# ELN orchestrates prometastatic and immunosuppressive niche in bladder cancer via TGFB1 autocrine signaling

**DOI:** 10.1172/jci.insight.194700

**Published:** 2026-05-22

**Authors:** Wentao Xu, Jia Gao, Shanshan Wu, Jianshang Huang, Chenchen An, Chonggui Jiang, Nianping Liu, Chen Cheng, Zihan Wang, Zijian Dong, Yuchen Xu, Jun Zhou, Hanren Dai, Xiaolei Li, Honghai Xu, Songyun Zhao, Qianwen Fan, Yang Li, Ying Dai, Li Zuo, Hua Wang

**Affiliations:** 1 Department of Oncology, the First Affiliated Hospital of Anhui Medical University, Hefei, China.; 2 Key Laboratory of Anti-inflammatory and Immune Medicine (Anhui Medical University), Ministry of Education, Hefei, China.; 3 Inflammation and Immune Mediated Diseases Laboratory of Anhui Province and; 4 Innovation and Entrepreneurship Laboratory for College Students, Anhui Medical University, Hefei, China.; 5 Department of Gastroenterology, The First Affiliated Hospital of Anhui Medical University, Hefei, China.; 6 Laboratory of Molecular Biology, and Department of Biochemistry, School of Basic Medical Science, Anhui Medical University, Hefei, China.; 7 Genomics and Bioinformatics at Division of Life Sciences and Medicine, USTC, Hefei, China.; 8 Department of Urology and; 9 Department of Pathology, The First Affiliated Hospital of Anhui Medical University, Hefei, China.; 10 Department of Plastic Surgery, The First Affiliated Hospital of Wenzhou Medical University, Wenzhou, China.; 11 Department of Genetics, School of Life Science, Anhui Medical University, Hefei, China.

**Keywords:** Immunology, Oncology, Cancer immunotherapy

## Abstract

Bladder cancer (BCa) mortality is mainly driven by metastatic dissemination and an immunosuppressive tumor microenvironment. Here, we identify ELN (tropoelastin), an extracellular matrix protein abundantly secreted by cancer-associated fibroblasts (CAFs), as a critical determinant of these processes and a marker of poor prognosis. ELN promotes epithelial-mesenchymal transition (EMT), facilitates lymphatic spread, and induces immune dysfunction characterized by macrophage polarization toward an M2 phenotype and T cell exhaustion. Mechanistically, ELN functions as a binding partner of TGF-β receptor 2 (TGFBR2), thereby triggering SMAD2/3-dependent TGF-β1 secretion and establishing a feed forward signaling loop. This ELN/TGFBR2/TGF-β1 axis amplifies metastatic capacity and immunosuppressive signaling, ultimately accelerating disease progression and diminishing responsiveness to immune checkpoint blockade. Functional studies in BCa organoids and murine models demonstrated that pharmacologic blockade of the ELN-TGFBR2 interaction effectively suppressed tumor metastasis and restored antitumor immunity. Collectively, our findings establish ELN as a CAF-derived driver of metastasis and immune evasion in BCa. Targeting the ELN-TGFBR2 interaction offers a promising therapeutic strategy to limit metastatic progression and enhance the efficacy of immunotherapy in this lethal disease.

## Abstract 2

This study reveals that ELN promotes bladder cancer progression by triggering TGFB1 signaling, which suppresses the immune system and drives tumor metastasis.

## Graphical abstract

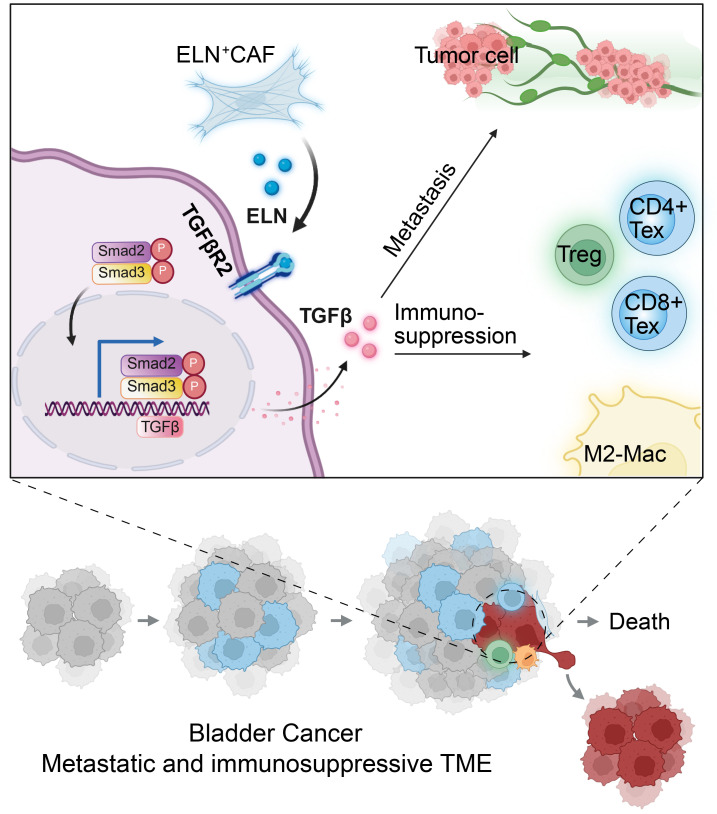

## Introduction

Bladder cancer (BCa) remains a major health burden worldwide, ranking among the most common malignancies of the urinary tract. Clinically, BCa is categorized into nonmuscleinvasive (NMIBC) and muscleinvasive (MIBC) forms, with MIBC showing a higher propensity for metastasis, immunosuppression and death (1). Although Bacillus Calmette-Gue′rin (BCG) intravesical (i.b.) immunotherapy has been used in BCa treatment for more than 4 decades, and BCa was among the earliest tumor types to receive FDA-approved immunotherapy, approximately 75% of patients fail to respond to BCG, and only 15%–25% derive durable benefit from immune checkpoint inhibitors (ICIs) (2, 3). These limitations highlight the pressing need to identify additional therapeutic targets in BCa.

Secreted proteins, including cytokines, growth factors, and soluble enzymes, serve as central mediators of intercellular communication and regulate a wide range of processes such as cell-to-cell signaling and immune responses, which has made them prominent therapeutic targets across multiple diseases, including cancer (4). However, secreted proteins exhibit remarkable diversity in their origins and destinations, such as those released into the circulation, acting within specific organs or systems, or deposited within the extracellular matrix (ECM) (5). This functional and spatial heterogeneity has motivated comprehensive studies of secreted proteins originating from distinct cellular or tissue compartments (6, 7), underscoring that different classes of secreted proteins may exert different biological effects. A prominent example is bevacizumab, an antibody against VEGFA, which suppresses tumor angiogenesis by restricting nutrient and oxygen supply. However, its therapeutic efficacy varies considerably among tumor types; for instance, the limited therapeutic response in pancreatic cancer underscores the organ-specific context and the inherent complexity of secreted-protein function (8).

Increasing evidence highlights the tumor microenvironment (TME) as a critical determinant of BCa progression, particularly the composition and remodeling of the ECM (9, 10). The ECM is a highly organized fibrous and gel-like network composed of proteins secreted into the extracellular space and polysaccharides, which provides mechanical scaffolding and regulates cellular behavior through diverse biochemical and biophysical cues that modulate proliferation, migration, and immune infiltration (11). Despite considerable advances in systemic therapy and immunotherapy, the secretome landscape in ECM of BCa and its contribution to tumor aggressiveness and immune evasion remain poorly defined. Unraveling these mechanisms may reveal new molecular determinants and therapeutic vulnerabilities specific to BCa.

Among the signaling pathways influenced by the ECM-associated secretome, the TGF-β1 (*TGFB1*) pathway has emerged as a central mediator of tumor progression. The TGF-β1 signaling pathway is abundantly active within the TME, where it orchestrates immune modulation, tumorigenesis, and metastatic progression (12). Signal transduction of TGF-β1 occurs through the activation of TGFB receptor type II (*TGFBR2*) and type I (*TGFBR1*), which phosphorylate SMAD*2/3* and drive their nuclear translocation to regulate transcriptional programs associated with malignant phenotypes in BCa (13). Previous studies have demonstrated that TGF-β1 is indispensable for fibroblast activation and ECM remodeling, while the bioavailability of TGF-β1 itself is tightly controlled by the ECM—stiffer matrices lower its activation threshold and amplify signaling (14). However, the mechanisms by which ECM-associated secreted proteins modulate tumor immunity and metastasis through the induction and activation of TGF-β1 remain poorly understood.

In this work, we identified ELN as a key modulator of the TGF-β1 signaling network in BCa. Expression of ELN is elevated in metastatic lesions and correlates with poor response to immunotherapy. We demonstrate that ELN directly binds to TGFBR2 to potentiate SMAD2/3 phosphorylation and amplify autocrine TGF-β1 signaling, thereby enhancing EMT and immunosuppressive phenotypes. Our findings define a previously unrecognized mechanism by which ELN amplifies TGF-β1–dependent tumor progression and immune evasion and point to the ELN-TGFBR2 interaction as a potential therapeutic target in advanced BCa.

## Results

### ELN correlates poor clinical outcomes in patients with BCa.

**Figure 1:**
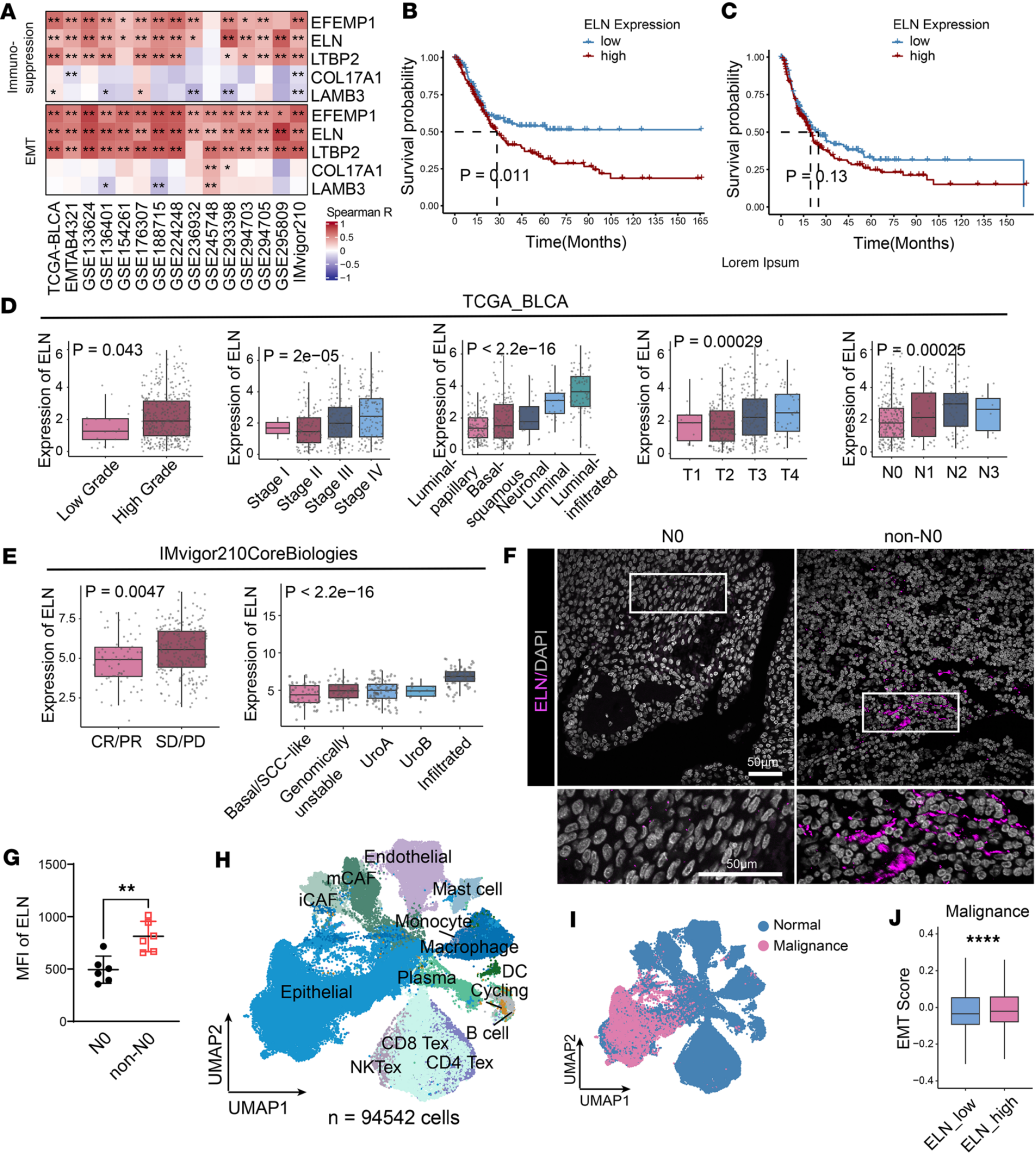
*ELN* expression correlates with BCa progression and poor clinical outcomes. (**A**) Heatmap shows the Spearman correlation between the expression of 5 candidate genes and signature scores for immunosuppression (top) and EMT (bottom) across multiple BCa high-throughput sequencing datasets. Correlation coefficients and *P* values were determined using Spearman’s correlation analysis. (**B** and **C**) Kaplan-Meier analysis of OS (**B**) and PFS (**C**) in TCGA-BLCA cohort (ELN_high = 204, ELN_low = 204). *P* values were determined by log-rank test. (**D**) *ELN* expression in primary tumors stratified by clinicopathological features in the TCGA-BLCA cohort, including tumor grade, clinical stage, molecular subtype, T stage, and N stage. *P* values were determined by Kruskal-Walli’s test. (**E**) *ELN* expression stratified by immunotherapy response and molecular subtype in the IMvigor210 cohort (*n* = 298). *P* values were determined by 2-tailed Mann-Whitney *U* test (left) and Kruskal-Wallis test (right). (**F**) Representative immunofluorescence staining of ELN (magenta) in metastatic (non-N0) and nonmetastatic (N0) BCa tissues. Nuclei were counterstained with DAPI (gray). Scale Bar: 50 μm. (**G**) Quantification of mean fluorescence intensity (MFI) of ELN in N0 versus non-N0 tissues. *n* = 6 for each group. *P* value was determined by 2-tailed Student’s *t* test. (**H**) UMAP visualization of 94,542 single cells from 12 human BCa tissues, annotated by major cell populations. (**I**) UMAP plot displaying cell origins grouped by status of epithelial cells (normal vs. malignant). (**J**) Quantitative comparison of EMT signature scores in malignancies between ELN_high and ELN_low groups. *P* value was determined by 2-tailed Mann-Whitney *U* test. Data are shown as mean ± SEM. **P < 0.01, ****P < 0.0001.

To identify secreted mediators that couple metastatic and immunosuppressive progression across cancers, we used a pancancer secretome analysis and identified ECM-secreted proteins as the top adverse prognostic module across solid tumors in TCGA (Supplemental Figure 1A; supplemental material available online with this article; https://doi.org/10.1172/jci.insight.194700DS1). Notably, BCa displayed the highest correlation between ECM-secretome signatures, EMT, and immunosuppression, indicating a distinct ECM-driven immune escape mechanism in this cancer type (Supplemental Figure 1B). To identify the key mediators, we intersected these ECM-secreted proteins with proteomic profiles of BCa progression and differentially expressed genes (DEGs) between anti–PD-L1 responders and nonresponders in the IMvigor210 cohort (15, 16). This screening strategy yielded 5 candidate genes (Supplemental Figure 1C). Multidataset correlation screening revealed that, among the identified candidate ECM-secreted proteins, ELN exhibited the most robust and consistent positive associations with both EMT and immunosuppression signatures across 15 independent BCa cohorts (Figure 1A). Additionally, ELN expression also stratified patients by overall survival (OS) in TCGA and progression-free survival (PFS) in EMTAB1803 dataset (Figure 1, B and C, and Supplemental Figure 1D). Pancancer univariate Cox analysis further identified ELN as a robust risk factor for both OS and PFS across multiple TCGA malignancies, including BCa (Supplemental Figure 2, A and B).

Consistent with these findings, expression of *ELN* was significantly increased with grade, tumor stage, terminal molecular subtype, lymph node status, recurrent, and advanced lesions in multiple independent cohorts (Figure 1D and Supplemental Figure 3A). Additionally, high *ELN* levels were enriched in anti-PDL1 metastatic nonresponders (Figure 1E). We also performed immunofluorescence staining to analyze intense ELN deposition in metastatic BCa tumors compared with lymph node negative controls (Figure 1, F and G).

To further delineate the relationship between *ELN* and the TME, we performed single-cell RNA-seq on tumor samples from 4 patients with BCa and integrated these data with the dataset of 8 additional data supported by Chen et al. (17). The combined analysis yielded 94,542 cells, which were classified into 14 distinct cellular clusters encompassing epithelial, stromal, and immune compartments (Figure 1H and Supplemental Figure 4A). Interestingly, at the highest clustering resolution, all T cell subpopulations expressed exhaustion associated markers; therefore, T cells were further categorized into exhausted CD4^+^ T cells (CD4^+^ Tex), CD8^+^ Tex, and exhausted NKT cells (NKTex) subsets (Supplemental Figure 4B). Based on *ELN* expression, the 12 patient samples were stratified into *ELN*_high and *ELN*_low groups (Supplemental Figure 4, C and D). Using inferCNV to infer genomic copy number variation within epithelial clusters, we delineated malignances in scRNA data. scRNA-seq analysis revealed that malignances in *ELN*_high samples exhibited enriched EMT signatures, consistent with the findings from the TCGA-BLCA cohort (Figure 1, I and J; Supplemental Figure 3B; and Supplemental Figure 4E).

Collectively, these data confirm that increased expression of *ELN* in BCa correlates strongly with poor clinical outcomes.

### ELN is a CAF-secreted driver of EMT in BCa.

**Figure 2:**
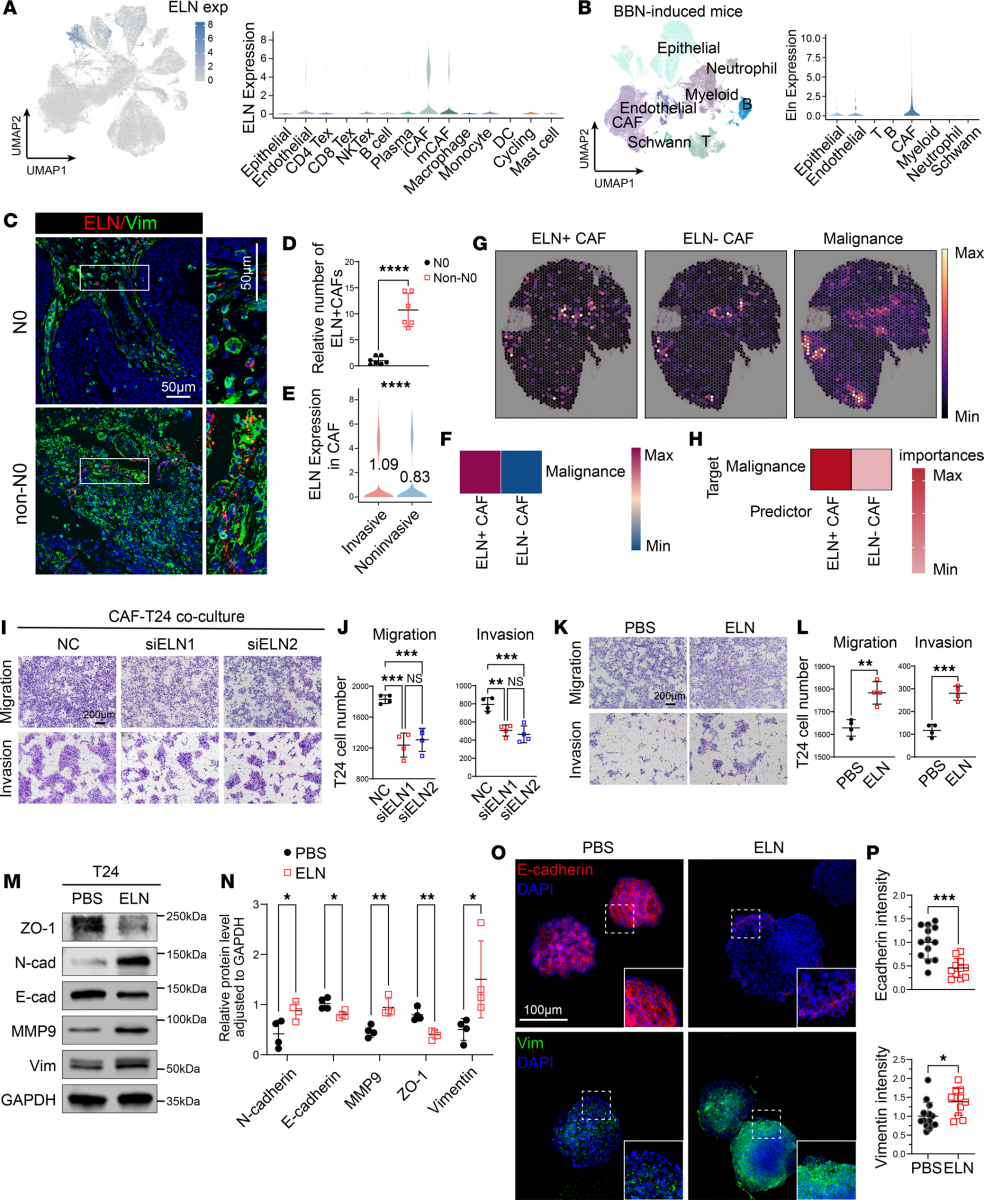
ELN^+^ CAFs promotes metastatic niche in BCa. (**A**) *ELN* expression across cell populations in human BCa (right). (**B**) UMAP plot of major cell populations (left) and *ELN* expression in BBN-induced mouse BCa model. (**C**) Representative immunofluorescence staining of ELN (red) and Vimentin (green) in nonmetastatic (N0) and metastatic (non-N0) human BCa tissues. Insets highlight ELN^+^ CAFs in metastatic lesions. Scale bar: 50 μm. (**D** and **E**) Quantification of ELN^+^ CAF numbers in N0 versus non-N0 tissues (**D**, *n* = 6 for each group), and *ELN* expression levels in CAFs between invasive and noninvasive groups from scRNA-seq data (**E**). *P* values were determined by 2-tailed Student’s *t* test (**D**) or 2-tailed Mann-Whitney *U* test (**E**). (**F**) Cell-cell interactions between malignancies and CAFs using CellPhoneDB. (**G** and **H**) Spatial transcriptomic deconvolution (**G**) and median importance of cell-type abundance in predicting the proximity of CAFs and malignancies within a spatial spot (**H**). (**I**) Representative images of migration and invasion assays for T24 cells cocultured with control (NC) or *ELN*-silenced (siELN) CAFs. (**J**) Quantitative analysis of migrated and invaded T24 cells. *n* = 4 for each group. *P* values were determined by 1-way ANOVA followed by Tukey’s multiple-comparison test. (**K** and **L**) Migration and invasion assays of T24 cells treated with PBS or rm-ELN (**K**) and corresponding quantification (**L**). *P* values were determined by 2-tailed Student’s *t* test. (**M** and **N**) Immunoblot analysis (**M**) and quantification (**N**) of EMT markers and MMP9 in T24 cells treated with rm-ELN. *n* = 3 for each group. *P* values were determined by 2-tailed Student’s *t* test. (**O** and **P**) Immunofluorescence staining of E-cadherin (red) and Vimentin (green) in mouse BCa organoids (**O**) and corresponding quantification of fluorescence intensity (**P**). Scale bar: 100 µm. *P* values were determined by 2-tailed Student’s *t* test. Data are shown as mean ± SEM. *P < 0.05, **P < 0.01, ***P < 0.001, ****P < 0.0001.

Single-cell transcriptomic analysis pinpointed CAFs as the predominant source of *ELN* within the BCa microenvironment (Figure 2A). We next analyzed scRNA-seq data from the BBN-induced murine BCa model (18), which similarly demonstrated that *Eln* expression is predominantly enriched in CAFs (Figure 2B and Supplemental Figure 4F). This indicates that the cellular origin of *ELN* is evolutionarily conserved across species in BCa. Analysis of human BCa specimens revealed a marked increase in ELN^+^ CAF abundance in lymph node–positive tumors compared with node-negative cases (Figure 2, C and D), and *ELN* expression was further elevated in invasive molecular subtypes compared with noninvasive lesions, as evidenced by our human BCa scRNA-seq data (Figure 2E). To investigate the clinical and biological significance of *ELN* expression within CAFs, we classified scRNA–derived CAFs into ELN^+^ CAFs and ELN^–^ CAFs based on *ELN* transcript counts (*ELN* > 0 versus = 0). Using BayesPrism deconvolution, these CAF signatures were subsequently projected onto the TCGA-BLCA bulk RNA-seq dataset. Patients with high proportions of ELN^+^ CAFs exhibited significantly worse OS (Supplemental Figure 5A), and ELN^+^ CAF abundance increased with tumor grade, stage, lymph-node metastasis, and advanced molecular subtypes (Supplemental Figure 5, B–F). We also performed CellPhoneDB to analyze extensive ligand-receptor interactions between CAFs and malignances, further supporting active paracrine communication within ELN^+^ CAFs and malignances (Figure 2F). Consistently, spatial deconvolution of transcriptomic profiles revealed that regions coenriched for CAF signatures were predominantly distributed within malignances (Figure 2G and Supplemental Figure 6, A–D). MistyR analysis further demonstrated that ELN^+^ CAFs were positioned in closer spatial proximity to malignances (Figure 2H).

Next, we isolated primary CAFs from human BCa tissues by flow cytometry, gating on viable EPCAM^–^CD45^–^PDPN^+^PDGFRA^+^ cells (Supplemental Figure 7A). To assess the functional role of *ELN* in these stromal cells, we used RNA interference to knockdown *ELN* expression with 2 independent siRNAs (siELN1 and siELN2). Both constructs efficiently reduced *ELN* levels, as evidenced by diminished *ELN* immunofluorescence signals in vimentin positive CAFs (Supplemental Figure 7, B and C) and markedly decreased protein expression on immunoblot analysis (Supplemental Figure 7, D and E). Functionally, silencing *ELN* in primary human BCa CAFs markedly suppressed migration and invasion of T24 BCa cell line in transwell assays (Figure 2, I and J). Conversely, recombinant ELN (rm-ELN) recapitulated these proinvasive effects, significantly enhancing cancer cell motility and invasiveness (Figure 2, K and L). Western blotting confirmed that rm-ELN treated BCa cell line displayed reduced epithelial markers (E-cadherin, ZO-1) and increased mesenchymal and matrix remodeling proteins (N-cadherin, Vimentin, MMP9) (Figure 2, M and N). Immunofluorescence further demonstrated cytoskeletal rearrangement and the characteristic gain of vimentin accompanied by loss of E-cadherin after rm-ELN exposure in mouse BCa organoids (Figure 2, O and P).

Together, these findings demonstrate that CAF-derived ELN is a key stromal driver of EMT and invasive behavior in BCa.

### ELN promotes immunosuppressive cell states in BCa.

**Figure 3:**
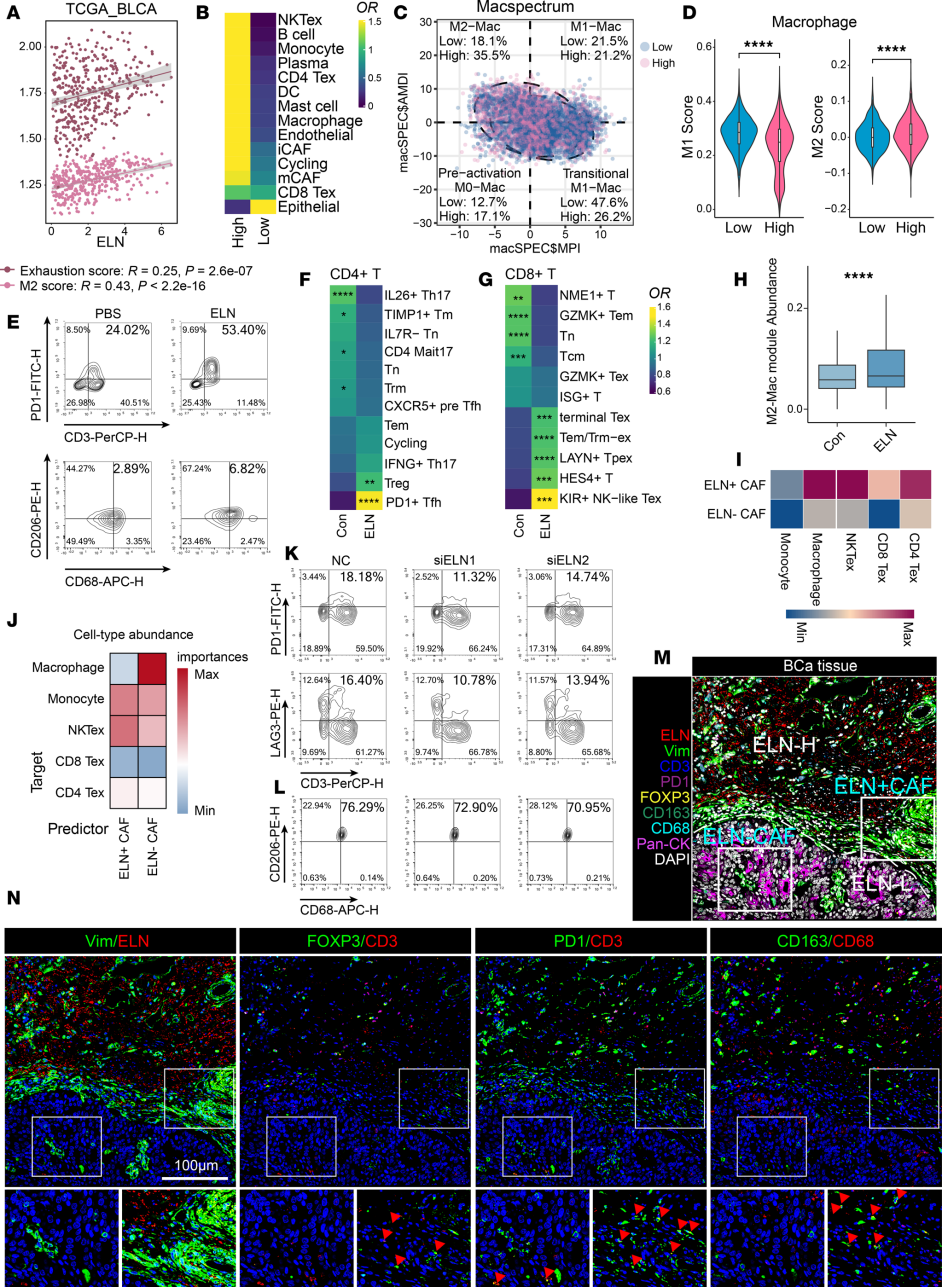
ELN^+^ CAFs shape an immunosuppressive microenvironment characterized by T cell exhaustion and M2 macrophage polarization. (**A**) Scatter plots showing positive correlations between *ELN* expression and exhaustion scores, as well as M2 polarization scores in the TCGA-BLCA dataset. Spearman correlation was used. (**B**) Heatmap displaying the ORs of various cell types between *ELN*_high and *ELN*_low groups. (**C**) MacSpectrum analysis of monocytes and macrophages in *ELN*_high and *ELN*_low groups. (**D**) Violin plots comparing M1 and M2 scores between *ELN*_high and *ELN*_low groups. *P* values were determined by 2-tailed Mann-Whitney *U* test. (**E**) Representative flow-cytometric plots of PBMCs cocultured with PBS or rm-ELN. (**F** and **G**) Heatmaps showing the ORs of CD4^+^ T and CD8^+^ T cell subsets in PBS and rm-ELN groups. (**H**) Abundance of M2-like macrophage modules in PBS and rm-ELN groups. *P* value was determined by 2-tailed Mann-Whitney *U* test. (**I**) Cell-cell interaction analysis between CAFs and immune cell populations using CellPhoneDB. (**J**) Median importance of cell-type abundance in predicting the proximity of CAFs and immune cells within a spatial spot. (**K** and **L**) Representative flow-cytometric analysis of PD-1^+^ and LAG3^+^ T cells (**K**) or CD206^+^ macrophages (**L**) cocultured with NC or *ELN*-silenced CAFs. (**M** and **N**) Multiplex immunofluorescence staining of human BCa tissues showing spatial proximity of ELN^+^ CAF regions (ELN^+^Vimentin^+^, red/green) to immunosuppressive populations including Tregs (FOXP3^+^), PD-1^+^CD3^+^ T cells, and CD163^+^ macrophages. Red arrows highlight the specific colocalization. Scale bar: 100 µm. Data are shown as mean ± SEM. *P < 0.05, **P < 0.01, ***P < 0.001, ****P < 0.0001.

Given the strong association of *ELN* with nonresponse to anti–PD-1 immunotherapy, we next investigated its effect on the immune microenvironment of BCa. In the TCGA-BLCA dataset, *ELN* expression correlated negatively with tumor mutational burden (TMB), suggesting that *ELN*_high tumors harbor fewer neoantigens and may be less immunogenic (Supplemental Figure 8A). Consistently, high *ELN* expression in BCa was strongly associated with an immunosuppressive TME, characterized by elevated M2-macrophage and Tex signatures as well as canonical M2 and Tex marker genes (Figure 3A and Supplemental Figure 8, B–D).

Further analysis of human BCa scRNA data revealed that *ELN*_high group displayed a broadly cold tumor phenotype, marked by increased presence of immunosuppressive lineages, such as CD4^+^ Tex, CD8^+^ Tex, NKTex, and M2-macrophages (Figure 3, B and C). Moreover, macrophages profiling demonstrated a loss of M1 and enrichment of M2 signature in *ELN*_high group, indicative of immunosuppressive polarization (Figure 3D).

In human peripheral blood mononuclear cells (PBMCs), treatment with rm-ELN led to a significant increase in PD-1^+^ T cell and CD206^+^ macrophages compared with controls (Figure 3E and Supplemental Figure 9A). To further assess the direct effect of *ELN* on immune cells, we performed droplet based scRNA on human PBMCs stimulated with rm-ELN. UMAP embedding identified major immune cell lineages, including T cells, B cells, NK cells, monocytes, and macrophages (Supplemental Figure 9, B and C). Within the T cell compartment, refined clustering delineated 12 distinct CD4^+^ and 11 CD8^+^ T cell states (Supplemental Figure 9, D–F). From the odds ratio (OR) analysis, exposure to rm-ELN led to an increased abundance of Tex, including Treg, PD-1^+^ Tfh, terminal Tex, Tem/Trm-ex, LAYN^+^ Tpex, KIR^+^ NK-like Tex (Figure 3, F and G). Considering the marked heterogeneity within macrophages populations, we applied nonnegative matrix factorization (NMF) metaprogram analysis, which revealed an expansion of M2-like macrophages clusters characterized by elevated expression of *CD163*, *MMP9*, *MAFB*, and *CLEC4E* (Supplemental Figure 9G and Supplemental Table 1). Consistent with the in vivo correlation analyses, MP1 was markedly more abundant following rm-ELN stimulation (Figure 3H).

Building on these findings, we next analyzed how ELN producing CAFs shape immune cell organization and function within the TME. In TCGA-BLCA data, correlation analyses further revealed that ELN^+^ CAFs were preferentially associated with immunosuppressive immune signatures, characterized by strong positive correlations with Tex markers (*HAVCR2*, *LAG3*, *PDCD1*, and *CTLA4*), M2-like macrophage markers (*CD163*, *MRC1*, *IL10*), as well as increased proportions of NKTex and myeloid cell subsets (Supplemental Figure 8, E and F). CellPhoneDB analysis of human BCa scRNA revealed that ELN^+^ CAFs displayed stronger ligand–receptor interactions with CD4^+^ Tex, CD8^+^ Tex, NKTex, monocyte and macrophages (Figure 3I). Spatial transcriptomic mapping further demonstrated that ELN^+^ CAFs were positioned in closer proximity to Tex and monocyte (Figure 3J). It is worth noting that macrophages represent a terminally differentiated state with limited plasticity, whereas monocytes retain higher differentiation potential. The closer spatial proximity of monocytes to ELN^+^ CAFs, therefore, suggests that ELN^+^ CAFs may drive monocyte differentiation toward immunosuppressive macrophages. These data suggest that ELN strengthen communicative interactions between CAFs and Tex or monocyte/macrophage lineage, thereby fostering an immunosuppressive TME. To determine the direct effect of ELN in CAFs on immune modulation, in the absence of ELN, CAFs significantly reduced the induction of PD-1^+^ and LAG3^+^ T cell subsets in CD3^+^ T cell cocultures and decreased CD206^+^ macrophages polarization in monocyte derived macrophage cultures (Figure 3, K and L, and Supplemental Figure 9H). Multiplex immunofluorescence analysis of human BCa further confirmed that ELN_high stromal regions were spatially associated with FOXP3^+^ and PD-1^+^ T cells as well as CD163^+^ macrophages (Figure 3, M and N, and Supplemental Figure 10, A and B).

These results suggest a central role of ELN in maintaining immunosuppressive landscapes in BCa.

### ELN activates TGF-β1 signaling in tumor cells and enhances the immunosuppressive function of immune cells.

**Figure 4:**
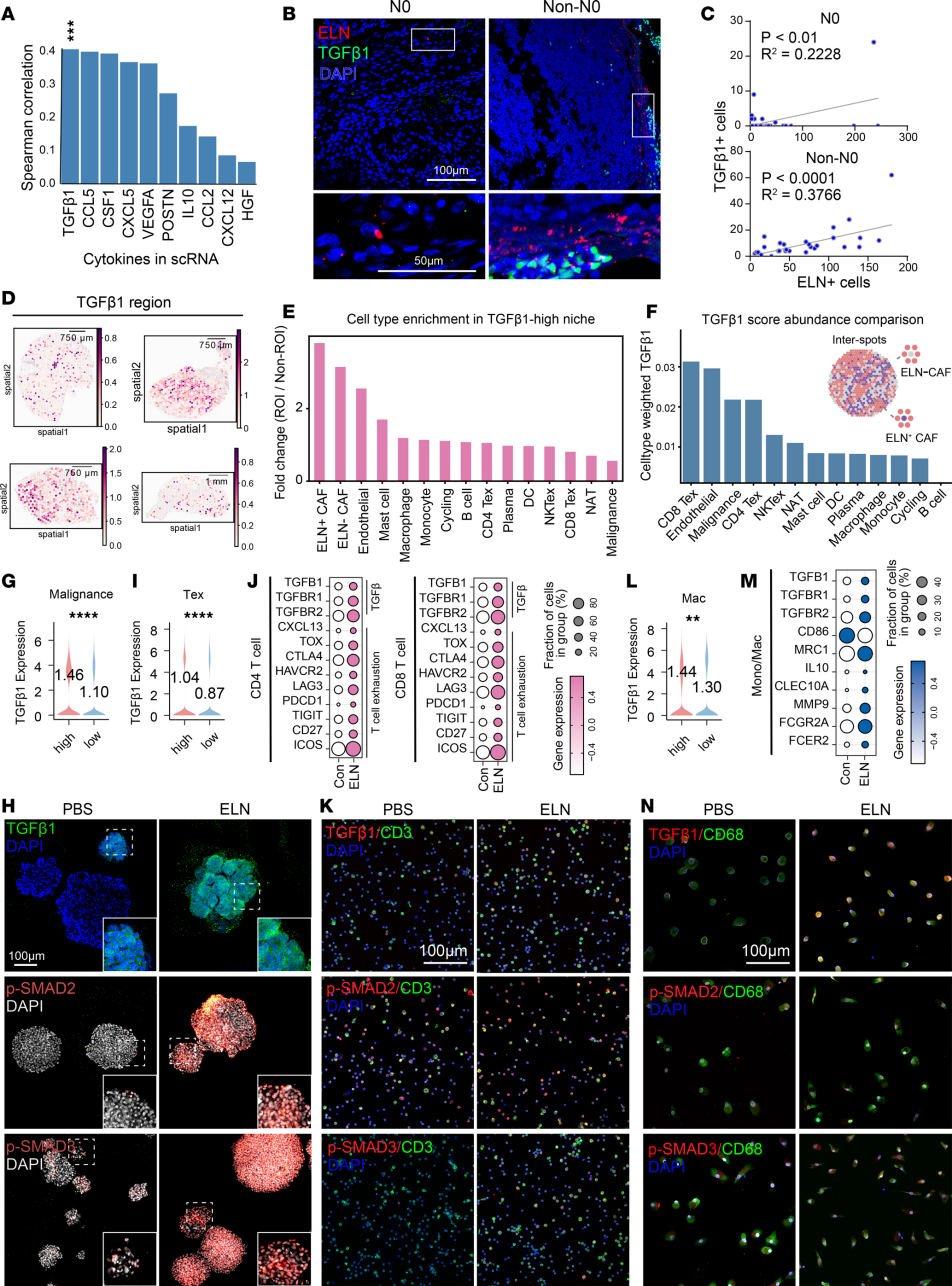
ELN activates TGF-β1 signaling in tumor cells, T cells, and macrophages. (**A**) Spearman correlations between *ELN* and cytokine transcripts across human BCa scRNA-seq data. (**B**) Representative immunofluorescence images showing the spatial relationship between ELN (red) and TGF-β1 (green) in N0 and non-N0 BCa tissues. Scale bar: 100 μm and 50 μm (insets). (**C**) Correlation analysis between ELN^+^ cells and TGF-β1^+^ cells in N0 (*n* = 6) and non-N0 (*n* = 6) tissues. *R*^2^ and *P* values were determined by linear regression analysis. Each data point represents a single field of view (FOV) from clinical samples. (**D**) Spatial transcriptomic maps identifying *TGFB1*-related regions across multiple tumor sections. (**E**) Fold-change enrichment of cell types within TGFB1-high regions (ROI) versus non-ROI areas. (**F**) Cell-type weighted *TGFB1* score abundance comparison between ELN^+^ CAF and ELN^–^ CAF dominated niches. (**G**) *TGFB1* expression in malignancies stratified by *ELN*_high and *ELN*_low groups. *P* value was determined by 2-tailed Mann-Whitney *U* test. (**H**) Immunofluorescence images of TGF-β1, p-SMAD2, and p-SMAD3 in human BCa organoids treated with PBS or rm-ELN. (**I**) *TGFB1* expression in Tex stratified by *ELN*_high and *ELN*_low groups. *P* values were determined by 2-tailed Mann-Whitney *U* test. (**J**) Dot plots showing transcriptional levels of TGF-β1–related signaling components and exhaustion-associated markers in CD4^+^ and CD8^+^ T cells from PBS- or rm-ELN–treated PBMCs. (**K**) Immunofluorescence staining of TGF-β1, p-SMAD2, and p-SMAD3 in purified CD3^+^ T cells following PBS or rm-ELN exposure. (**L**) *TGFB1* expression in macrophages stratified by *ELN*_high and *ELN*_low groups. *P* values were determined by 2-tailed Mann-Whitney *U* test. (**M**) Transcriptional levels of TGF-β1–related signaling components and macrophage-associated markers in monocytes/macrophages from PBS- or rm-ELN–treated PBMCs. (**N**) Immunofluorescence staining of TGF-β1, p-SMAD2, and p-SMAD3 in purified CD68^+^ macrophages following PBS or rm-ELN exposure. Scale bar: 100 µm. Data are shown as mean ± SEM. **P < 0.01, ***P < 0.001, ****P < 0.0001.

To further elucidate the signaling mechanisms underlying the prometastatic and immunosuppressive effects of *ELN*, we evaluated pertinent cytokine activity profiles across the human scRNA data in BCa. Among secreted factors correlated with *ELN* expression, *TGFB1* showed the strongest positive association, followed by *CCL5*, *CSF1*, *CXCL5*, and *VEGFA* (Figure 4A). Immunofluorescence analysis revealed a significant positive correlation between ELN and TGF-β1 expression in BCa tissues, with metastatic samples exhibiting a stronger coupregulation (Figure 4, B and C). Additionally, using the spatial transcriptomic framework described by Teichmann et al. (19), we next delineated *TGFB1*-defined regions within BCa tissues and observed that ELN^+^ CAFs were most strongly enriched within these *TGFB1*^hi^ niches. This spatial colocalization suggests that ELN^+^ CAFs may play a key role in organizing or reinforcing the *TGFB1*-dependent ecological niche within the BCa microenvironment (Figure 4, D and E). Building upon this spatial organization, we further examined how ELN^+^ and ELN^–^ CAFs regions differ in their local TGF-β1 signaling landscape. By integrating deconvolution-derived abundances with spot-level TGF-β1 scores, we computed cell-type–specific weighted *TGFB1* contributions and compared their distributions between ELN^+^ and ELN^–^ CAFs interspots. Across multiple BCa samples, regions surrounding ELN^+^ CAFs exhibited higher weighted *TGFB1* activity than areas adjacent to ELN^–^ CAFs, with the strong contributions arising from CD8^+^ Tex, malignances, CD4^+^ Tex, NKTex and monocyte/macrophage populations in this order (Figure 4F). These patterns suggest that ELN^+^ CAFs may drive elevated TGF-β1 production within their local microenvironment. Notably, TGF-β1 response scores were significantly higher in malignances and multiple immune lineages such as macrophages, NKTex, CD4^+^ Tex compared with noninvasive counterparts (Supplemental Figure 11A). Consistently, *TGFB1* expression was enriched in malignances, normal adjacent tissue (NAT), stromal, and immune populations, including macrophages and Tex (Supplemental Figure 11B). These analyses reveal a broad activation of TGF-β1 signaling in both malignant and immune compartments, supporting its possible role as a key mediator of ELN in both tumor and immune cells. Together, these findings support a model in which ELN^+^ CAFs and their associated stromal networks help structure or potentiate TGF-β1–driven metastatic and immunoregulatory niches within BCa tissues.

At the single-cell level malignances from *ELN*_high patients exhibited markedly elevated *TGFB1* levels (Figure 4G), and immunofluorescence showed stronger TGF-β1, p-SMAD2, and p-SMAD3 signals in mice BCa organoids treated with rm-ELN compared with controls (Figure 4H and Supplemental Figure 11C). In addition to elevated *TGFB1* levels in tumor cells, *TGFB1* expression was also upregulated in Tex cells from human BCa samples (Figure 4I). Consistent with this, both CD4^+^ and CD8^+^ T cells from rm-ELN treated PBMCs exhibited increased transcription of TGF-β1 signaling components and Tex related genes (Figure 4J). In vitro, exposure to rm-ELN intensified TGF-β1, p-SMAD2, and p-SMAD3 signals in CD3^+^ T cells (Figure 4K and Supplemental Figure 11D). Cui et al. previously reported an immune response dictionary at single-cell resolution detailing cytokine activity and immune cell polarization derived from gene expression data (20). We utilized this dictionary and found that transcriptional profiles post-ELN treatment was biased toward T4-e, T4-f, T4-c, T8-b, and T8-f, all of which align with Treg cell polarization states (Supplemental Figure 12, A and B). Next, we examined the trajectories of T cell differentiation using gene expression data and inferred the enhanced paths of T cell exhaustion following ELN treatment based on RNA velocity, showing that naive T cell (Tn) populations flowed toward Tregs in CD4^+^ T cells and toward LAYN^+^ precursor exhausted T cells (Tpex) in CD8^+^ T cells (Supplemental Figure 12, C and D). We then traced the differentiation trajectories along these 2 paths, with CD4^+^ and CD8^+^ T cells exhibiting increased *TGFB1* and exhaustion activity (Supplemental Figure 12, E and F). ELN treatment substantially heightened the activity of both pathways, underscoring a crucial role for TGF-β1 in ELN-triggered immunosuppression (Supplemental Figure 12G).

Moreover, *TGFB1* expression was also upregulated in macrophages from human BCa samples, as well as macrophages from rm-ELN–treated PBMCs showed coordinated induction of TGF-β1 signaling, and M2-polarization markers (Figure 4, L–N, and Supplemental Figure 11E). Consistently, the cytokine response dictionary suggested that transcription by monocyte/macrophages post-ELN treatment shifted more toward the Mac-e polarization state, driven by classic M2 macrophage cytokines like IL-4 and IL-13 (Supplemental Figure 12H). pySCENIC analysis revealed relevant motifs after ELN treatment, such as the activation of M2 transcription factors (e.g., *SMAD2*, *FOS*, *EZH2*) and the attenuation of M1-polarization transcription factors (e.g., *STAT1*, *IRF1*) (Supplemental Figure 12I).

Together, these findings identify ELN as a key upstream regulator of TGF-β1 signaling that links tumor-cell metastasis, immune cell exhaustion and macrophage immunosuppressive polarization in BCa.

### ELN acts as a binding partner of TGFBR2.

**Figure 5:**
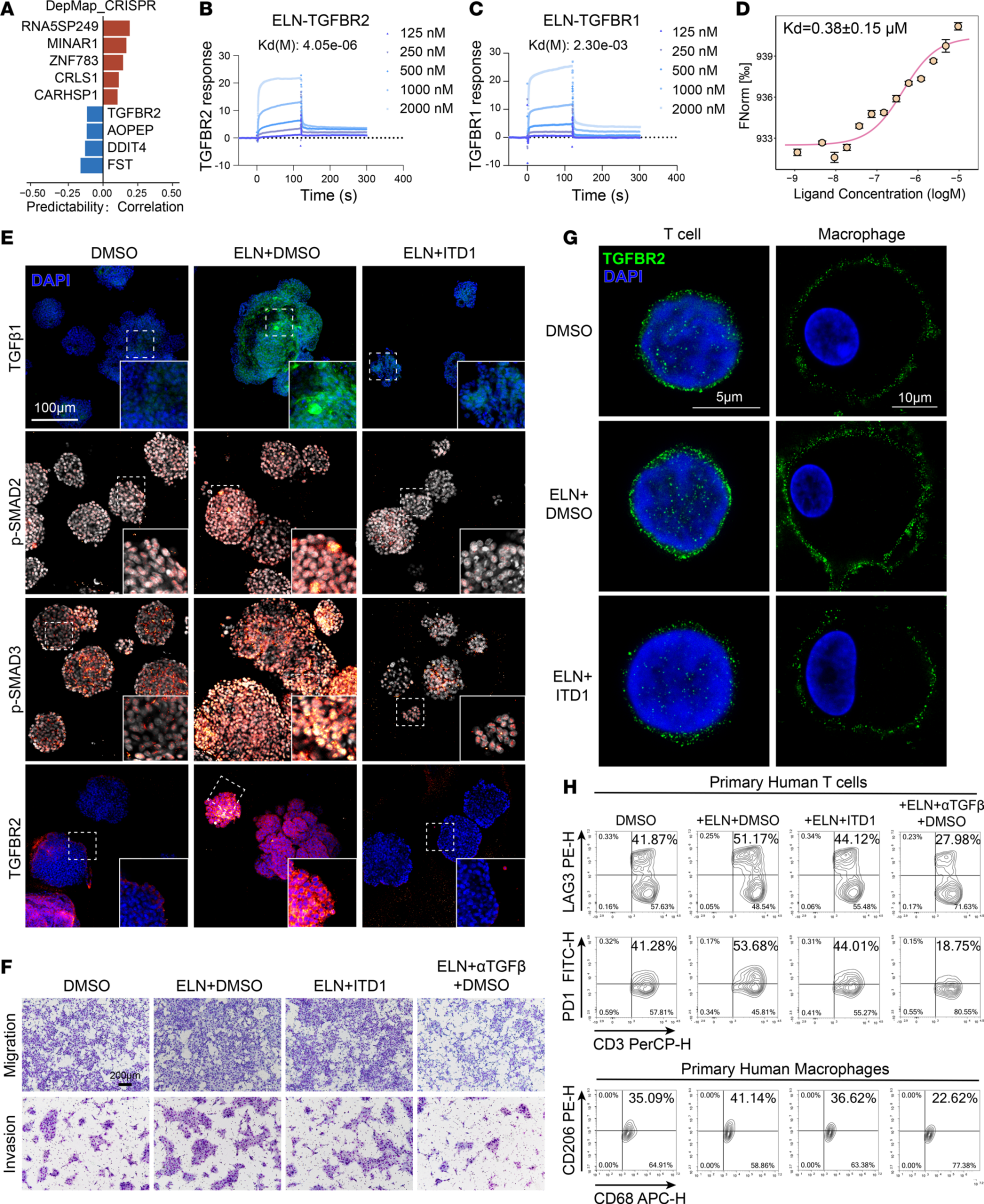
ELN directly binds TGFBR2 and activates TGF-β1 autocrine signaling in tumor cells and immune cells. (**A**) Correlation analysis of *ELN* dependency and CRISPR perturbation profiles across the DepMap Public 24Q4 dataset. Blue bars indicate genes, including *TGFBR2*, exhibiting significant functional codependency with ELN. (**B** and **C**) SPR sensorgrams showing binding kinetics of ELN to TGFBR2 (**B**) and TGFBR1 (**C**) at the indicated concentrations. (**D**) MST binding curve illustrating the direct interaction between ELN and TGFBR2. (**E**) Representative immunofluorescence images of TGF-β1, p-SMAD2, p-SMAD3, and TGFBR2 in human BCa organoids treated with DMSO, rm-ELN + DMSO, or rm-ELN + ITD1. Scale bar: 100 µm. (**F**) Representative images of migration and invasion assays for T24 cells under the indicated treatment conditions. (**G**) STED superresolution microscopy showing cell-surface TGFBR2 localization (green) in primary human T cells and macrophages. Scale bar: 5 μm (T cell) and 10 μm (macrophage). (**H**) Representative flow-cytometric plots of LAG3 and PD-1 in primary human T cells (top and middle panels) as well as CD206^+^ macrophages (bottom panel) following the indicated treatments. Numbers indicate the percentage of cells in each quadrant or gate.

After confirming that ELN can upregulate *TGFB1* and *SMAD2/3* levels in tumor cells, T cells and macrophages. We first interrogated the DepMap CRISPR dependency dataset (Version: DepMap Public 24Q4). Among the candidate genes associated with *ELN* dependency in the CRISPR perturbation screen, *TGFBR2* exhibited one of the strongest negative correlations (Figure 5A), indicating that perturb of *ELN* more profoundly affects cells with higher *TGFBR2* expression and suggesting an expression dependent relationship between *ELN* and *TGFBR2*.

Given that *SMAD2/3* activation itself can upregulate *TGFB1* expression (21, 22), we next investigated whether ELN acts as a ligand for TGFBR2 to initiate TGF-β1 signaling. Correlation analysis across the TCGA-BLCA cohort revealed that *ELN* expression exhibited the strongest positive association with *TGFBR2* among *TGFB1* superfamily members (Supplemental Figure 13A). Subsequently, we performed surface plasmon resonance (SPR) analyses using rm-ELN, which demonstrated specific high affinity binding to TGFBR2 with high affinity (Kd = 4.05 × 10^–6^ M), whereas its interaction with TGFBR1 was much weaker (Kd = 2.30 × 10^–3^ M) (Figure 5, B and C). We further measured the binding affinity of the TGF-β1–TGFBR2 positive control pair, which showed a higher affinity interaction (Kd = 9.904 × 10^–8^ M) (Supplemental Figure 13B). Microscale thermophoresis (MST) further confirmed the interaction between ELN and TGFBR2 (Kd = 0.38 ± 0.15 μM) (Figure 5D and Supplemental Figure 13, C and D).

Notably, TGFBR2 is broadly expressed across various cell populations in human BCa scRNA-seq data. CellPhoneDB analysis further revealed that ELN/TGFBR2 ligand-receptor interactions are significantly enriched in both T cells and macrophages (Supplemental Figure 13, E and F), highlighting these populations as potential ELN-responsive cells within the TME. Given prior evidence that ELN can drive EMT in colorectal cancer cells (23) and ELN can promote metastatic behaviors in BCa cells, we reasoned that BCa cells might likewise serve as an additional ELN-responsive population. Consistent with the binding evidence, CellPhoneDB analysis of our human BCa scRNA revealed robust customed ELN-TGFBR2 ligand–receptor interactions between CAFs and malignances (Supplemental Figure 13G). To validate this interaction in vitro, rm-ELN was applied to T24 cells and mouse BCa organoids. ELN treatment markedly enhanced expression of TGFBR2, autocrine TGF-β1 production and SMAD2/3 phosphorylation, confirming activation of the canonical *TGFB1* pathway and inducing EMT in organoids. These effects were abrogated by ITD1, a TGFBR2-specific inhibitor that removes TGFBR2 from the cell surface (Figure 5E; Supplemental Figure 14, A–E; Supplemental Figure 15, A and B; and Supplemental Figure 16, A and B). Mechanistically, both ITD1 and anti–TGF-β neutralizing antibody attenuated ELN-induced tumor-cell migration and invasion (Figure 5F and Supplemental Figure 16, C and D).

Extending this analysis to immune populations, CellPhoneDB predicted frequent ELN-TGFBR2 interactions between CAFs and Tex, as well as with monocyte/macrophage clusters (Supplemental Figure 13H), implying that ELN may engage TGFBR2 across multiple immune subsets. To validate these predictions, we examined the effect of rm-ELN on purified primary human T cells and monocyte derived macrophages in vitro. Exposure to rm-ELN upregulated TGFBR2 expression and TGF-β1 production as well as enhanced SMAD2/3 phosphorylation in both cell types, whereas ITD1 treatment abolished these changes (Supplemental Figure 15, C–F). Congruously, super resolution STED microscopy further demonstrated increased cell surface TGFBR2 localization in ELN treated T cells and macrophages (Figure 5G and Supplemental Figure 16E). ELN stimulation significantly elevated the proportions of LAG3^+^ and PD-1^+^ Tex as well as CD206^+^ M2 like macrophages; both ITD1 and anti–TGF-β neutralization effectively prevented these phenotypic changes (Figure 5H and Supplemental Figure 16, F and G).

Together, these results demonstrate that ELN directly engages TGFBR2 to activate SMAD2/3 signaling in BCa cells, T cells, and macrophages, thereby driving TGF-β1–dependent tumor metastasis and immune suppression.

### ELN/TGFBR2/TGF-β1 axis attenuates the capacity of immune cells to eliminate BCa organoids.

**Figure 6:**
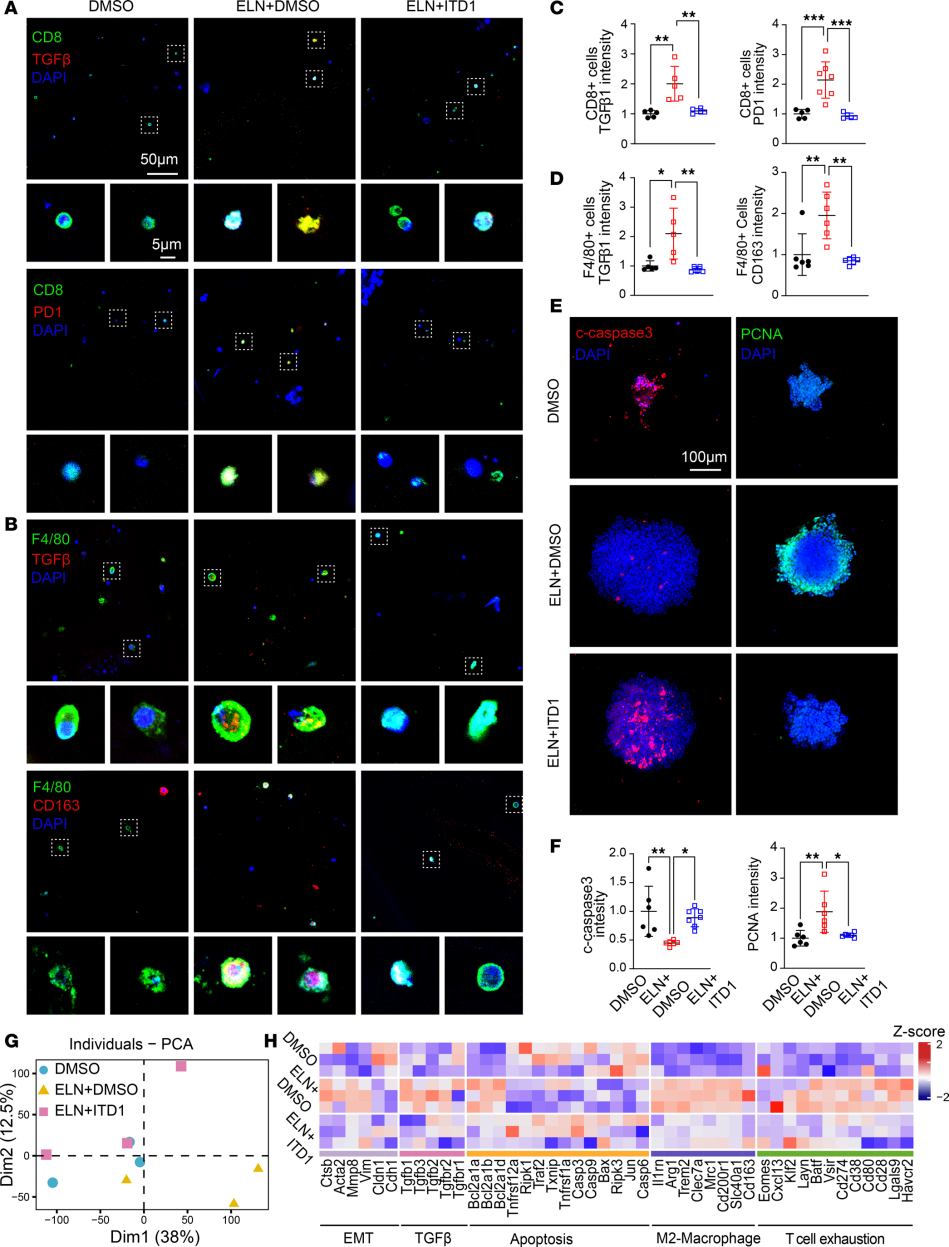
ELN promotes immunosuppressive immune cell subsets through TGF-β1 production. (**A** and **B**) Representative immunofluorescence images of CD8^+^ T cells (**A**) and F4/80^+^ macrophages (**B**) treated with DMSO, rm-ELN + DMSO, or rm-ELN + ITD1 within TIOs. Scale bar: 50 μm and 5 μm (insets). (**C** and **D**) Quantification of fluorescence intensity for TGF-β1 and PD-1 in CD8^+^ T cells (**C**), TGF-β1 and CD163 in F4/80^+^ macrophages (**D**). *n* = 5–7 for each group. *P* values were determined by 1-way ANOVA followed by Tukey’s multiple-comparison test. (**E**) Immunofluorescence staining of cleaved-caspase 3 and PCNA in TIOs across treatment groups. Scale bar: 100 μm. (**F**) Quantification of fluorescence intensity for cleaved-caspase 3 and PCNA in organoids. *n* = 5–7 for each group. *P* values were determined by 1-way ANOVA followed by Tukey’s multiple-comparison test. (**G**) PCA of transcriptional profiles from TIOs treated as indicated (*n* = 3 per group). (**H**) Heatmap displaying *z* scores of genes associated with EMT, TGF-β1 signaling, apoptosis, M2-macrophage polarization, and T cell exhaustion across treatment groups. Data are shown as mean ± SEM. *P < 0.05, **P < 0.01, ***P < 0.001.

We next investigated the impact of this axis within tumor immuno-organoids (TIOs) derived from mouse models, focusing on immune cell dynamics in response to ELN stimulation. The experimental framework consisted of BCa organoids cocultured with mesenteric lymph node–derived (MLN-derived) T cells and bone marrow–derived macrophages (BMDMs), treated with recombinant ELN and/or the TGFBR2 inhibitor ITD1 (Supplemental Figure 17A). Within this coculture system, rm-ELN treatment markedly enhanced organoid growth and metabolic activity while reducing organoid death after 48 hours (Supplemental Figure 17, B and C). Immunofluorescence analysis revealed heterogeneous TGF-β1 expression across immune cell populations. In particular, CD8^+^ T cells exposed to ELN exhibited elevated TGF-β1 and PD-1 expression, consistent with T cell exhaustion, whereas F4/80^+^ macrophages displayed increased TGF-β1 and CD163 levels as well (Figure 6, A–D). These findings suggest that ELN-TGFBR2 engagement promotes autocrine TGF-β1 secretion by immune cells, thereby compromising their cytotoxic and antitumor functions. Accordingly, staining for cleaved-caspase 3 was reduced, while the proliferation marker PCNA was increased in the ELN treated group, indicating suppression of tumor cell apoptosis and enhanced tumor growth under high ELN conditions in the TME (Figure 6, E and F).

Given the extensive transcriptional reprogramming induced by ELN within the TME, we next performed mRNA profiling in the 72-hour coculture system. Principal component analysis (PCA) demonstrated distinct transcriptional clustering of TIOs under different treatment conditions (Figure 6G). Gene expression profiling revealed enrichment of pathways related to EMT, TGF-β1 signaling, T cell exhaustion, and macrophage differentiation, indicating a global remodeling of BCa-immune interactions driven by ELN. Conversely, cotreatment with the TGFBR2 inhibitor ITD1 largely reversed these transcriptional alterations, restoring the expression landscape toward control levels. Consistently, heatmap and GSVA confirmed that ELN markedly increased T cell exhaustion and M2 macrophage signatures, along with upregulation of *Tgfb1*-associated and EMT-related genes and downregulation of apoptosis-related transcripts, whereas ITD1 cotreatment abrogated these effects in ELN-treated TIOs (Figure 6H and Supplemental Figure 17D).

Collectively, these data demonstrate that ELN exerts multifaceted effects on the tumor–immune ecosystem by engaging the TGFBR2/TGF-β1 axis, thereby reprogramming immune cell states, enhancing BCa organoid metastasis, and promoting immune evasion in BCa.

### In vivo activation of the ELN/TGFBR2/TGF-β1 axis accelerates BCa progression and impairs immunotherapeutic response.

**Figure 7:**
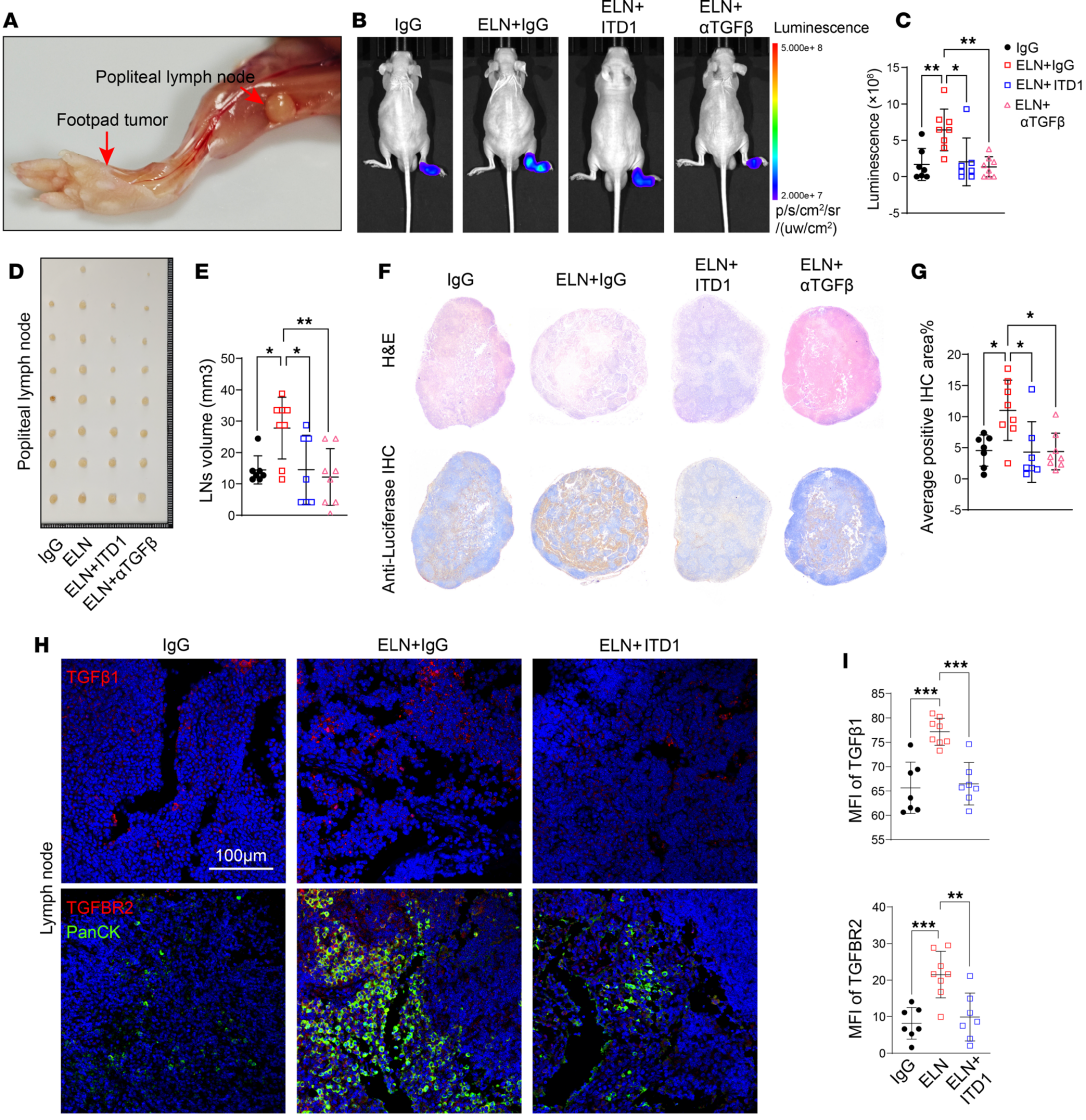
ELN/TGFBR2 axis accelerates BCa lymph node metastasis. (**A**) Schematic of the footpad metastasis model illustrating the primary footpad tumor and draining popliteal LNs. (**B** and **C**) Bioluminescence imaging (**B**) and quantification of luminescence intensity (**C**) in popliteal LNs from mice treated with IgG, rm-ELN + IgG, rm-ELN + ITD1, or rm-ELN + αTGF-β. *n* = 7–8 for each group. *P* values were determined by 1-way ANOVA followed by Tukey’s multiple-comparison test. (**D** and **E**) Representative images of isolated popliteal LNs (**D**) and quantification of metastatic LN volumes (**E**) across treatment groups. *P* values were determined by 1-way ANOVA followed by Tukey’s multiple-comparison test. (**F** and **G**) H&E staining and anti-luciferase IHC (**F**) of metastatic LNs, with quantification of positive IHC area percentage (**G**). *P* values were determined by 1-way ANOVA followed by Tukey’s multiple-comparison test. (**H** and **I**) Representative immunofluorescence images (**H**) of TGF-β1 (red, top) and TGFBR2 (red, bottom) in PanCK^+^ (green) metastatic lymph nodes, with corresponding quantification of MFI (**I**). Scale bar: 100 μm. *P* values were determined by 1-way ANOVA followed by Tukey’s multiple-comparison test. Data are shown as mean ± SEM. *P < 0.05, **P < 0.01, ***P < 0.001.

To assess the in vivo relevance of the ELN/TGFBR2/TGF-β1 axis, we established a footpad metastasis model of BCa in mice (Figure 7A). Administration of rm-ELN markedly increased popliteal lymph node metastasis, as indicated by enhanced bioluminescence signals and larger nodal tumor volumes compared with controls (Figure 7, B–E). Histological analyses confirmed greater tumor infiltration within metastatic nodes, which was effectively reduced by ITD1 or TGF-β neutralizing antibody treatment (Figure 7, F and G). Immunofluorescence further revealed that ELN exposure induced strong upregulation of TGF-β1 and its receptor TGFBR2 within the lymph node metastases, both of which were significantly suppressed upon ITD1 cotreatment (Figure 7, H and I).

**Figure 8:**
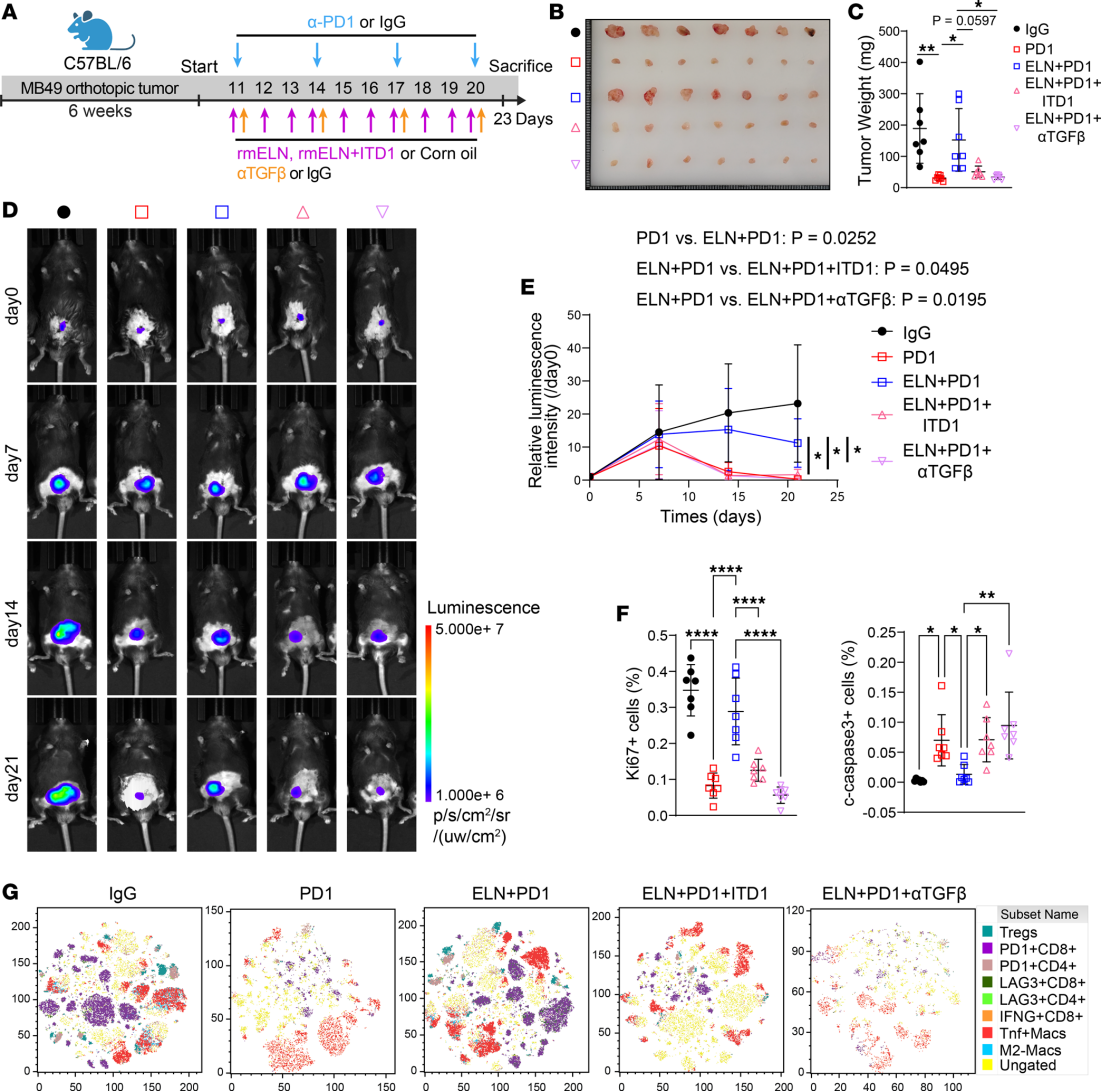
Targeting the ELN/TGFBR2 axis improves the immunotherapeutic response in vivo. (**A**) Schematic illustrating the MB49 orthotopic BCa model and the treatment schedule. Mice were treated with anti–PD-1 or IgG, combined with rm-ELN, ITD1, or αTGF-β as indicated. (**B** and **C**) Representative images of harvested orthotopic tumors (**B**) and endpoint tumor weights (**C**) in each treatment group. *n* = 7 for each group. *P* values were determined by 1-way ANOVA followed by Tukey’s multiple-comparison test. (**D** and **E**) Longitudinal bioluminescence imaging (**D**) and quantification of relative luminescence intensity (**E**) over 21 days. *P* values were determined by 2-way ANOVA with Tukey’s post hoc test. (**F**) Quantification of Ki67^+^ proliferating cells and cleaved-caspase 3^+^ apoptotic cells within orthotopic tumors across treatment groups. *P* values were determined by 1-way ANOVA followed by Tukey’s multiple-comparison test. (**G**) t-SNE visualization of spectral flow cytometry data displaying tumor-infiltrating immune cell subsets, including Tregs, exhausted T cells (PD-1^+^, LAG3^+^), and M2-like macrophages under the indicated treatments. Data are shown as mean ± SEM. *P < 0.05, **P < 0.01, ****P < 0.0001.

We next evaluated the effect of ELN on anti–PD-1 immunotherapy using an MB49 orthotopic BCa model (Figure 8A). Compared with anti–PD-1 monotherapy, the combination of ELN with anti–PD-1 resulted in accelerated tumor outgrowth, as evidenced by significantly increased bioluminescence intensity, higher endpoint tumor weights, and an expanded proportion of Ki67^+^ proliferating cells. These protumorigenic effects were markedly attenuated by pharmacologic inhibition of TGFBR2 via ITD1 or by TGF-β neutralization, which also induced robust cleaved-caspase 3^+^ apoptotic signals within the tumor parenchyma (Figure 8, B–F, and Supplemental Figure 18A). Spectral flow cytometry further demonstrated that ELN treatment enriched PD-1^+^ and LAG3^+^ Tex, Tregs, and CD206^+^ M2-like macrophages, while concomitantly reducing TNF^+^ proinflammatory macrophages (Figure 8G and Supplemental Figure 18B). This profound induction of a therapy-resistant immune microenvironment was largely abolished by TGF-β/TGFBR2 blockade, establishing that ELN impairs anti–PD-1 efficacy through activation of the TGF-β1 signaling axis.

Collectively, these in vivo data establish the ELN/TGFBR2/TGF-β1 axis as a potent promoter of metastasis and immunotherapy resistance in BCa.

## Discussion

Metastasis and immunosuppression are 2 major determinants of lethality in advanced BCa (24, 25). Through integrated analyses of multiple clinically relevant datasets, we identified ELN—a common but often overlooked protein secreted into the ECM—as a critical regulator of these processes, showing stronger associations with patient survival and immunotherapy response than other ECM-related factors in BCa. Recent evidence links ELN to EMT in colorectal cancer (23), but its mechanism and immunological effects are unclear. Here, we observed that *ELN* expression is markedly elevated in CAFs within metastatic BCa and in nonresponders to anti–PD-1 therapy, suggesting that CAF-secreted ELN may contribute to both tumor dissemination and immune evasion. Collectively, these findings identify ELN^+^ CAF as a key determinant of BCa aggressiveness and a potential target for clinical intervention.

CAFs promote BCa progression through multiple strategies, among which the secretion of ECM proteins to remodel the TME represents a central mechanism (26, 27). Our results demonstrate that ELN^+^ CAFs enhance the metastatic capacity of BCa cells and increase the infiltration of CD4^+^ Tex, CD8^+^ Tex, Tregs, and M2 like macrophages. ELN^+^ CAFs are significantly enriched during BCa metastasis, where they actively remodel the TME and foster immune resistance. These findings are consistent with a previous report, supporting the notion that ELN^+^ CAFs act as pivotal mediators of BCa progression.

Although *ELN* has long been considered a highly stable gene with minimal expression after adulthood, it marked reexpression during tissue fibrosis or tumor progression indicates that the altered microenvironment actively induces tropoelastin secretion (28, 29). Interestingly, *ELN* is viewed as a downstream effector of TGF-β1, being upregulated in fibrotic tissues under sustained TGF-β1 signaling; for instance, TGF-β1 treatment of fibroblasts has been observed to increase *ELN* expression (30, 31). In contrast, our results reveal an unexpected upstream function of secreted tropoelastin; it enhances TGF-β1 expression and signaling within the BCa TME. Notably, TGF-β1 acts as a multifunctional cytokine in promoting metastasis and immunosuppression in advanced cancers (12, 32, 33). Activated TGF-β1 acts through canonical *SMAD* and noncanonical pathways to induce the EMT program in epithelial cells and M2 macrophage polarization, as well as T cell exhaustion, thereby reinforcing an immunosuppressive and prometastatic TME. This finding suggests that the remodeled TME upregulates ELN secretion, which in turn amplifies TGF-β1 activity, establishing a feed forward loop that reinforces tumor progression and immune resistance. Briefly, ELN functions as a critical stromal regulator that remodels the TME and concurrently induces TGF-β1 expression within the TME; together, they synergistically accelerate BCa lethality.

*TGFBR2* represents a pivotal receptor mediating canonical and noncanonical TGF-β1 signaling pathway. Given its broad expression across multiple cell types within the TME, including tumor cells, T cells, and macrophages, all of which are capable of secreting TGF-β1, our findings suggest that ELN acts as a binding partner of TGFBR2 and facilitates autocrine TGF-β1 signaling in these cells. Single-cell and spatial transcriptomic analyses further revealed that as tumor aggressiveness increases, ELN secretion from CAFs is markedly amplified, leading to enhanced interactions between stromalderived ELN and TGFBR2 on tumor and immune cells. This coordinated communication establishes a prometastatic and immunosuppressive TME characterized by the coexistence of invasive tumor cells and immunesuppressive populations. Consistently, both in vitro and in vivo experiments demonstrated that pharmacologic inhibition of TGFBR2 attenuated the ELN-induced upregulation of TGF-β1 in tumor and immune cells, whereas neutralization of downstream TGF-β1 disrupted the ELN-driven positive feedback loop.

In summary, these results identify tumor and immune cell TGFBR2 as key target molecules of ELN within the TME and highlight a critical ELN/TGFBR2/TGF-β1 signaling axis that reprograms the microenvironment toward a protumorigenic and immunerefractory state. Given the prominent role of the ELN/TGFBR2/TGF-β1 axis in orchestrating immunosuppression and metastasis, targeting this pathway either as a monotherapy or in combination with immune checkpoint blockade represents a promising therapeutic strategy for advanced BCa.

This study has certain limitations. First, our research focused on full-length ELN; however, the degradation fragments known as elastin-derived peptides (EDPs), which are typically generated by elastase-mediated cleavage, were not investigated in vivo. The potential biological functions of these fragments remain to be clarified. Second, we primarily examined the effects of ELN on T cells and macrophages; the responses of other immune populations within the TME, such as DCs and neutrophils, were not explored in this study. Overall, future studies addressing these questions will help refine our understanding of ELN-mediated regulation of the TME and its implications for therapeutic targeting.

## Methods

The antibodies and primers used in this study are included in Supplemental Tables 3 and 5. The detailed methods are given in Supplemental Methods.

### Sex as a biological variable.

Human studies included both male and female patients. For animal studies, all tumor growth and treatment experiments were conducted in male mice. Sex was not explicitly considered as a variable in analyses.

### Statistics.

All statistical analyses and data visualizations were performed using R software (version 4.4.1) or GraphPad Prism 10.1.2. Kaplan-Meier survival curves were generated to evaluate the prognostic value of variables based on median expression cutoffs. Correlations between 2 variables were assessed using Spearman’s correlation analysis. Comparisons between 2 groups were conducted using either a 2-tailed unpaired/paired *t* test or a 2-tailed Mann-Whitney *U* test. Comparisons involving more than 2 groups were performed using 1-way ANOVA with Tukey’s multiple-comparison test. Tumor growth curves were compared using 2-way ANOVA with Tukey’s multiple-comparison test. For differential expression analyses, *P* values were adjusted for multiple testing using the Benjamini-Hochberg FDR correction. A *P* value or an adjusted *P* < 0.05 was considered statistically significant.

### Study approval.

All experimental protocols and animal handling procedures were conducted in strict accordance with institutional guidelines and were approved by the IACUC of Anhui Medical University (no. 20252098). Ethical review for the use of pathological specimens and access to patient records was obtained from the IRB of the First Affiliated Hospital of Anhui Medical University (PJ 2025-03-43). Written informed consent was provided by all participants and/or their legal representatives as required.

### Data availability.

The raw sequence data presented in this paper have been deposited in the NCBI Sequence Read Archive (PRJNA1245412 and PRJNA1369659). Bulk RNA-seq data has been deposited into the Zenodo database (DOI: 10.5281/zenodo.15120987). Any additional code or information necessary to reanalyze the data in this paper can be obtained upon request from the lead contact. Values for all data points in graphs are reported in the Supporting Data Values file.

## Author contributions

LZ, YD, and HW designed this study. WX, SW, JG, JH, CA, CC, and CJ performed the experiments and analyzed and interpreted results. WX, NL, and SZ analyzed bioinformatics data. CJ and CA performed SPR and MST assays. HX, YX, and JZ provided clinical samples from patients with BCa. ZW, ZD, HD, and XL provided scientific inputs for this study and contributed to planning experimental models. QF and YL performed pathological screening of the BCa sections. LZ and HW are the principal investigators and the guarantors of the study and are responsible for this study concept, design, data analysis, and interpretation and procuring funds. WX wrote the manuscript. All the authors read the manuscript and approved its submission. For co–first authors, the authorship order was assigned based on the relative scope of their experimental contributions and their roles in manuscript preparation.

## Conflict of interest

The authors have declared that no conflict of interest exists.

## Funding support

This work was supported by the following organization:

- National Natural Science Foundation of China (U21A20375).

## Supplementary Material

Supplemental data

Unedited blot and gel images

Supplemental tables 1-7

Supporting data values

## Version 1 05/22/2026

Electronic publication

## References

[1] Grayson M (2017). Bladder cancer. Nature.

[2] Kamat AM (2017). BCG-unresponsive non-muscle-invasive bladder cancer: recommendations from the IBCG. Nat Rev Urol.

[3] Patel VG (2020). Treatment of muscle-invasive and advanced bladder cancer in 2020. CA Cancer J Clin.

[4] Gong L (2025). Cancer immunology data engine reveals secreted AOAH as a potential immunotherapy. Cell.

[5] Uhlen M (2015). Proteomics. Tissue-based map of the human proteome. Science.

[6] Wei W (2021). Cell type-selective secretome profiling in vivo. Nat Chem Biol.

[7] Huang Y (2025). Secreted proteins in treating metabolic dysfunction-associated steatotic liver disease: from bench towards bedside. Protein Cell.

[8] Ferrara N (2004). Discovery and development of bevacizumab, an anti-VEGF antibody for treating cancer. Nat Rev Drug Discov.

[9] Szarvas T (2011). Matrix metalloproteinases and their clinical relevance in urinary bladder cancer. Nat Rev Urol.

[10] Child F (2025). The hypoxic ECM and neutrophils in MIBC immunotherapy resistance. Nat Rev Urol.

[11] Fane M, Weeraratna AT (2020). How the ageing microenvironment influences tumour progression. Nat Rev Cancer.

[12] Massague J, Sheppard D (2023). TGF-β signaling in health and disease. Cell.

[13] Wong JKM (2024). TGF-β signalling limits effector function capacity of NK cell anti-tumour immunity in human bladder cancer. EBioMedicine.

[14] Sutherland TE (2023). The extracellular matrix and the immune system: a mutually dependent relationship. Science.

[15] Yao Z (2023). Proteogenomics of different urothelial bladder cancer stages reveals distinct molecular features for papillary cancer and carcinoma in situ. Nat Commun.

[16] Mariathasan S (2018). TGFβ attenuates tumour response to PD-L1 blockade by contributing to exclusion of T cells. Nature.

[17] Chen Z (2020). Single-cell RNA-seq highlights the role of inflammatory cancer-associated fibroblasts in bladder urothelial carcinoma. Nat Commun.

[18] Cui C (2024). Inhibition of JNK signaling overcomes cancer-associated fibroblast-mediated immunosuppression and enhances the efficacy of immunotherapy in bladder cancer. Cancer Res.

[19] Kanemaru K (2023). Spatially resolved multiomics of human cardiac niches. Nature.

[20] Cui A (2024). Dictionary of immune responses to cytokines at single-cell resolution. Nature.

[21] Yeh HW (2018). PSPC1 mediates TGF-β1 autocrine signalling and Smad2/3 target switching to promote EMT, stemness and metastasis. Nat Cell Biol.

[22] Li Y (2021). OSR1 phosphorylates the Smad2/3 linker region and induces TGF-β1 autocrine to promote EMT and metastasis in breast cancer. Oncogene.

[23] Li J (2020). Elastin is a key factor of tumor development in colorectal cancer. BMC Cancer.

[24] Tran L (2021). Advances in bladder cancer biology and therapy. Nat Rev Cancer.

[25] Lopez-Beltran A (2024). Advances in diagnosis and treatment of bladder cancer. BMJ.

[26] Flynn JM (2025). Plasticity and Functional Heterogeneity of Cancer-Associated Fibroblasts. Cancer Res.

[27] Lee CJ (2024). The dysadherin/MMP9 axis modifies the extracellular matrix to accelerate colorectal cancer progression. Nat Commun.

[28] Heinz A (2020). Elastases and elastokines: elastin degradation and its significance in health and disease. Crit Rev Biochem Mol Biol.

[29] Hu S (2022). TDO2^+^ myofibroblasts mediate immune suppression in malignant transformation of squamous cell carcinoma. J Clin Invest.

[30] Hume RD (2023). Tropoelastin improves post-infarct cardiac function. Circ Res.

[31] Zi Y (2025). Human umbilical cord mesenchymal stem cell exosomes promote elastin production and acute skin wound healing via TGFβ1-Smad pathway. Mol Cell Biochem.

[32] Lee JH (2024). TGF-β and RAS jointly unmask primed enhancers to drive metastasis. Cell.

[33] Deng Z (2024). TGF-β signaling in health, disease, and therapeutics. Signal Transduct Target Ther.

