# Supplementary material for: ELN orchestrates prometastatic and immunosuppressive niche in bladder cancer via TGFB1 autocrine signaling
Source: JCI Insight. 2026 May 22;11(10):e194700. doi: 10.1172/jci.insight.194700 (PMC13232729; doi:10.1172/jci.insight.194700)
Supplement: Supplemental data [file jciinsight-11-194700-s247.pdf]

Supplementary Methods

Human BCa Samples. The clinical samples in this study included patients with BCa who underwent radical cystectomy at the First Affiliated Hospital of Anhui Medical University, as well as formalin-fixed paraffin-embedded (FFPE) samples from the Department of Pathology (Supplemental Table 2). This study adhered to the principles of the Helsinki Declaration and was approved by the Medical Ethics Committee of the First Affiliated Hospital of Anhui Medical University.

Animals. Male wild-type C57BL/6J mice (6–10 weeks old) and BALB/c nude mice (4 weeks old) were obtained from GemPharmatech (Nanjing, China). Mice were maintained under specific pathogen-free conditions with a standard 12-hour light/12-hour dark cycle. All animal procedures were conducted in accordance with the ethical guidelines outlined for the care and use of laboratory animals. All procedures were approved by the Institutional Animal Care and Use Committee (IACUC) of Anhui Medical University.

Antibodys and proteins. The antibodies and proteins used in this study are listed in Supplemental Table 3 and 4. All purified proteins were biologically active and diluted according to the instructions and stored at -80°C for subsequent use. For cell culture experiments, rm-ELN was used at a final concentration of 1 μg/mL.

Cell lines culture. Human BCa cell line T24 and mouse BCa cell line MB49 were obtained from the ATCC (American Type Culture Collection). Mycoplasma contamination was excluded using PCR-based methods, and the identity of all cell lines was confirmed by short tandem repeat (STR) analysis. These cells were cultured in DMEM supplemented with 10% FBS and 1% penicillin–streptomycin (P/S) at 37°C in a 5% CO_2 atmosphere.

PBMCs isolation and culture. Peripheral blood was collected using EDTA-coated vacuum blood collection tubes, ensuring that no hemolysis occurred. Next, 10 mL of blood was carefully transferred into a sterile 50 mL centrifuge tube. A 1:1 mixture of 10 mL Ficoll-Paqueand 10 mL sterile PBS was gently layered beneath the blood sample to avoid intermixing. The sample was centrifuged at 800 g for 35 minutes with low acceleration and deceleration to ensure proper separation. After centrifugation, the PBMC layer (a white, cloudy band located at the plasma-Ficoll interface) was carefully aspirated using a pipette and transferred into a new sterile 50 mL centrifuge tube. The cells were washed by resuspension in PBS up to 50 mL and centrifugation at 600 g for 10 minutes. Red blood cells were lysed with the addition of 3 mL of red blood cell lysis buffer, incubated for 3 minutes, and subsequently washed with PBS up to 15 mL. The washing process was repeated 2-3 times to ensure the removal of residual debris and proteins. The anti-human CD3 antibody (eBioscience, 16-

0037-85) was diluted to 5 μg/mL in sterile PBS and used to coat six-well culture plates at 37°C for 2 hours. The remaining coating solution was discarded, and the wells were washed three times with PBS. The freshly isolated PBMCs were seeded into the coated wells at a density of 1 × 10^6 cells/mL, and 2 mL of complete culture medium (RPMI-1640 supplemented with 10% FBS, 1% P/S, 5 μg/mL anti-human CD28 (eBioscience, 16-0289-85), 50U/mL IL2 (Yeasen, 90103ES60) and 50 ng/mL M-CSF (PHC9504, Invivogen)) were added to each well. The cells were cultured at 37°C in a humidified 5% CO_2 incubator. Antigenic stimuli were added subsequently according to experimental requirements.

T cell purification and macrophage differentiation. Purified T cells were obtained from freshly isolated PBMCs using the Miltenyi Human $CD3^+$ T Cell Isolation Kit (Miltenyi Biotec, Germany) according to the manufacturer's instructions. The purity of the isolated T cells was confirmed by immunofluorescence staining using an anti-CD3 antibody staining. For macrophage preparation, the density of freshly isolated PBMCs was adjusted to 1 × 10^6 cells/mL with RPMI-1640 medium. The cell suspension was added into a tissue culture flask and incubated at 37 °C in a 5% CO_2 incubator for 2 hours to allow monocyte adherence. Non-adherent cells were carefully removed, and the attached cells were washed gently with pre-warmed PBS to eliminate residual lymphocytes. After washing, fresh RPMI-1640 medium supplemented with 10% FBS was

added, and the cultures were maintained at 37 °C and 5% CO_2 for subsequent differentiation into macrophages.

Primary CAFs isolation and culture. Primary CAFs were isolated from freshly resected human BCa obtained during surgery. Tumor tissues were washed extensively in cold PBS and mechanically minced into approximately 1–2 mm^3 fragments. The preparation of single-cell suspensions followed the procedure described in the “Single-cell suspension preparation” section. The resulting suspensions were incubated with antibodies for CAFs enrichment and subsequently subjected to fluorescence activated cell sorting (FACS). The purity of isolated CAFs was verified by immunofluorescence staining with an anti-vimentin antibody. CAFs were maintained in fibroblast medium supplemented with 2% FBS, 1% P/S, and 1% fibroblast growth supplement (Sciencell, catalog no. 2301) at 37 °C in a humidified incubator with 5% CO_2.

Lentivirus and short interfering RNAs transfer. Lentiviruses containing luciferase were purchased from HANBIO Technology Co. Ltd., Shanghai. Cells were transduced with lentiviral in the presence of 5 μg/ml polybrene. After 24 h, culture medium was removed, and fresh medium was added. Three days after transduction, 10 μg/ml Blasticidin S was added to the medium. After 1 week of antibiotic selection, T24-Luc and MB49-Luc cells were obtained. SiRNA

targeting *ELN* (si*ELN*) and non-targeting siRNA negative controls (siNC) were purchased from HANBIO Technology Co. Ltd., Shanghai (Supplemental Table 5). SiRNA transfections were performed using RNAiMAX (Invitrogen). The efficiency of silencing was assessed by immunofluorescence and immunoblotting 48 hours post-transfection.

Co-culture experiments. Co-culture experiments were performed using Transwell inserts (0.4 or 8μm pore size, Corning) to assess paracrine interactions between different cell types. For tumor–CAFs co-cultures, T24 (5×10^4) cells were seeded in the upper chamber, while CAFs (1.5×10^5) were plated in the lower chamber. The cells were cultured in complete medium for 72 hours. For immune cell-CAFs co-cultures, CAFs (1×10^5) were plated in the upper chamber, and $CD3^+$ T cells (2×10^5) or macrophages (2×10^5) were added to the lower chamber containing RPMI-1640 complete medium. After 48 hours, cells from the lower compartment were harvested for subsequent analysis.

Flow cytometry. Cells were collected and processed into single-cell suspensions and then subjected to surface marker staining by first being incubated with anti-human CD16/CD32 antibody (BioLegend, Cat# 422301), followed by incubation with the specified antibodies on ice for 30 min, and then washed with staining buffer. A fixable viability dye (L34962, Thermo Scientific)

was employed to discriminate between live and dead cells. These stained cells were analyzed using NovoCyte Penteon flow cytometer (Agilent, CA, USA) and Cytek Aurora flow cytometer (Cytek, CA, USA), and data were processed using FlowJo.

Transwell assay. T24 cells (5×10^4) were seeded into the upper chamber and cultured in FBS-free medium for 24 hours. Subsequently, 700 μL complete medium supplemented with 10% FBS was added into the lower chamber of the transwell insert to promote cell migration. After another 24 hours incubation for cell migration, the upper chambers were taken out, and cells that had migrated through the membrane were fixed with 4% paraformaldehyde for15min and stained with 0.1% crystal violet for 15 min. Invasion assays were performed using the transwell chambers in the growth factor–reduced Matrigel (Corning, New York, USA).

Western blotting. Total cellular protein was extracted using RIPA lysis buffer and then quantified using the Bradford method (Sigma-Aldrich). The protein lysates were separated by SDS–PAGE, electro-blotted onto polyvinylidene difluoride membranes (Millipore; Sigma-Aldrich), probed with the indicated primary antibody followed by the HRP-conjugated secondary antibody, and then detected by enhanced chemiluminescence with LI-COR Imaging Systems (Lincoln, Nebraska, USA).

ELISA. TGFβ1 levels in the cell supernatant were quantified using enzyme-linked immunosorbent assay (ELISA) kits specific for TGFβ1, according to the manufacturer's instructions (Proteintech, Wuhan, China).

Immunofluorescence. Cells were plated onto glass coverslips, fixed with 4% paraformaldehyde, permeabilized with 0.1% Triton X-100, and then stained with the indicated primary antibody at 4°C, followed by the corresponding secondary antibody. The antibody-labeled cells on coverslips were counterstained with DAPI and subsequently mounted using a mounting solution. Primary antibodies listed in Supplemental Table 3 were used to stain tissue sections. Fluorescently labeled secondary antibodies were utilized to visualize the primary antibodies. Images were acquired using a confocal microscope (SUNNY CSIM 130, Suzhou, China).

Surface plasmon resonance. SPR experiments were conducted using a Biacore T200 (GE Healthcare; Chicago, Illinois, USA). Following the manufacturer's instructions, recombinant mouse or human ELN protein was immobilized onto the CM5 sensor surface using a standard amine coupling procedure. HBS-N buffer (10 mM HEPES, pH 7.4, and 150 mM NaCl) supplemented with 0.05% Tween-20 was employed as the running buffer for immobilization. Affinity measurements were performed at 25°C in a buffer

containing 25 mM HEPES (pH 7.4), 150 mM NaCl, 0.1% Tween 20, and 2% DMSO. The ELN protein concentration was prepared via a twofold dilution series from 2000 nM to 125 nM, and the protein was flowed through the chip channel at varying concentrations, both with and without ELN protein, at a flow rate of 30 μL/min for 180 seconds. After each concentration point, the chip was regenerated using NaOH at a flow rate of 30 μL/min for 60 seconds, followed by real-time recording and data storage. To eliminate non-specific binding and solvent effects, solvent correction was applied using slight variations in DMSO concentration between samples. Finally, the binding rates (ka), dissociation rates (kd), and the equilibrium dissociation constant (KD = kd/ka) were analyzed and categorized using the Biacore T200 analysis software (GE Healthcare), with each experiment being performed in triplicate.

Microscale thermophoresis. MST experiments were analyzed using a Monolith NT.115 instrument (NanoTemper Technologies). His-tagged TGFBR2 was fluorescently labeled with the RED-tris-NTA Protein Labeling Kit (NanoTemper, MO-L018) and measured in standard capillaries (MO-K022). A 1× PBST buffer was prepared by diluting 5× PBST with 8.0 mL of double-distilled water. The fluorescent dye solution (2.5 μM) was dissolved in 50 μL PBST and diluted to a final concentration of 100 nM. For labeling, 100 μL of 400 nM His-TGFBR2 was mixed with 100 μL dye solution and 10 μL BSA, followed by incubation at room temperature in the dark for 30 minutes. The mixture was then centrifuged at

12,000 rpm for 10 minutes at 4 °C to remove unbound dye. Ligand titration was performed by preparing a two-fold serial dilution of rm-ELN in PBST. In a series of PCR tubes, tubes 2–16 received 10 μL PBST, while tube 1 contained 20 μL of rm-ELN. Sequential 10 μL transfers were carried out between tubes with thorough mixing, discarding 10 μL from the final tube to maintain equal volumes. Subsequently, 10 μL of labeled His-TGFBR2 was added to each tube, gently mixed, and samples were loaded into MST capillaries for measurement.

Mouse BCa organoids. Bladder cancer organoids were generously provided by Professor Yang Li in Anhui medical university. An in situ mouse model of bladder cancer was established using BBN (Santa Cruz, sc-486264) dissolved in drinking water at a concentration of 0.05% (w/v), which was administered to male mice for a duration of 30 weeks, as previously described (1). In this study, organoid cultures were established using a carefully formulated medium comprising 100 ml of advanced DMEM/F12 (Gibco 12634010), supplemented with 1 ml of GlutaMAX (Gibco 35050061), 1 ml of HEPES (final concentration 10 mM; Gibco 15630130), and 200 μl each of the antibiotics Primocin and Normocin (Invivogen ant-pm-2/ant-nr-2) to prevent bacterial contamination. Additionally, the medium contained 1 ml of B27 supplement without vitamin A (Gibco 12587010) to provide essential growth factors, along with 200 μl of epidermal growth factor (EGF) at a final concentration of 50 ng/ml (Thermo Scientific 315-09-500UG) to promote cellular proliferation. The medium was

further enriched with 200 µl of Y27632·2HCl at a final concentration of 10 µM (MCE HY-10583), 200 µl of N-acetyl-cysteine at a final concentration of 1 mM (Sigma-Aldrich A9165), and 200 µl of nicotinamide at a final concentration of 10 mM (Sigma-Aldrich N0636). To investigate the effects of TGFβ, A8301 (TGFβ inhibitor) was included in the medium at a final concentration of 1 µM (Sigma-Aldrich SML0788), except during routine culture, where the media was replaced 24-48 hours prior to experimentation to exclude this inhibitor.

Organoids were adapted through two passages over a total of seven days, with media changes conducted three times weekly (500 µl per well) to enhance organoid growth and viability. During passage, digestion involved adding 2 ml of cold Cell Recovery Solution (Corning 354253) into a 24-well plate, followed by mechanical disruption using a pipette to break down the Matrigel (Corning 356231) matrix. The resultant mixture was then carefully transferred to a 15 ml conical tube and incubated on ice for 40 minutes. After this incubation, the samples were centrifuged at 350 g for 5 minutes at 4°C, allowing for the separation into three distinct layers: the supernatant containing the Cell Recovery Solution, a middle layer with smaller organoids, and a pellet of larger organoids at the bottom. Following centrifugation, the supernatant was discarded, and excess smaller organoid and Matrigel mixture was also removed to isolate the larger organoids. The resulting organoid pellet could then be re-suspended in fresh Matrigel for further culture or experimental applications.

For co-culture experiments involving primary macrophages and MLN cells, the prepared organoids underwent a specific digestion process to facilitate optimal integration. In addition, primary macrophages were isolated from the bone marrow of C57BL/6J mice and cultured in RPMI1640 supplemented with macrophage colony-stimulating factor (M-CSF, 50 ng/mL, Abcam, M9170). Mature macrophages were obtained on day 7 of culture. Concurrently, MLN cells, which were primarily composed of T cells, were cultured in an RPMI 1640-based medium supplemented with essential nutrients. These preparatory steps provided a solid foundation for the comprehensive investigation of immune interactions within the organoid systems.

Mouse model. For the Popliteal lymphatic metastasis model, T24-Luc cells (5×10^6) were inoculated into the footpads of the mice. Then, the mice were randomly divided into 4 groups and injected with IgG, rm-ELN+IgG, rm-ELN+IgG+ITD1 or rm-ELN+αTGFβ. The popliteal LNs were excised when the footpad tumors were 200 mm^3. For the MB49 orthotopic BCa model, 6 weeks old C57BL/6 mice were anesthetized with isoflurane inhalation, and the lower abdominal skin and peritoneal wall were incised to create a ~5 mm exposure of the bladder. After emptying the bladder of residual urine, 25 μL of MB49-Luc cell suspension (5×10^5 cells) was slowly injected into the bladder wall using a microsyringe. Following injection, both the muscular and skin layers were closed with absorbable sutures. Eleven days after tumor implantation, mice

were randomly assigned to five experimental groups receiving intraperitoneal injections of IgG, anti-PD-1, rm-ELN+anti-PD-1, rm-ELN+ITD1+anti-PD-1, or rm-ELN+αTGFβ+anti-PD-1. Unless specified, 10 μg rm-ELN and 1mg/kg ITD1 was used for each intratumoral or intraperitoneal injection daily. For antibody, 200 μg anti-mouse PD-1 (BE0273, BioXcell) or 250 μg anti-TGFβ (BE0057, BioXcell) per mouse was injected intraperitoneally every 3 days. In vivo bioluminescence imaging of luciferase activity was analyzed by IVIS Spectrum in Vivo Imaging System (Aniview, GuanZhou, China).

RNA sequencing and analysis. Bulk RNA sequencing of tumor-infiltrating organoids (TIOs) was performed by Gene Denovo Biotechnology Co., Ltd. (Guangzhou, China). RNA libraries were constructed following the manufacturer's standard protocol and sequenced on the Illumina NovaSeq 6000 platform. The resulting raw reads were processed to obtain count and transcripts per million (TPM) matrices, which were subsequently used for downstream gene-expression analyses.

Single-cell suspension preparation. Fresh bladder tumor specimens were processed immediately after surgical resection (Supplemental Table 6). Tissues were washed thoroughly with cold PBS to remove blood and debris, then minced into small fragments (< 1 mm^3) and transferred into 5 mL of DMEM containing collagenase IV (1 mg/mL; Thermo). The samples were incubated at

37 °C for 60 minutes in a shaking incubator to facilitate enzymatic digestion. The resulting cell suspension was diluted with 10 mL PBS, filtered through a 70 μm cell strainer, and centrifuged at 800 rpm for 5 minutes. The cell pellet was collected and resuspended in cell preservation solution for subsequent analysis.

Single-cell transcriptomic sequencing. For human BCa samples, single-cell RNA-seq libraries were generated using the DNBelab C Series High-throughput Single-Cell RNA Library Prep Set (MGI, #940-000047-00) following the manufacturer's protocol. Sequencing was performed on the DNBSEQ-T7 platform to obtain high-throughput transcriptomic data. Raw reads were processed with DNBC4Tools for quality control, demultiplexing, and alignment to the human reference genome, yielding gene-barcode count matrices for downstream analysis. In addition, human PBMCs were encapsulated into oil droplets using the MobiNova-100 platform (MobiDrop, Zhejiang, China). The resulting FASTQ files were processed with the MobiVision software pipeline to generate gene-expression matrices through demultiplexing of cellular barcodes, mapping reads to both the genome and transcriptome using the STAR aligner, and down-sampling as needed to achieve normalized aggregate data across samples. Public single-cell RNA-seq datasets (PRJNA662018) were incorporated to characterize the cellular composition of the TME. Raw sequencing data were processed using Cell Ranger (v6.1.2, 10x Genomics) to generate normalized aggregate

gene-expression matrices from the samples included in PRJNA662018. Subsequent data processing was conducted in R or python. Unique molecular identifier (UMI) count matrices were filtered to remove low-quality droplets and potential doublets using UMI thresholds combined with the Scrublet package. Cells with fewer than 1,000 UMIs or greater than 10% mitochondrial gene content were excluded as low-quality. Furthermore, cells detecting fewer than 300 or more than 6,000 genes were filtered out to ensure data consistency and reliability.

Subsequent analyses of scRNA-seq data, including normalization, log transformation, identification of top variable genes, and canonical correlation analysis (harmony) for batch effect correction, were conducted with "Seurat" and "Scanpy". To characterize the distribution of cell types, odds ratios were calculated based on established reports to indicate preferences. To identify malignances, copy number variation levels were assessed in each cell using the inferCNVpy package. To evaluate hallmark scores for each cell, we calculated a gene set activity score by applying the AddModuleScore function. For analyzing the expression dynamics of cellular subpopulations, we employed velocyto (v0.17.17) to estimate RNA velocity of individual cells by distinguishing between spliced and unspliced mRNAs. To map differentiation within the TME, pseudotime analysis was carried out using Monocle2 to elucidate differentiation relationships among cell types. To assess transcription factor activity within each cell, pySCENIC analysis was conducted across all

single cells. The CellPhoneDB software was applied to investigate the interaction network among cell types.

Transcriptome data and clinical information. Transcriptome profile analysis data and clinical annotations for 32 types of malignancies (TGCT, LGG, ACC, CESC, PCPG, THCA, KICH, PRAD, BRCA, DLBC, OV, THYM, HNSC, KIRC, GBM, LIHC, KIRP, SARC, ESCA, UVM, MESO, PAAD, READ, SKCM, UCEC, LUAD, CHOL, COAD, STAD, BLCA, LUSC, and UCS) were obtained from The Cancer Genome Atlas (TCGA; https://portal.gdc.cancer.gov/). For validation purposes, we utilized multiple high-throughput sequencing datasets: GSE133624, GSE136401, GSE154261, GSE176307, GSE188715, GSE224248, GSE236932, GSE245748, GSE293398, GSE294703, GSE294705, GSE295809 and EMTAB4321. Additionally, we employed the R package "IMvigor 210 CoreBiologies" to acquire transcriptome data and clinical information from a study by Mariathasan involving BCa patients receiving immunotherapy (IMvigor 210, Phase II trial) (2).

Additional bioinformatic analyses. Secreted protein–encoding genes were obtained from the Human Protein Atlas (HPA) database (https://www.proteinatlas.org/). We next focused on *ELN* and evaluated its survival associations using univariate Cox proportional hazards regression alongside log-rank tests, implemented via the survival and survminer packages

in R. For pan-cancer evaluation, Hazard Ratios (HRs) and 95% Confidence Intervals were calculated to quantify the association between *ELN* expression and clinical outcomes, specifically OS and PFS, across various TCGA cohorts. The resulting HRs across cancer types were visualized using forest plots, where HR > 1 indicated that *ELN* expression was associated with an increased risk of mortality or disease progression. For Kaplan-Meier analysis, patients were stratified into high and low expression groups using the median expression value as a predefined threshold.

Additionally, gene sets (h.all.v2025.1.Hs.symbols.gmt, CLASSICAL_M1_VS_ALTER-NATIVE_M2_MACROPHAGE_DN.gmt, exhausted T cell signature) were sourced from the Molecular Signatures Database (MSigDB; http://www.gsea-msigdb.org/gsea/downloads.jsp) and previous study (Supplemental Table 7) (3-5). R packages imogimap and maftools were employed to calculate the EMT score and TMB for each sample. To assess immune infiltration, quanTIseq was utilized to quantify the proportions of immune cells within the transcriptome (6). Furthermore, associations between *ELN* and immune checkpoints were explored in the TCGA bladder cancer cohort as well as in the immunotherapy cohort from Mariathasan et al., using the TIMER2 and CAMOIP platforms (7, 8). Cellular composition within bulk RNA-seq datasets was estimated using BayesPrism (v2.0) for deconvolution analysis.

Visium spatial transcriptomics. The spatial transcriptomics dataset, which was based on the Visium Spatial Gene Expression platform and included four BCa tissue slices, was downloaded from the GEO database under the accession number GSE171351. We log-transformed the data using SCANPY's pp.log1p() fSunction and identified highly variable genes, selecting the top 2000 genes with the setting flavor = seurat_v3. Following preprocessing, spatial RNA-seq data analysis was conducted with the Python packages SCANPY (version 1.9.3), Squidpy (1.2.3), and Cell2location (v0.1.3) (9). Additionally, we employed mistyR (v1.2.1) to estimate the importance of each major cell type's abundance in explaining the abundance of other cell types. For the *TGFB1*-dependent ecological niche, we calculated estimated abundance values for *TGFB1* score derived from decoupleR package. Subsequently, we identified neighboring point clusters with cumulative abundance values exceeding 0.03 ($n > 5$) for further analysis (10). To further delineate the spatial microenvironments associated with ELN^+ CAFs, we classified Visium spots into ELN^+ or ELN^- categories using tangram-derived abundance estimates. Each spot was initially labeled according to whether ELN^+ or ELN^- CAFs constituted the dominant CAF subtype. To account for local spatial continuity and reduce noise driven by single-spot fluctuations, we applied a spatial k-nearest-neighbor (KNN) refinement procedure. Spatial coordinates were used to construct a k-d tree, from which the six nearest neighboring spots ($k = 6$) were identified for each index spot. Final ELN^+ or ELN^- assignments were determined by majority

voting within each neighborhood (⩾4 supporting neighbors), yielding spatially coherent ELN^+ and ELN^- domains that more accurately reflect underlying tissue architecture. To quantify the degree to which individual cell types contribute to local *TGFB1* signaling within these *ELN*-stratified microenvironments, we calculated a cell-type–specific weighted *TGFB1* score for each spot. This score was defined as the product of the tangram-predicted abundance of a given cell type and the spot-level *TGFB1* activity. This integrated metric captures both the presence of each cell population and its potential influence on *TGFB1*-dependent signaling within the tumor microenvironment in BCa.

References

1. Wu M, et al. Low doses of decitabine improve the chemotherapy efficacy against basal-like bladder cancer by targeting cancer stem cells. *Oncogene.* 2019;38(27):5425-39.
2. Mariathasan S, et al. TGFbeta attenuates tumour response to PD-L1 blockade by contributing to exclusion of T cells. *Nature.* 2018;554(7693):544-8.
3. Subramanian A, et al. Gene set enrichment analysis: a knowledge-based approach for interpreting genome-wide expression profiles. *Proc Natl Acad Sci U S A.* 2005;102(43):15545-50.
4. Guo X, et al. Global characterization of T cells in non-small-cell lung cancer by single-cell sequencing. *Nat Med.* 2018;24(7):978-85.
5. Hanzelmann S, et al. GSVA: gene set variation analysis for microarray and

RNA-seq data. *BMC Bioinformatics.* 2013;14:7.

6. Finotello F, et al. Molecular and pharmacological modulators of the tumor immune contexture revealed by deconvolution of RNA-seq data. *Genome Med.* 2019;11(1):34.
7. Lin A, et al. CAMOIP: a web server for comprehensive analysis on multi-omics of immunotherapy in pan-cancer. *Brief Bioinform.* 2022;23(3).
8. Li T, et al. TIMER2.0 for analysis of tumor-infiltrating immune cells. *Nucleic Acids Res.* 2020;48(W1):W509-W14.
9. Kleshchevnikov V, et al. Cell2location maps fine-grained cell types in spatial transcriptomics. *Nat Biotechnol.* 2022;40(5):661-71.
10. Kanemaru K, et al. Spatially resolved multiomics of human cardiac niches. *Nature.* 2023;619(7971):801-10.

Supplementary Figures

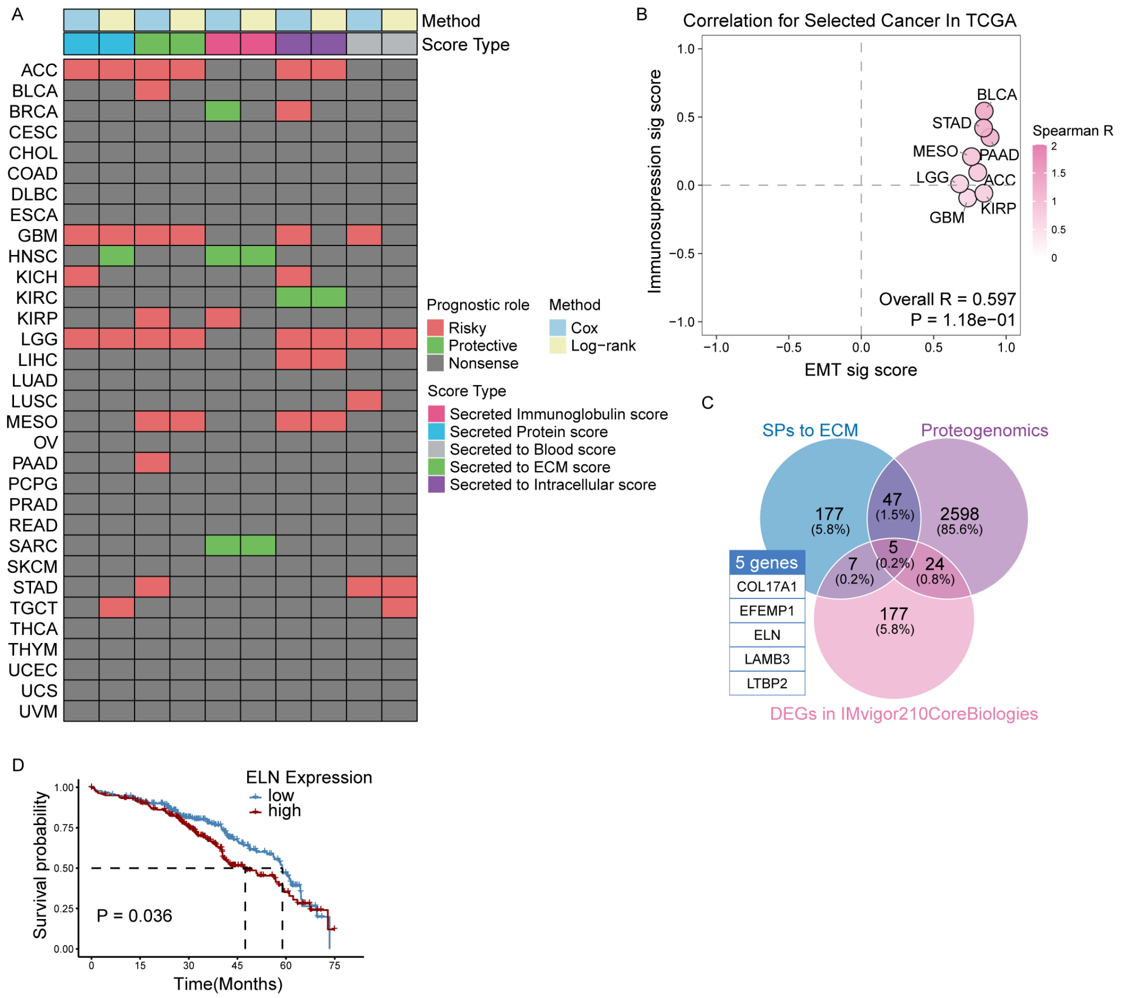

Figure S1: Pan-cancer profiling of secreted protein signatures and identification of candidates. (**A**) Heatmap displaying the prognostic significance of secreted protein-derived scores across TCGA pan-cancer cohorts. (**B**) Correlation between EMT and immunosuppression signature scores in selected TCGA cancer types. Spearman R and *P*-values are indicated. (**C**) Venn diagram showing the intersection of secreted ECM genes, proteogenomic datasets, and DEGs from the IMvigor210 cohort, identifying 5 candidate genes. (**D**) Kaplan-Meier analysis of PFS in the E-MTAB-4321 cohort, stratified by median *ELN* expression (high = 238, low = 238). *P*-values were

determined by log-rank test.

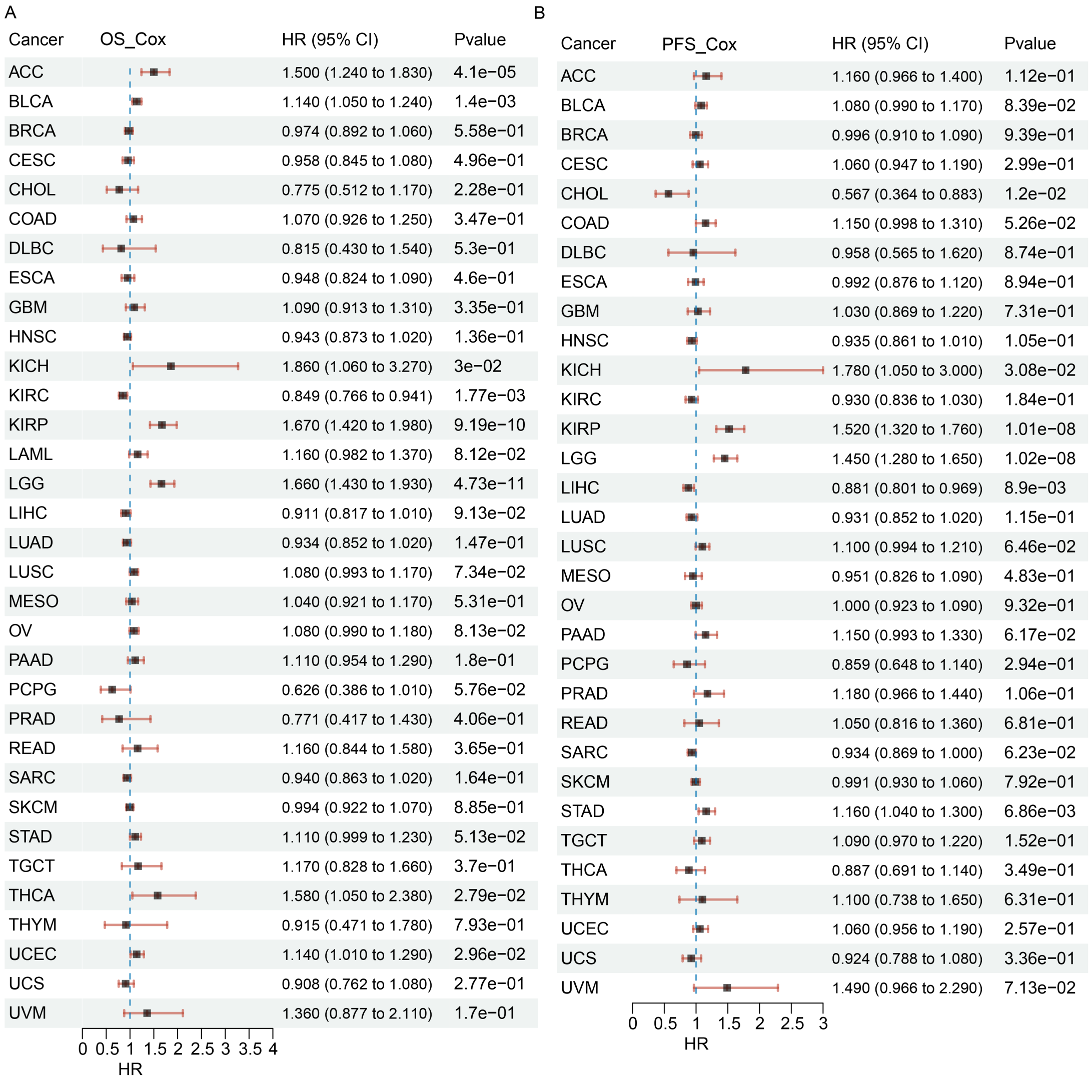

Figure S2: Pan-cancer prognostic significance of *ELN* expression in TCGA cohorts. (**A-B**) Forest plots illustrating the results of univariate Cox regression analysis for *ELN* expression across TCGA pan-cancer cohorts, evaluating overall survival (OS, **A**) and progression-free survival (PFS, **B**). *P*-values were calculated using the Cox proportional hazards model.

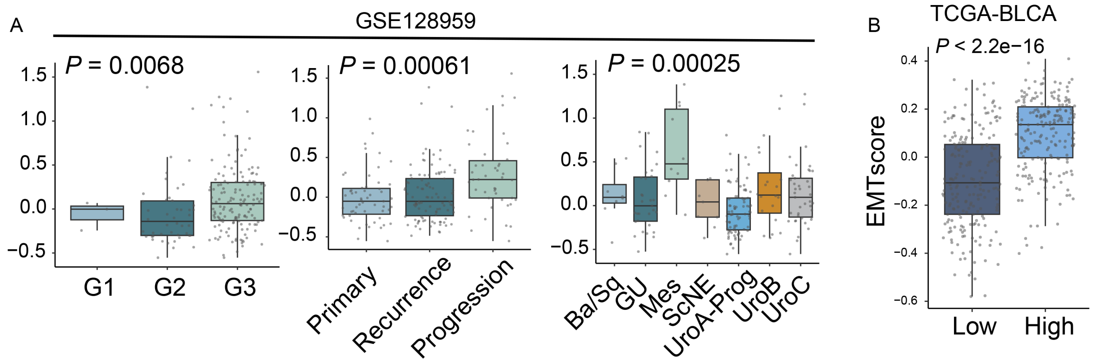

Figure S3: Differential expression of *ELN* across clinical grades, disease states, and molecular subtypes of BCa. (**A**) Box plots showing *ELN* expression levels in the GSE128959 cohort (n = 200) across different tumor grades, disease statuses, and molecular subtypes. *P*-values were determined by Kruskal-Wallis test. (**B**) Box plot comparing EMT signature scores between *ELN*_low and *ELN*_high groups (stratified by the median expression of *ELN*) in the TCGA-BLCA cohort (n = 408). *P*-values were determined by 2-tailed Mann-Whitney U test.

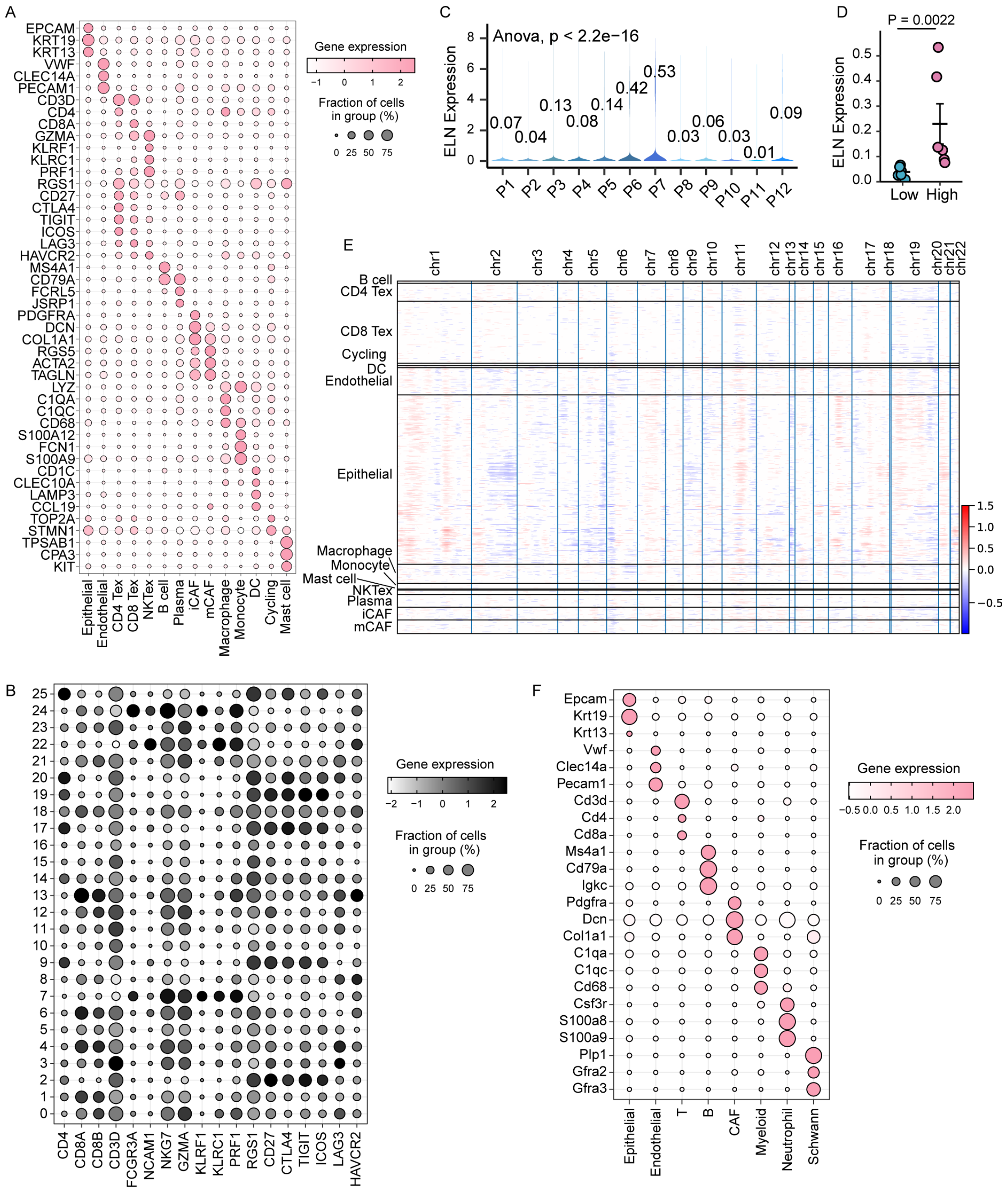

Figure S4: Single-cell transcriptomic characterization of *ELN* expression, cell-type annotation, and malignance identification in human and mouse BCa. (**A**) Dot plot showing marker-gene expression and cell-type annotation across major epithelial, stromal, and immune clusters from the integrated scRNA-seq dataset comprising 12 BCa patients. (**B**) Dot plot displaying T cell related marker expression across T cell clusters within resolution = 1, including

CD4$^+$ Tex, CD8$^+$ Tex, and NKT exhaustion subsets. (**C**) Violin plots showing *ELN* expression levels across patient samples (P1–P12) in the integrated human BCa single-cell dataset. (**D**) Boxplot comparing *ELN* expression between *ELN*_high and *ELN*_low patients. n = 6 per group. *P*-value was determined by 2-tailed Mann-Whitney U test. (**E**) CNV evaluated per cell by inferCNVpy. (**F**) Dot plot showing marker-gene expression in the BBN-induced mouse BCa single-cell dataset.

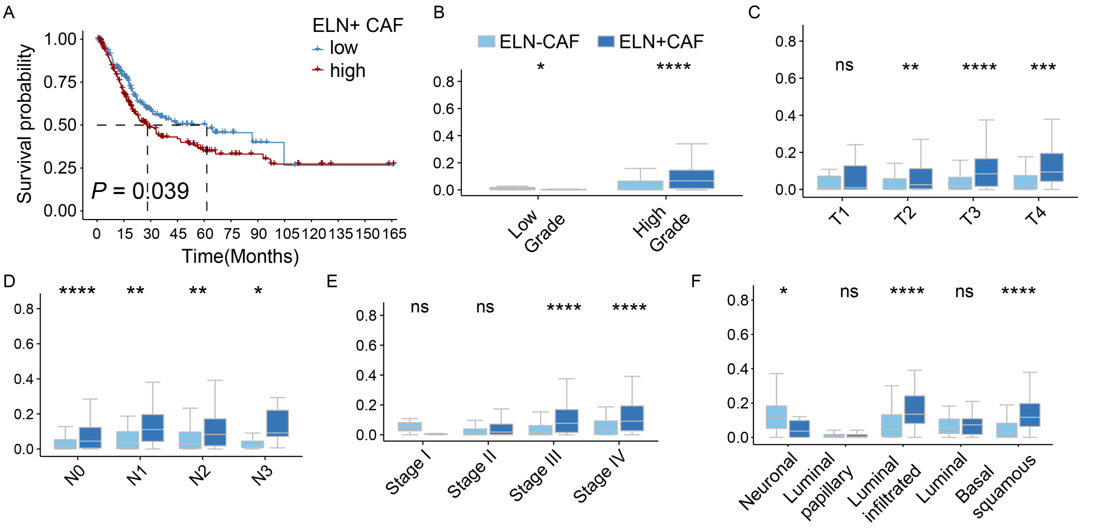

Figure S5: Association of ELN⁺ CAFs abundance with clinical features in TCGA-BLCA. (**A**) Kaplan–Meier curve (OS) for TCGA-BLCA patients stratified by high versus low proportions (median cut-off) of ELN^+ CAFs inferred by BayesPrism. (**B-F**) Boxplot comparing ELN^+ CAF abundance across tumor grade, clinical stage, molecular subtype, T stage, and N stage. *P*-values were calculated using two-tailed Mann–Whitney U test. ns, $P > 0.05$; $^*P < 0.05$, $^{**}P < 0.01$, $^{***}P < 0.001$, $^{****}P < 0.0001$.

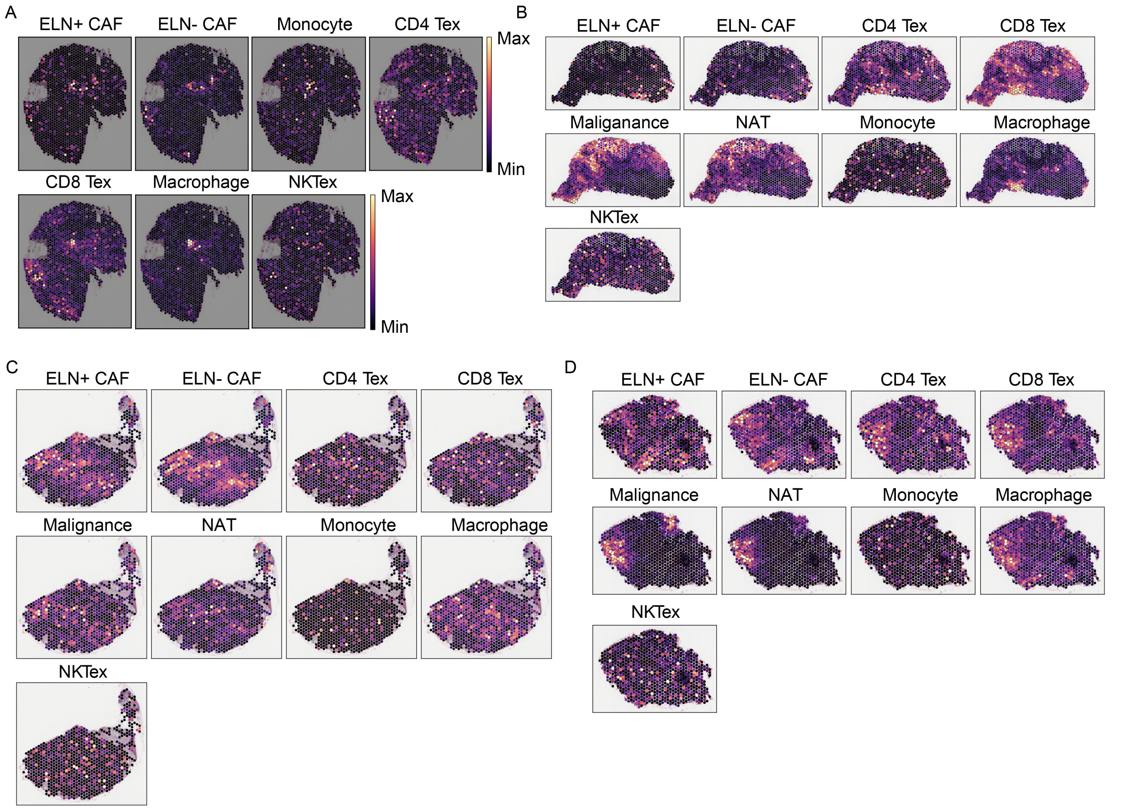

Figure S6: Spatial distribution of ELN$^+$ CAFs, immune subsets, and malignances across multiple BCa spatial transcriptomic samples. (**A**) Spatial transcriptomic maps showing the distribution of ELN$^+$ CAFs, ELN$^-$ CAFs, CD4$^+$ Tex, CD8$^+$ Tex, NKTex, monocytes, and macrophages in the first BCa tissue section.(**B-D**) Spatial distribution of ELN$^+$ CAFs, ELN$^-$ CAFs, CD4$^+$ Tex, CD8$^+$ Tex, NKTex, monocytes, macrophages, malignances, and NAT regions in BCa section of P2-P4.

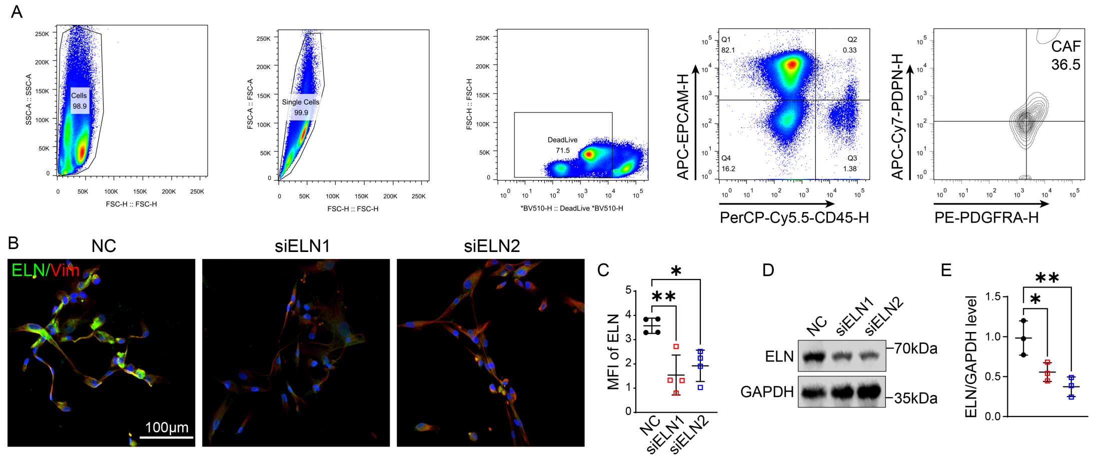

Figure S7: Flow-cytometric isolation of primary human BCa CAFs and validation of *ELN* knockdown efficiency. (**A**) Gating strategy for flow-cytometric isolation of primary CAFs from human BCa tissues. CAFs were identified as viable, singlet, Dead/Live$^-$, EPCAM$^-$CD45$^-$, and PDPN$^+$PDGFRA$^+$ cells. (**B-C**) Representative immunofluorescence images (**B**) and MFI quantification (**C**) of ELN (green) and Vimentin (red) in primary CAFs transfected with negative control siRNA (NC) or *ELN*-targeting siRNAs (siELN1, siELN2). n = 4 for each group. *P*-values were determined by one-way ANOVA followed by Tukey's multiple comparisons test. (**D-E**) Immunoblot analysis (**D**) and densitometric quantification (**E**) of ELN protein levels normalized to GAPDH in CAFs following siRNA transfection. n = 3 for each group. *P*-values were determined by one-way ANOVA followed by Tukey's multiple comparisons test. ns, $P > 0.05$; $^*P < 0.05$, $^{**}P < 0.01$, $^{***}P < 0.001$, $^{****}P < 0.0001$.

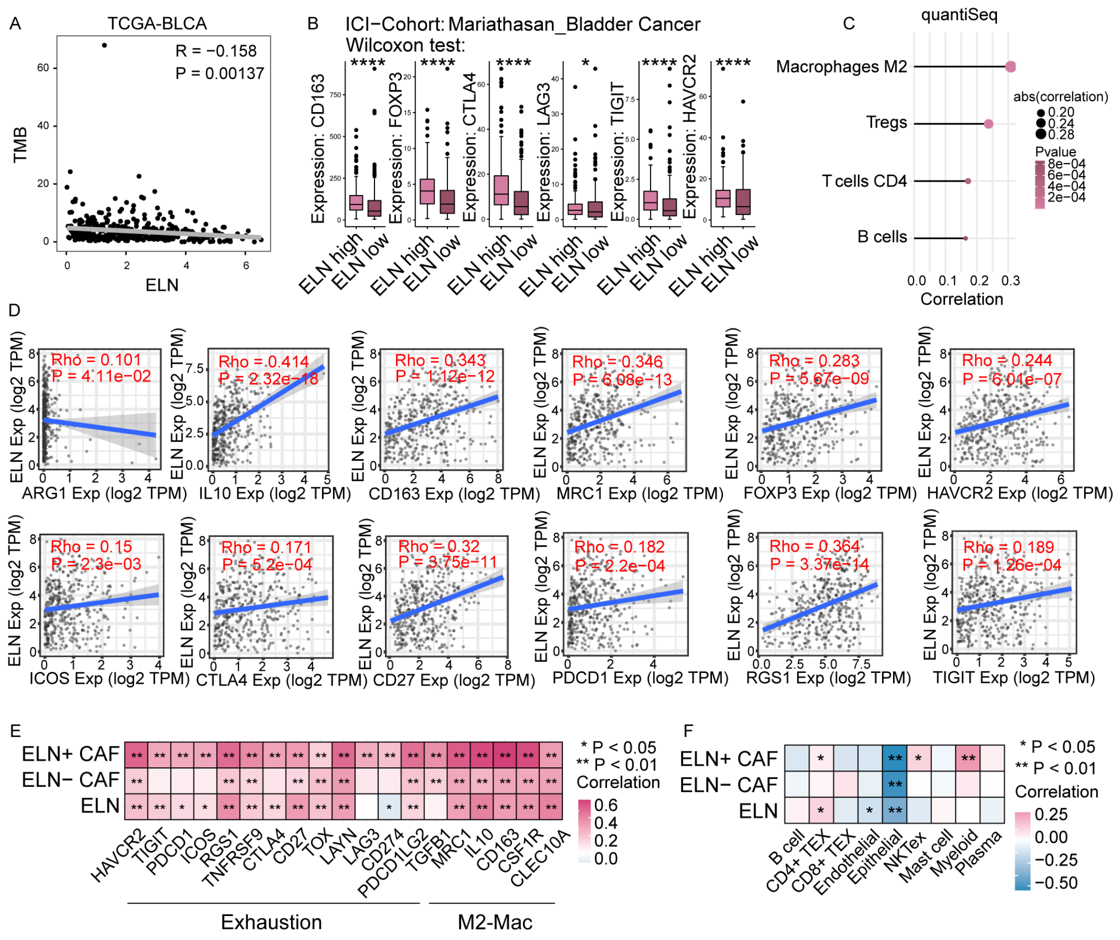

Figure S8: Associations between *ELN* expression, ELN$^+$ CAFs, tumor immunogenicity, and immunosuppressive signatures in BCa. (**A**) Correlation between *ELN* expression and TMB in the TCGA-BLCA cohort. (**B**) Expression of immunosuppressive markers (*CD163*, *FOXP3*, *CTLA4*, *LAG3*, *TIGIT*, *HAVCR2*) in *ELN*-high versus *ELN*-low groups within the Mariathasan immunotherapy cohort. *P*-values were determined by the Wilcoxon rank-sum test. (**C**) Correlation of *ELN* expression with immune cell abundances (quantiSeq deconvolution) in TCGA-BLCA. (**D**) Correlation plots of *ELN* expression with M2-like macrophage markers (*ARG1*, *IL10*, *CD163*, *MRC1*) and Tex markers (*FOXP3*, *HAVCR2*, *ICOS*, *CTLA4*, *CD27*, *PDCD1*, *RGS1*, *TIGIT*) in TCGA-BLCA. (**E-F**) Heatmaps displaying correlations of *ELN*

expression, ELN^+ CAFs, and ELN^- CAFs with Tex/M2-macrophage markers (**E**) and diverse immune/stromal cell subsets (**F**) in TCGA-BLCA. ns, $P > 0.05$; $^*P < 0.05$, $^{**}P < 0.01$, $^{***}P < 0.001$, $^{****}P < 0.0001$.

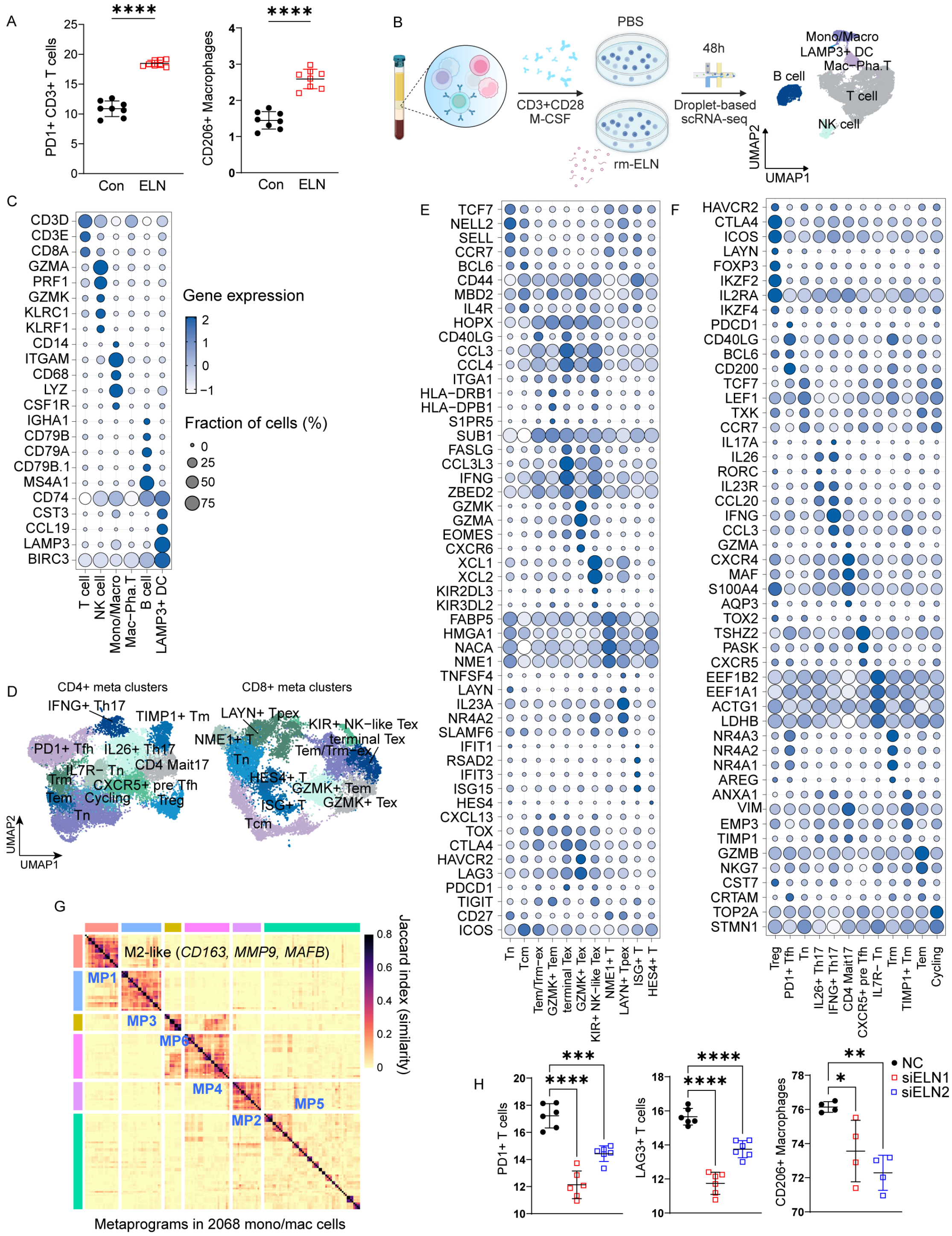

Figure S9: Single-cell transcriptomic profiling of rm-ELN–stimulated human PBMCs and functional validation of ELN-dependent immune modulation. (**A**) Flow-cytometric quantification of PD1$^+$CD3$^+$ T cells and CD206$^+$ macrophages following rm-ELN stimulation. *P*-values were determined by 2-tailed Student's t test. (**B**) Experimental workflow for scRNA-seq analysis

of human PBMCs stimulated with rm-ELN. (**C**) Dot plot showing canonical marker gene expression across major immune lineages. (**D**) UMAP visualization of refined $CD4^+$ and $CD8^+$ T cell metaclusters. (**E-F**) Dot plots displaying expression of marker genes across identified $CD4^+$ (**E**) and $CD8^+$ (**F**) T cell subsets. (**G**) NMF metaprogram analysis of 2,068 monocyte/macrophage cells, identifying M2-like signatures (CD163, MMP9, MAFB). (**H**) Flow-cytometric quantification of $PD1^+$ T cells, $LAG3^+$ T cells, and $CD206^+$ macrophages in co-culture assays with CAFs transfected with NC or ELN siRNAs. n = 4-6 for each group. *P*-values were determined by one-way ANOVA followed by Tukey's multiple comparisons test. ns, $P > 0.05$; $^*P < 0.05$, $^{**}P < 0.01$, $^{***}P < 0.001$, $^{****}P < 0.0001$.

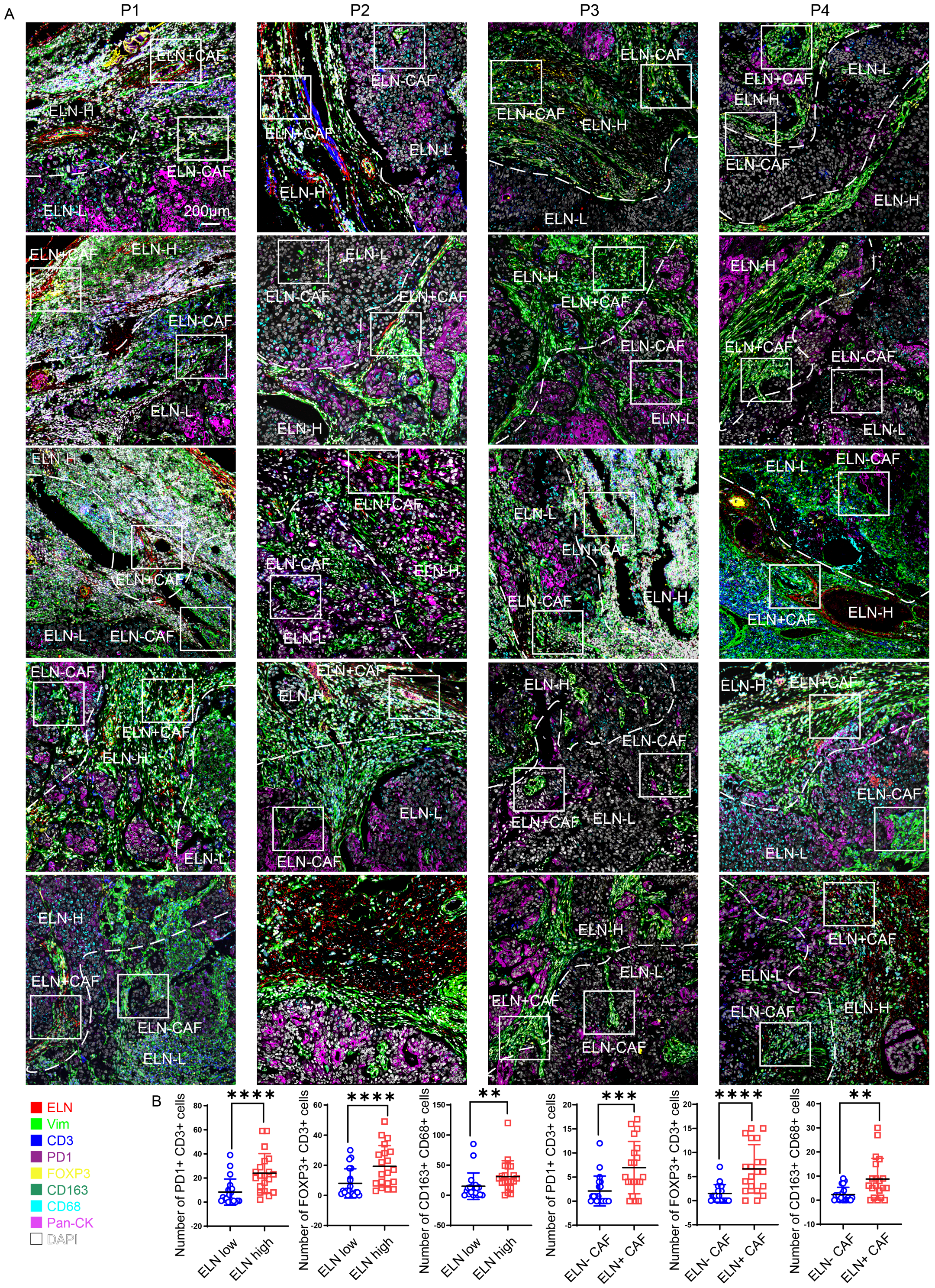

Figure S10: Multiplex immunofluorescence analysis of ELN-high regions and ELN⁺ CAF niches in human BCa tissues. (**A**) Multiplex immunofluorescence images from four human BCa patients (P1–P4) displaying the spatial organization of ELN, Vimentin, CD3, PD1, FOXP3, CD163, CD68,

Pan-CK, and DAPI. Representative ELN-high (ELN-H), ELN-low (ELN-L), and ELN^+ CAF-rich regions are highlighted. (**B**) Quantification of $PD1^+CD3^+$ T cells, $FOXP3^+CD3^+$ Tregs, and $CD163^+$ $CD68^+$ M2-like macrophages comparing ELN-high versus ELN-low regions, and ELN^+ CAF-associated versus ELN^- CAF-associated niches. Each data point represents a single FOV from clinical samples. *P*-values were determined by two-tailed Student's t test. ns, $P > 0.05$; $^*P < 0.05$, $^{**}P < 0.01$, $^{***}P < 0.001$, $^{****}P < 0.0001$.

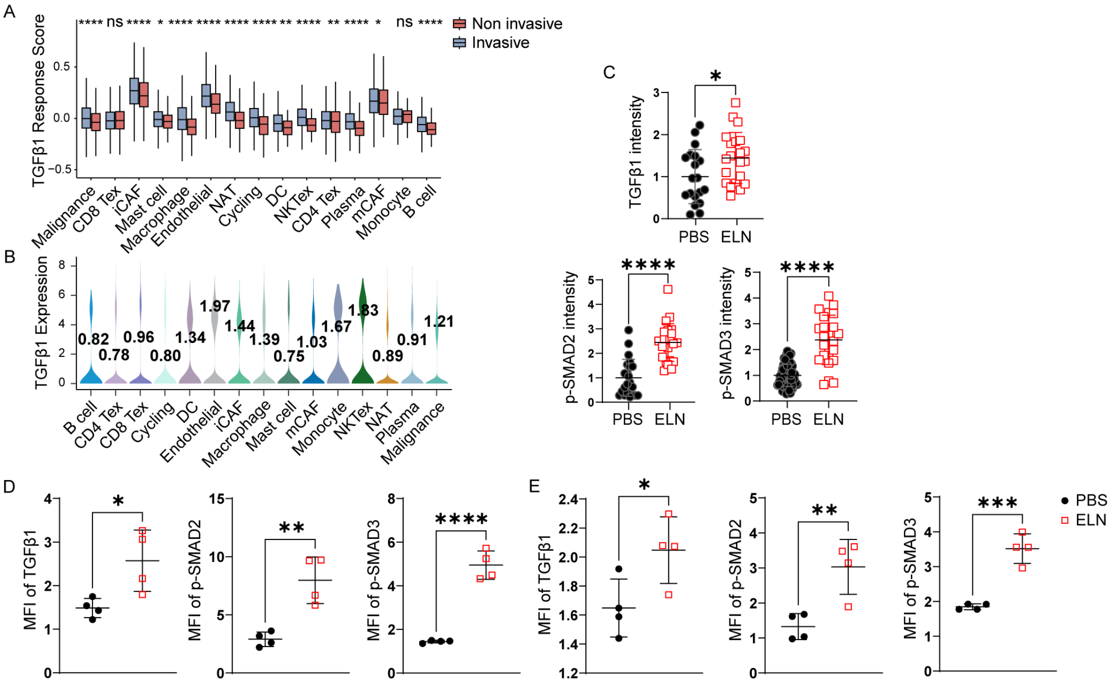

Figure S11: Profiling of *TGFB1* signaling across BCa cell subsets and its induction by ELN. (**A**) Box plots of *TGFB1* response scores across major cell types in invasive versus noninvasive tumors. P-values were determined by two-tailed Mann-Whitney U test. (**B**) Violin plots displaying *TGFB1* expression across major cell populations in human BCa scRNA-seq data. (**C**) Quantified fluorescence intensity of TGFB1, p-SMAD2, and p-SMAD3 in BCa organoids treated with PBS or rm-ELN. Each dot represents an individual organoid. n = 19-68 for each group. P-values were determined by two-tailed Student's t test. (**D-E**) MFI of TGFB1, p-SMAD2, and p-SMAD3 in T cells (**D**) and macrophages (**E**) following rm-ELN treatment. n = 4 for each group. P-values were determined by two-tailed Student's t test. ns, P > 0.05; *P < 0.05, **P < 0.01, ***P < 0.001, ****P < 0.0001.

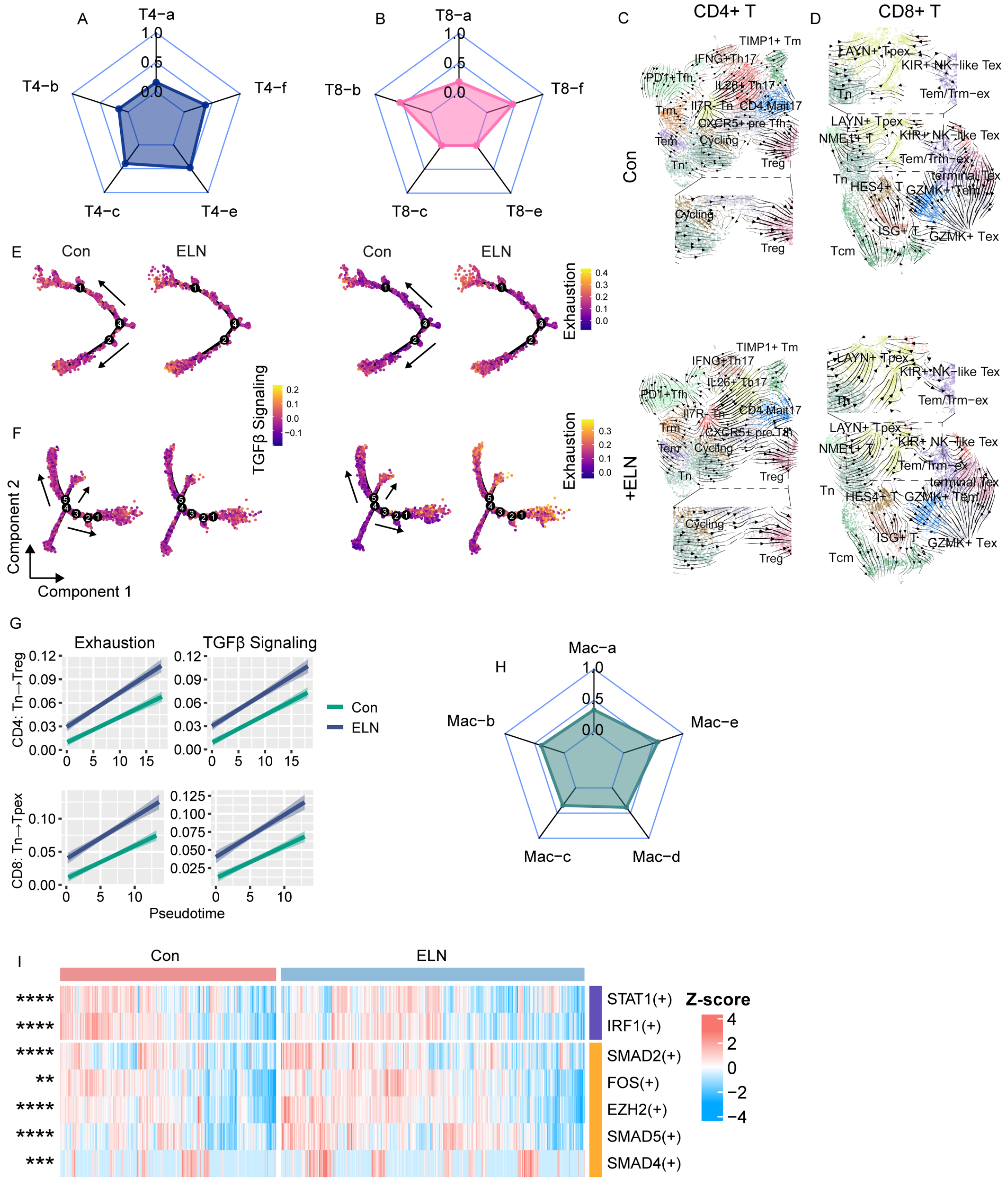

Figure S12: ELN-driven transcriptional polarization of T cells and macrophages. (**A-B**) Cytokine-response dictionary-based radar plots showing the classification of CD4⁺ (**A**) and CD8⁺ (**B**) T cell states in rm-ELN–treated PBMCs. (**C-D**) RNA-velocity streamlines overlaid on UMAP embeddings of CD4⁺ (**C**) and CD8⁺ (**D**) T cells, illustrating differentiation vectors under control and rm-ELN conditions. (**E-F**) Pseudotime-aligned differentiation trajectories

along the CD4^{+} (from Tn to Treg) and CD8^{+} (from Tn to Tpex) lineages, displaying progressive increases in *TGFB1*-associated (left) and exhaustion-related (right) transcriptional activity. (**G**) Quantification of pathway activity scores showing enhanced *TGFB1* signaling and exhaustion along both CD4^{+} and CD8^{+} differentiation trajectories after rm-ELN exposure. (**H**) Cytokine-response dictionary classification of monocyte/macrophage transcriptional states. (**I**) Heatmap of pySCENIC-based regulon activity analysis identifying the activation of M1-associated (purple) or M2-associated (orange) transcription factors following rm-ELN stimulation. *P*-values were calculated using two-tailed Mann–Whitney U test. ns, $P > 0.05$; *$P < 0.05$, **$P < 0.01$, ***$P < 0.001$, ****$P < 0.0001$.

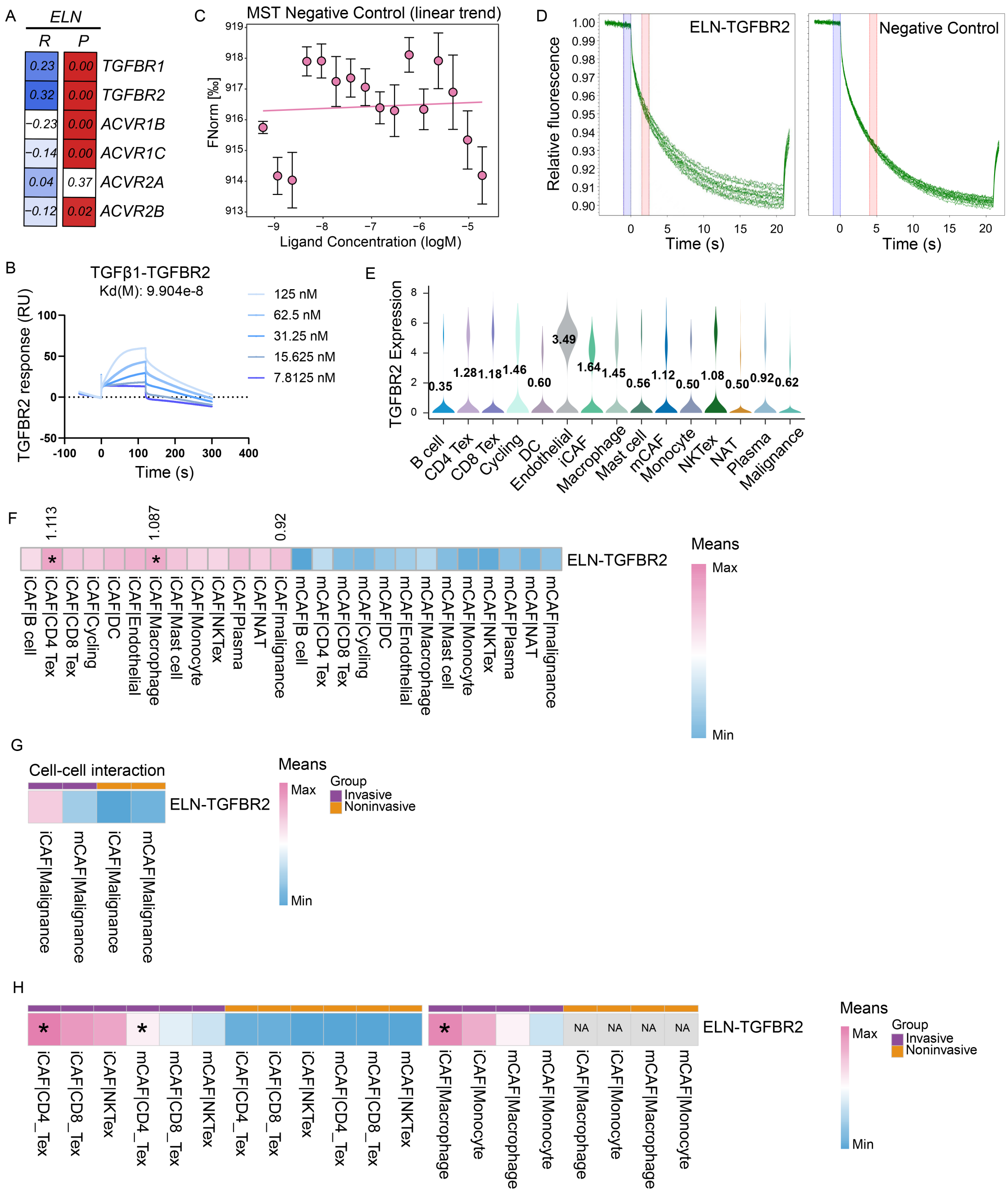

Figure S13: ELN–TGFBR2 interactions and cell type–specific expression patterns. (**A**) Correlation heatmap showing associations between *ELN* expression and TGFB receptor family members in the TCGA-BLCA cohort. Spearman R and *P*-values are indicated. (**B**) SPR sensorgrams depicting the binding kinetics of rm-TGFβ1 to immobilized TGFBR2 across increasing concentrations. (**C**) MST negative-control assay showing no significant binding

trend. (**D**) Representative MST traces displaying the interaction between ELN and TGFBR2 compared with the negative control. (**E**) Violin plots displaying *TGFBR2* expression levels across major cell populations in human BCa scRNA-seq data. (**F–H**) CellPhoneDB analysis displaying the strength of ELN–TGFBR2 interactions from iCAFs or mCAFs to other cell types in total (**F**), invasive and noninvasive (**G-H**) BCa samples. ns, $P > 0.05$; *$P < 0.05$, **$P < 0.01$, ***$P < 0.001$, ****$P < 0.0001$.

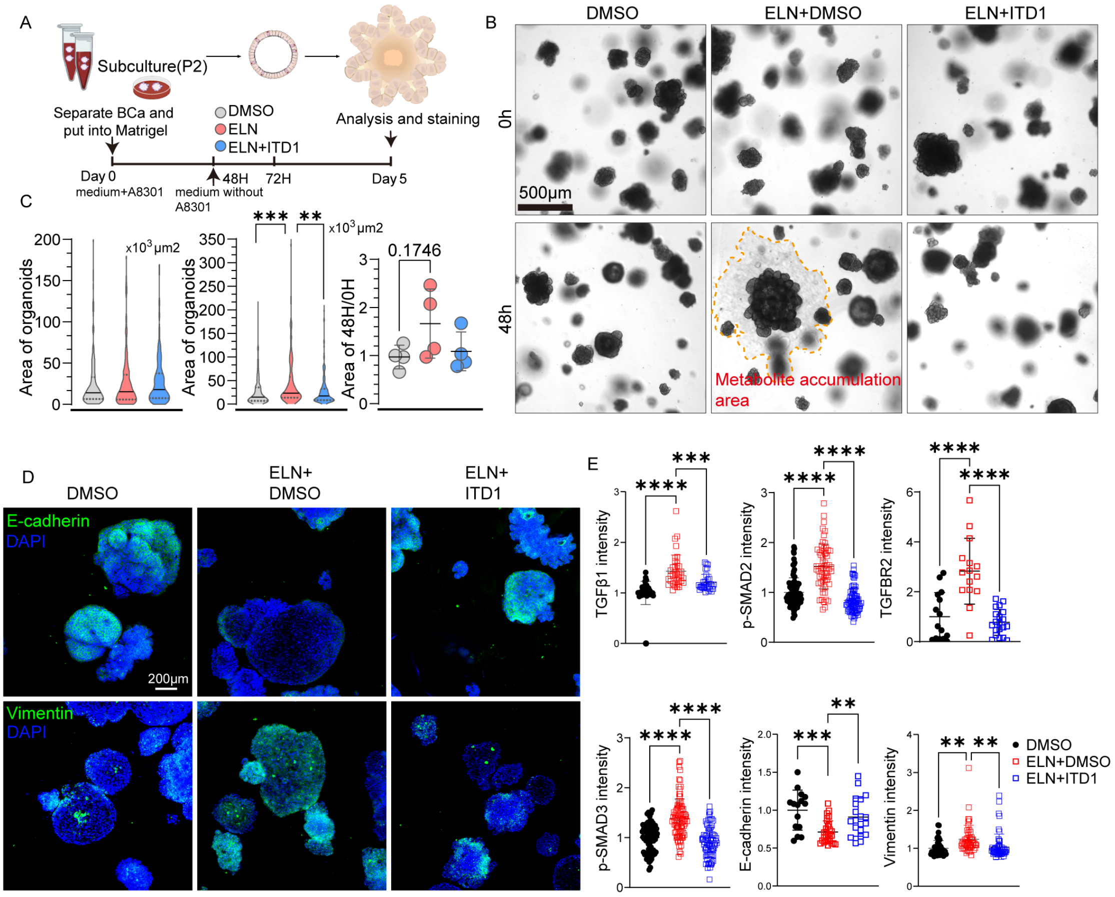

Figure S14. ELN drives TGFB1 signaling and EMT in BCa organoids via TGFBR2. (**A**) Schematic overview of the experimental workflow for BCa organoid culture in Matrigel and subsequent treatments with DMSO, ELN, or ELN + ITD1. (**B-C**) Representative bright-field images (**B**) and quantification of organoid area distributions (**C**) at 0 h and 48 h across treatment conditions. Note the formation of a metabolite accumulation area (orange dashed lines) in the ELN-treated group. n = 137-168 for each group. *P*-values were determined by one-way ANOVA followed by Tukey's multiple comparisons test. (**D-E**) Representative immunofluorescence images (**D**) and MFI quantification (**E**) of TGFβ1, p-SMAD2, TGFBR2, p-SMAD3, E-cadherin, and Vimentin in BCa organoids after the indicated treatments. n = 15-96 for each group. *P*-values

were determined by one-way ANOVA followed by Tukey's multiple comparisons test. ns, $P > 0.05$; $^{*}P < 0.05$, $^{**}P < 0.01$, $^{***}P < 0.001$, $^{****}P < 0.0001$.

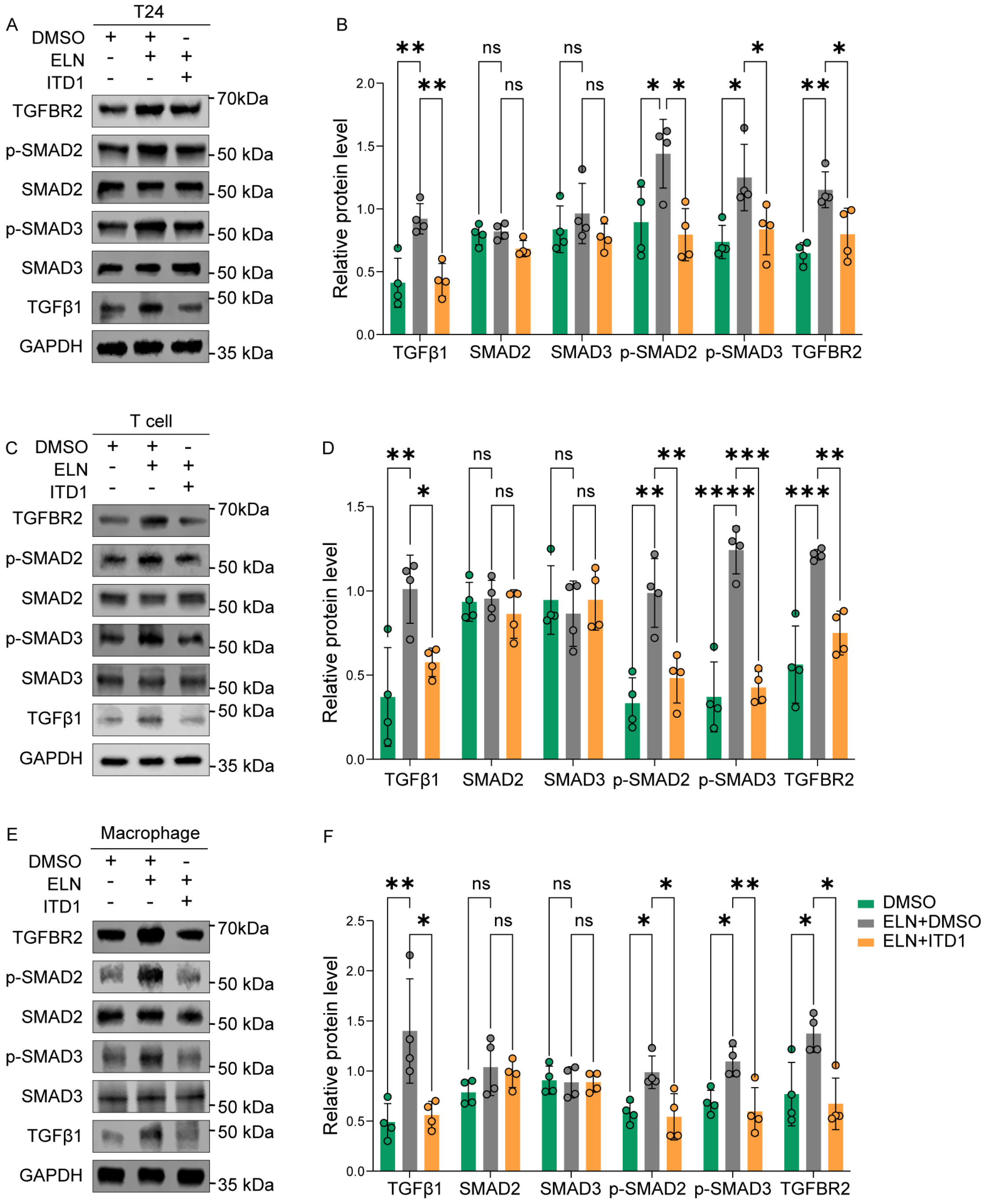

Figure S15. ELN activates TGFB1 signaling through TGFBR2 in BCa cells and immune subsets. (**A-B**) Immunoblot analysis (**A**) and densitometric quantification (**B**) of TGFβ1, SMAD2, SMAD3, p-SMAD2, p-SMAD3, and TGFBR2 protein levels in T24 cells treated with DMSO, ELN + DMSO, or ELN + ITD1. (**C-D**) Immunoblot analysis (**C**) and quantification (**D**) of TGFβ1 signaling components in purified human CD3^{+} T cells following indicated

treatments. (**E-F**) Immunoblot analysis (**E**) and quantification (**F**) of TGFB1 signaling components in monocyte-derived macrophages following indicated treatments. GAPDH was used as the loading control. n = 4 for each group. *P*-values were determined by one-way ANOVA followed by Tukey's multiple comparisons test. ns, $P > 0.05$; *$P < 0.05$, **$P < 0.01$, ***$P < 0.001$, ****$P < 0.0001$.

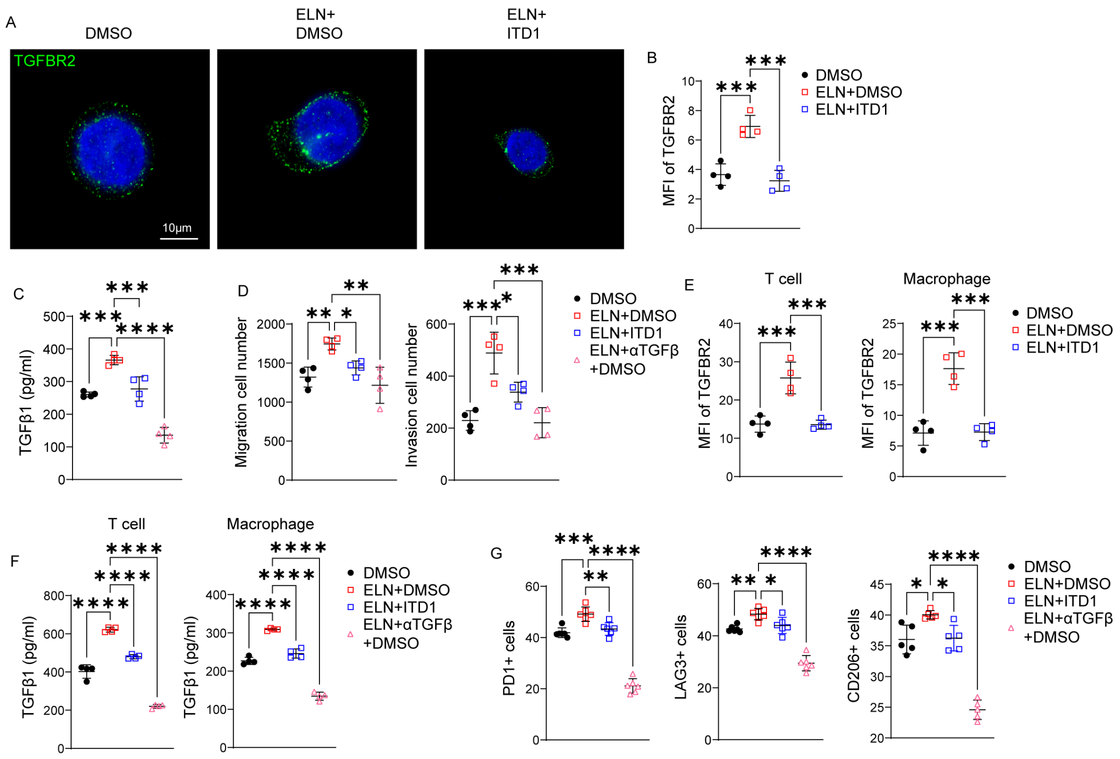

Figure S16: Modulation of TGFBR2 expression, TGFB1 signaling, and immune phenotypes by ELN and TGFBR2 inhibition. (**A-B**) STED super-resolution images (**A**) and MFI quantification (**B**) of TGFBR2 (green) in T24 cells treated with DMSO, ELN + DMSO, or ELN + ITD1. n = 4 for each group. *P*-values were determined by one-way ANOVA followed by Tukey's multiple comparisons test. (**C-D**) ELISA measurement of secreted total TGFβ1 levels (**C**) and transwell quantification of migration and invasion (**D**) in BCa cells under indicated treatments. n = 4 for each group. *P*-values were determined by one-way ANOVA followed by Tukey's multiple comparisons test. (**E-F**) MFI quantification of TGFBR2 (**E**) and secreted total TGFβ1 levels (**F**) in purified human T cells and macrophages stimulated with ELN ± ITD1 or anti-TGFβ. n = 4 for each group. *P*-values were determined by one-way ANOVA followed by Tukey's multiple comparisons test. (**G**) Flow-cytometric frequencies of PD1^{+} T

cells, LAG3$^+$ T cells, and CD206$^+$ macrophages following indicated treatments. n = 5-6 for each group. *P*-values were determined by one-way ANOVA followed by Tukey's multiple comparisons test. ns, $P > 0.05$; *$P < 0.05$, **$P < 0.01$, ***$P < 0.001$, ****$P < 0.0001$.

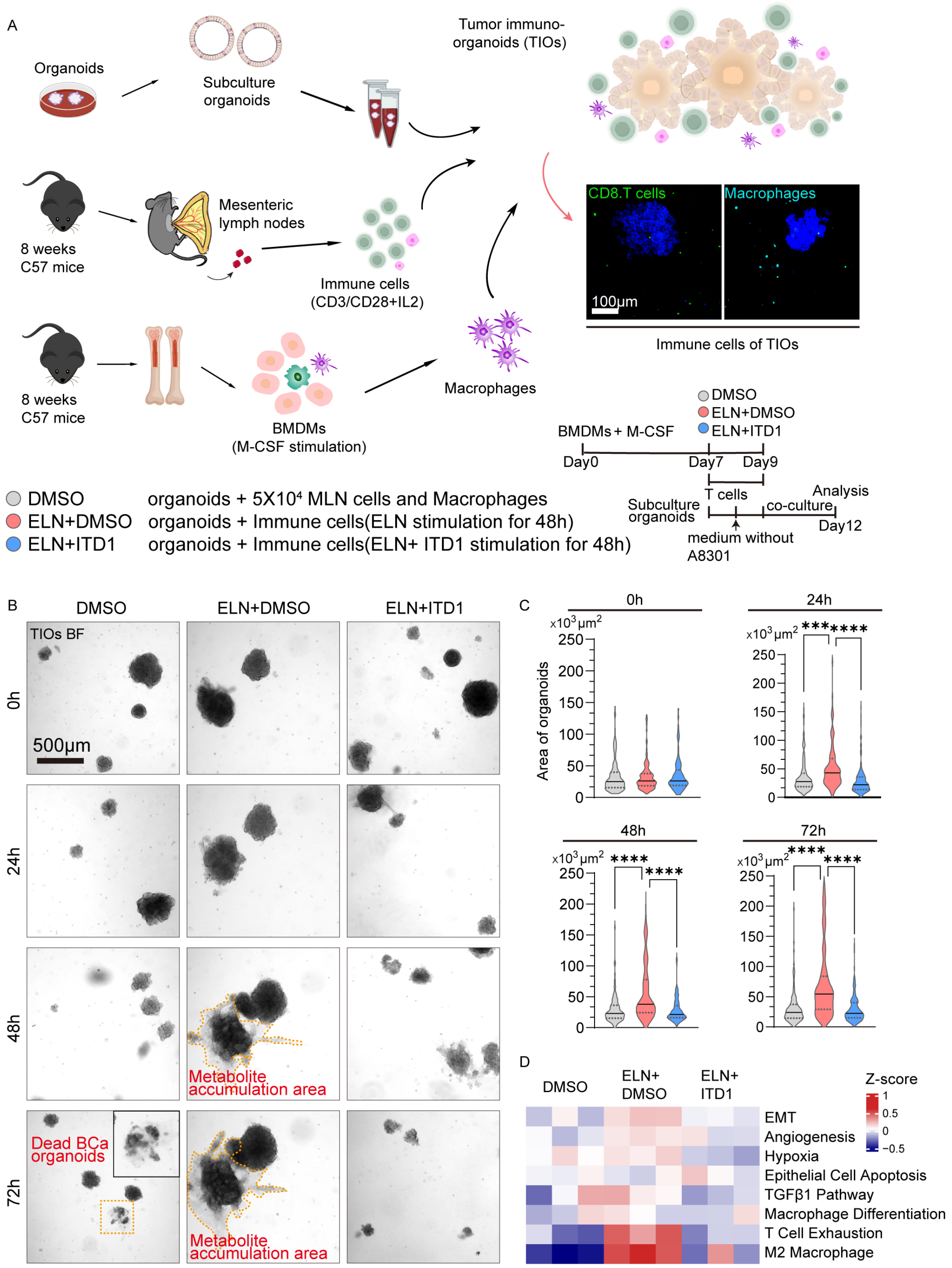

Figure S17: Evaluation of ELN-driven immune suppression in a 3D TIO model. (**A**) Schematic illustration of the experimental workflow for establishing the TIO model. Primary BCa organoids were subcultured and co-cultured with MLN-derived CD8^{+} T cells and BMDMs from C57BL/6 mice. TIOs were treated

with DMSO, ELN + DMSO, or ELN + ITD1 as indicated in the timeline. Representative immunofluorescence images (right) show the spatial distribution of $CD8^+$ T cells (green) and macrophages (cyan) within TIOs. (**B-C**) Representative bright-field images (**B**) and quantification of organoid area (**C**) across indicated treatment groups from 0 to 72 hours. Note the accumulation of metabolites and dead BCa organoids (orange dashed lines/insets) in the ELN + DMSO group. n = 95-131 for each group. *P*-values were determined by one-way ANOVA followed by Tukey's multiple comparisons test. (**D**) Heatmap displaying the Z-scores of selected transcriptomic signatures, including EMT, Angiogenesis, *TGFB1* pathway, and M2-macrophage polarization, across treatment groups in the TIO model. ns, $P > 0.05$; $*P < 0.05$, $**P < 0.01$, $***P < 0.001$, $****P < 0.0001$.

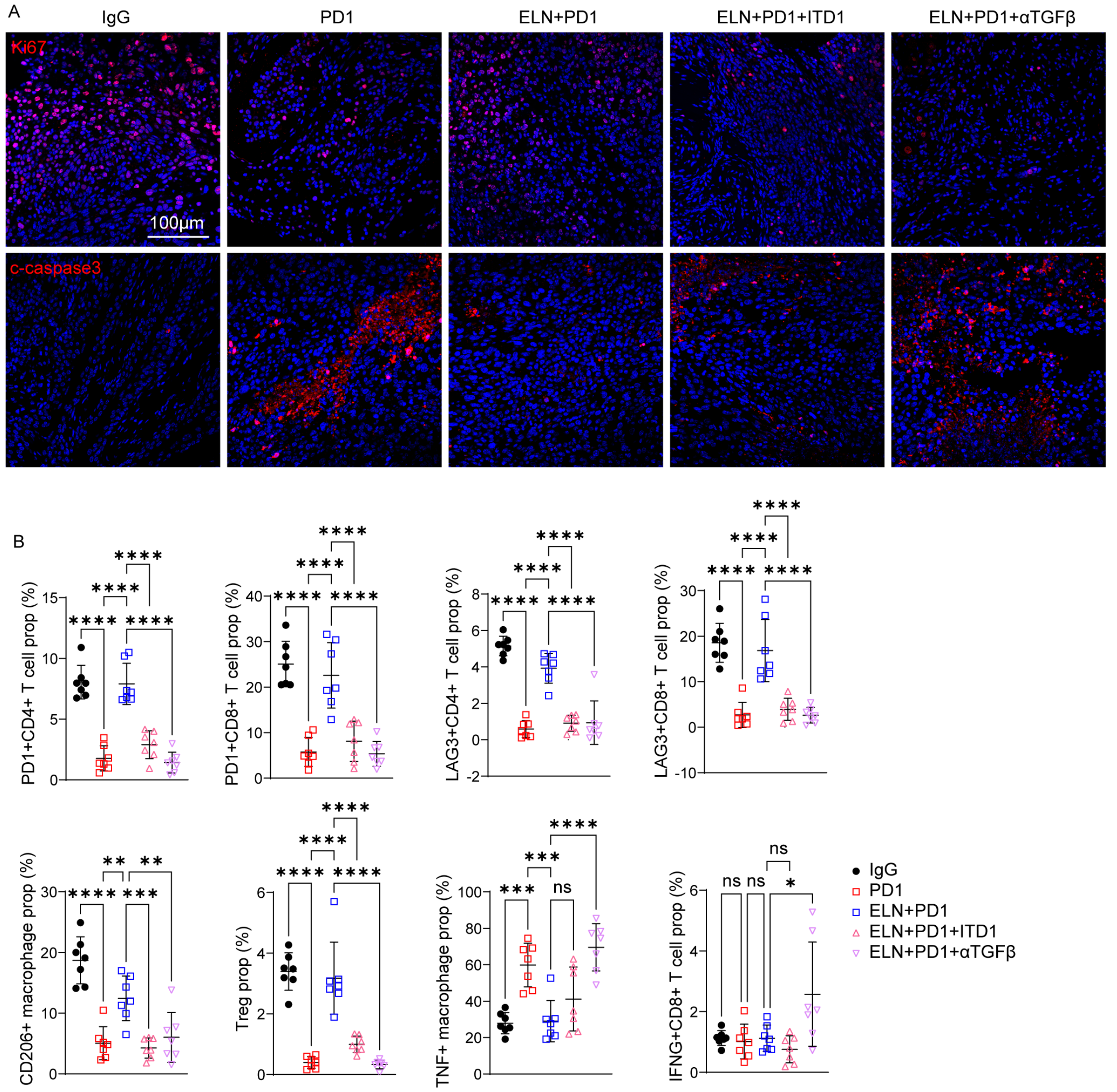

Figure S18: ELN impairs anti–PD-1 therapy response by modulating the immune microenvironment in orthotopic BCa model. (**A**) Representative immunofluorescence images of Ki67 and cleaved-caspase3 in MB49 orthotopic tumor sections across treatment groups. (**B**) Flow-cytometric quantification of tumor-infiltrating immune subsets, including PD1^{+} and LAG3^{+} T cells, CD206^{+} or TNF^{+} macrophages, and Tregs, following the indicated treatments. n = 7 for each group. *P*-values were determined by one-way ANOVA followed by Tukey's multiple comparisons test. ns, $P > 0.05$; *$P < 0.05$, **$P < 0.01$, ***$P < 0.001$, ****$P < 0.0001$.
