# Supplementary material for: ELN orchestrates prometastatic and immunosuppressive niche in bladder cancer via TGFB1 autocrine signaling
Source: JCI Insight. 2026 May 22;11(10):e194700. doi: 10.1172/jci.insight.194700 (PMC13232729; doi:10.1172/jci.insight.194700)

Figure 2, M

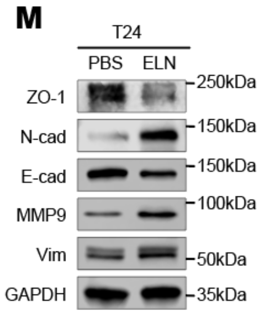

The Image captured by the LICOR instrument processed using Image Studio Ver 5.2.

Uncropped/unedited images↓

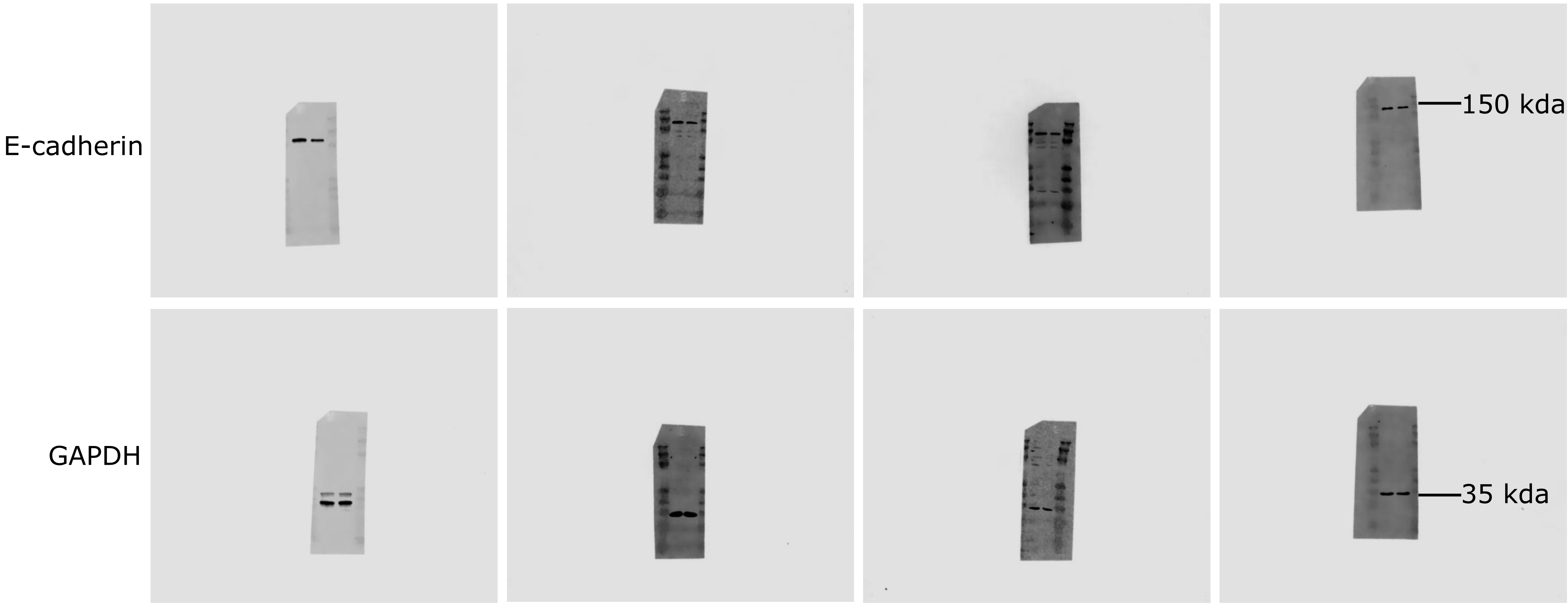

Figure 2, M

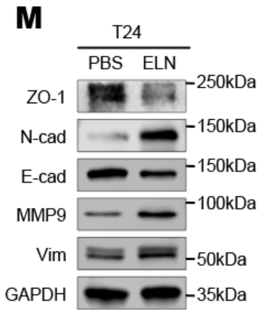

The Image captured by the LICOR instrument processed using Image Studio Ver 5.2.

Uncropped/unedited images↓

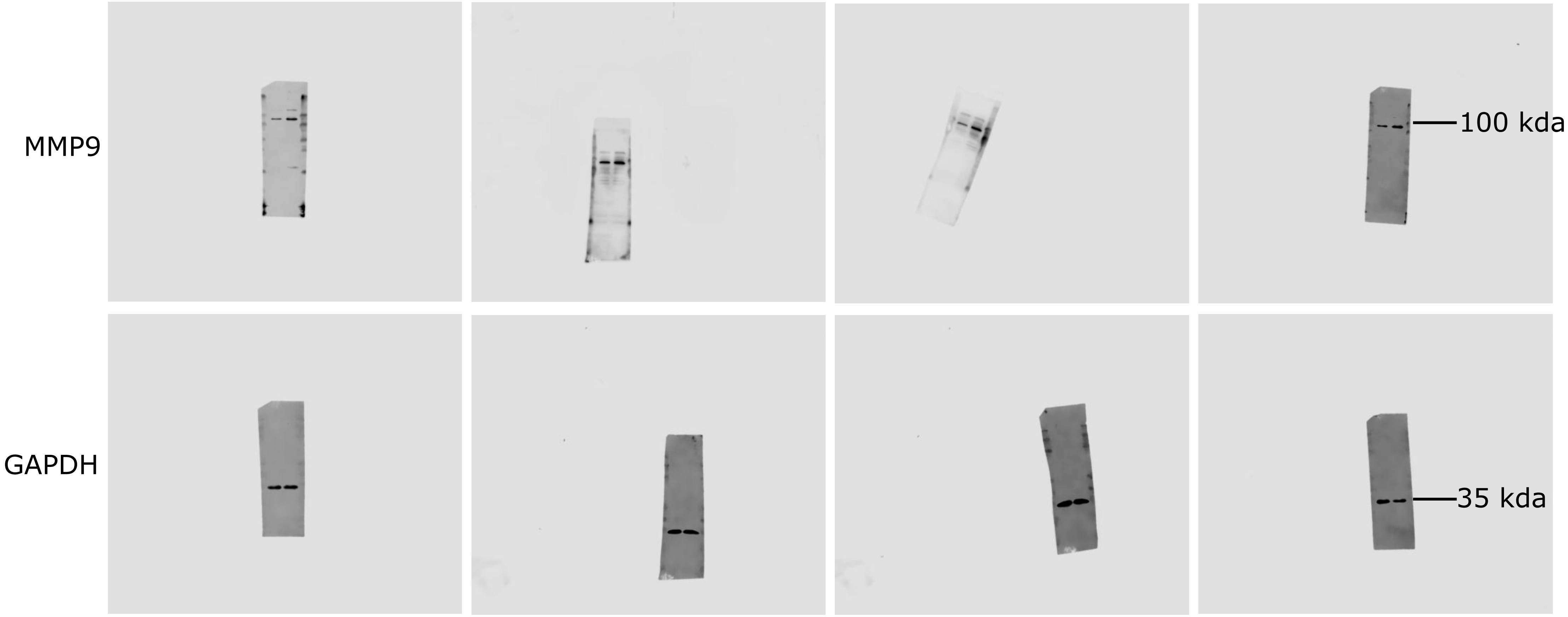

Figure 2, M

The Image captured by the LICOR instrument processed using Image Studio Ver 5.2.

Uncropped/unedited images↓

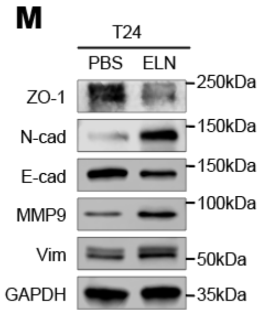

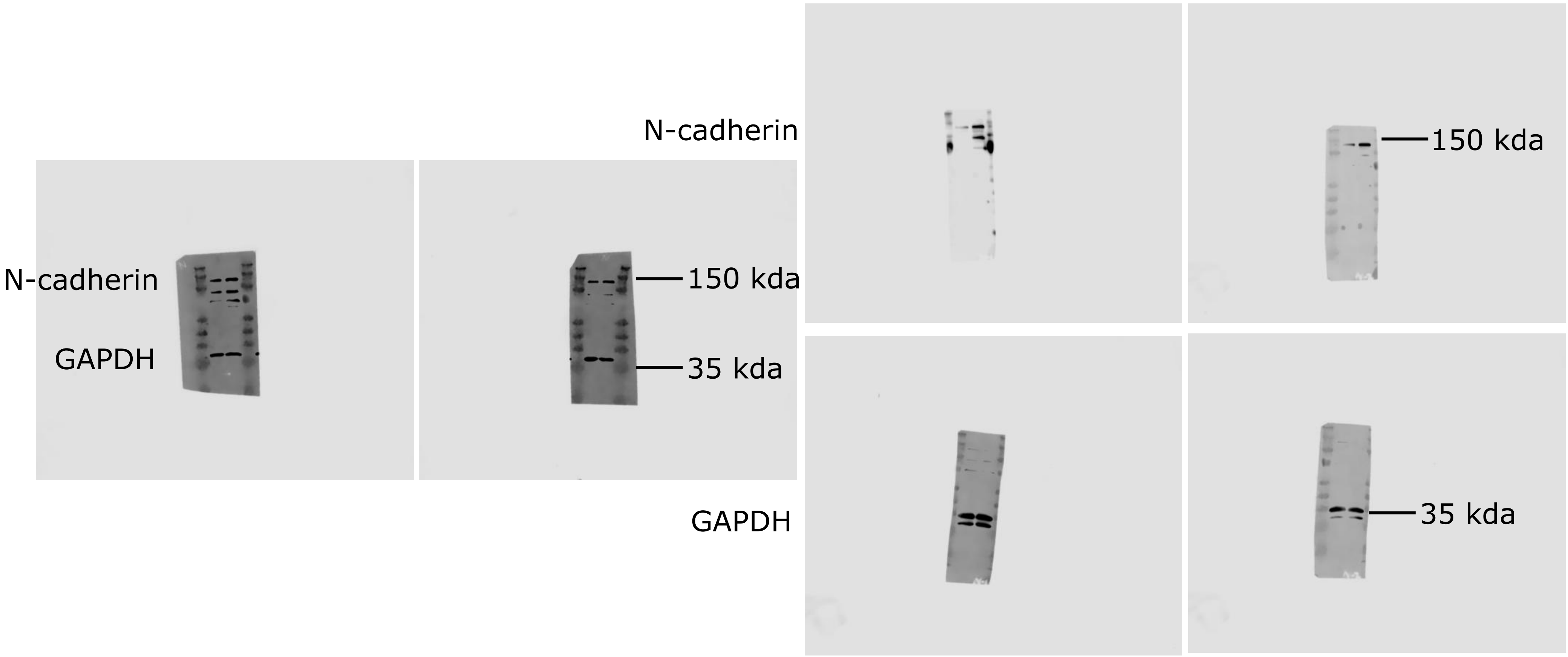

Figure 2, M

The Image captured by the LICOR instrument processed using Image Studio Ver 5.2.

Uncropped/unedited images↓

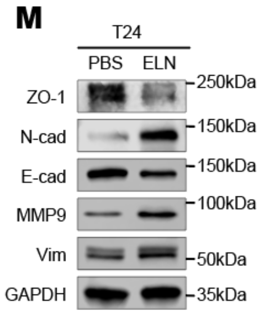

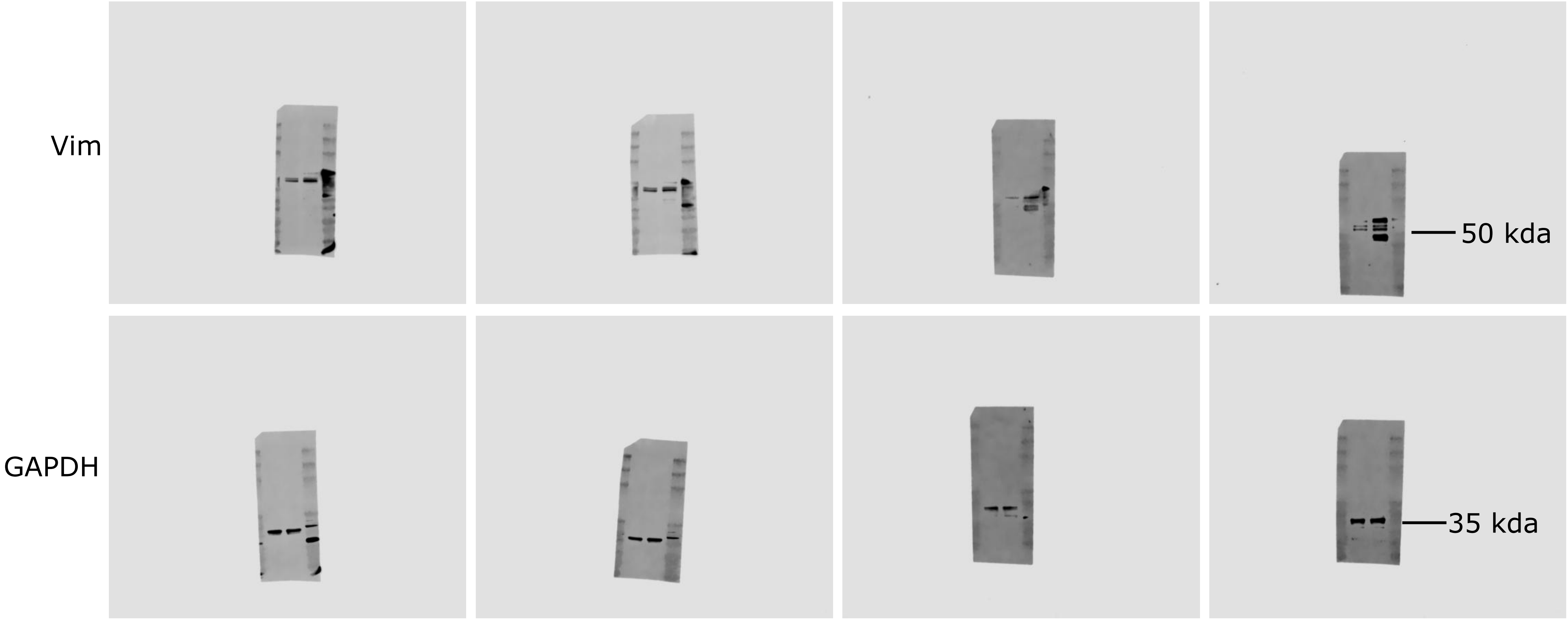

Figure 2, M

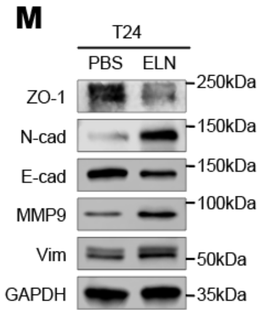

The Image captured by the LICOR instrument processed using Image Studio Ver 5.2.

Uncropped/unedited images↓

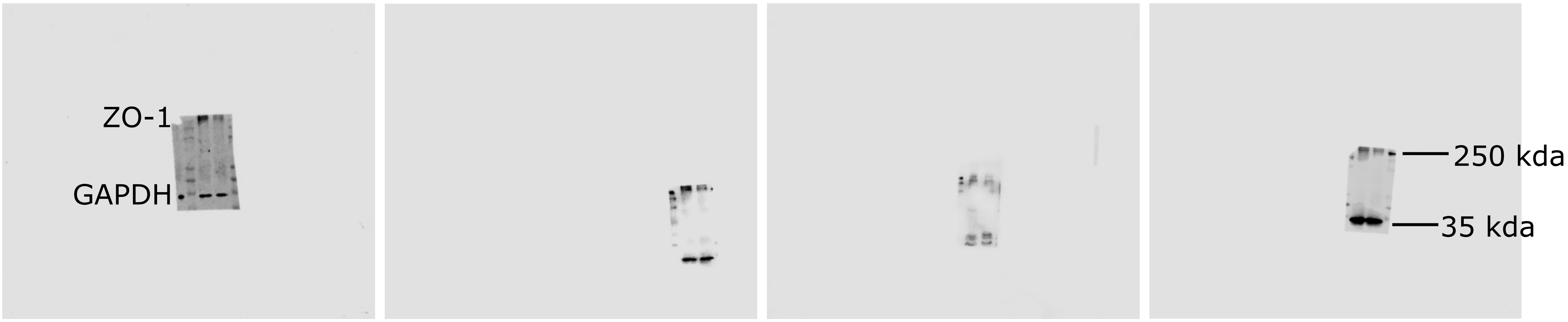

Figure 5, F

The Image captured by the LICOR instrument processed using Image Studio Ver 5.2.

Uncropped/unedited images↓

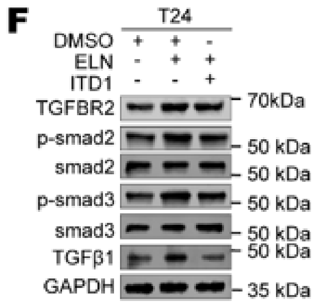

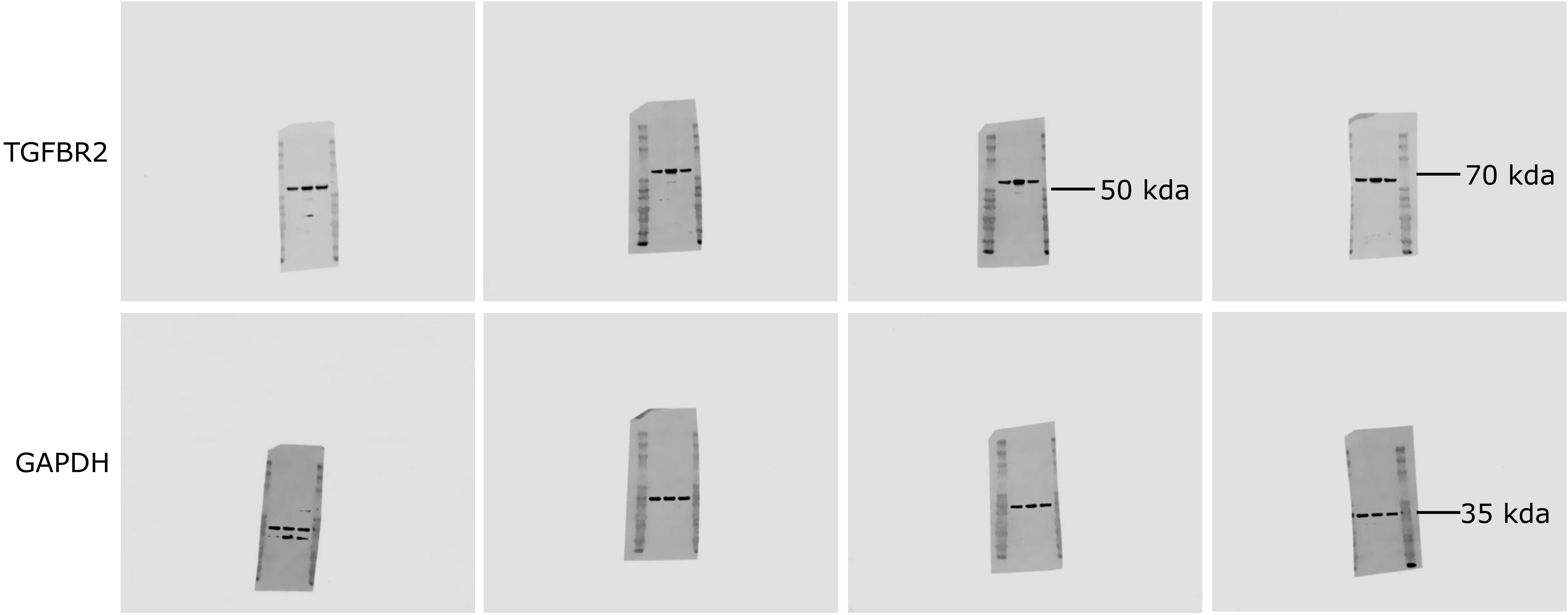

Figure 5, F

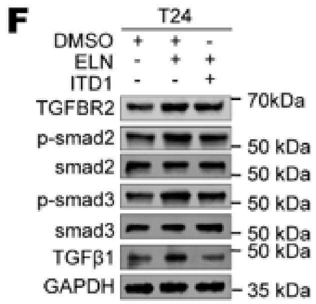

The Image captured by the LICOR instrument processed using Image Studio Ver 5.2.

Uncropped/unedited images↓

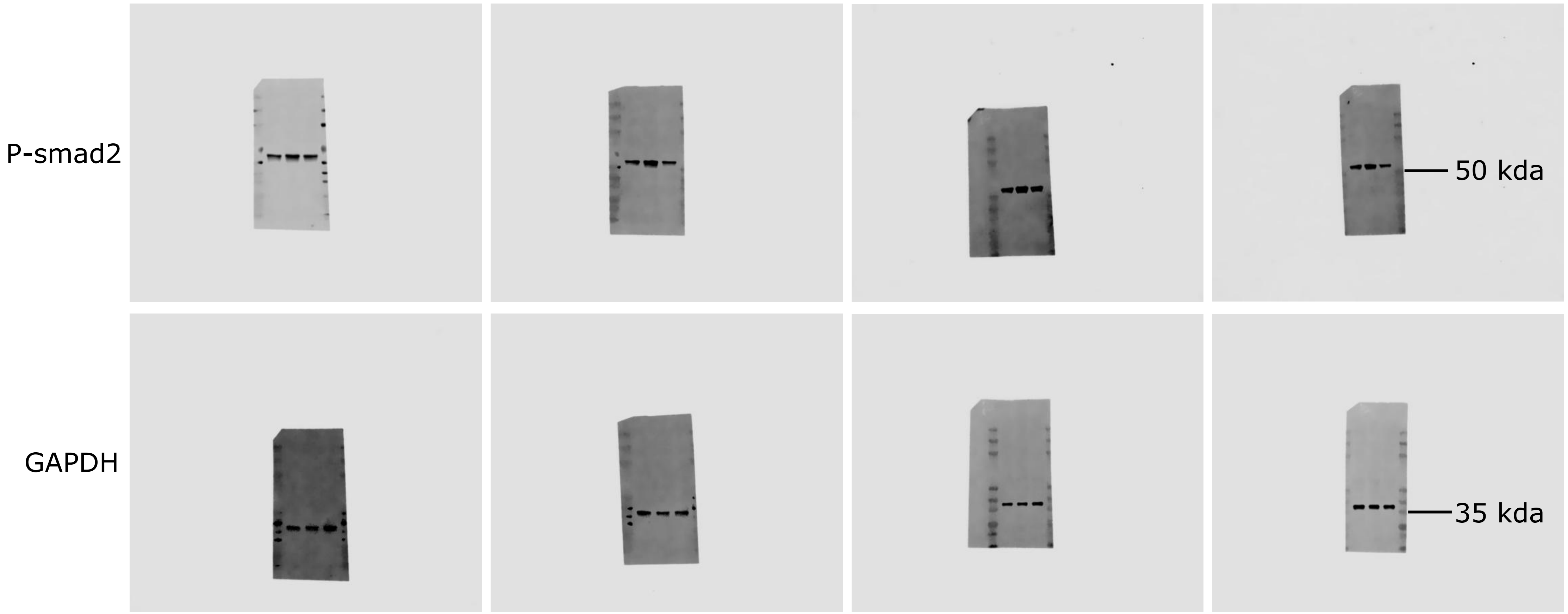

Figure 5, F

The Image captured by the LICOR instrument processed using Image Studio Ver 5.2.

Uncropped/unedited images↓

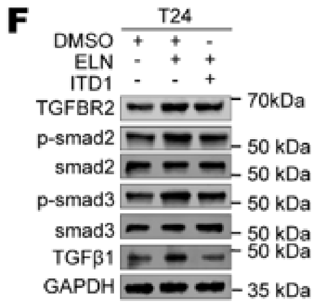

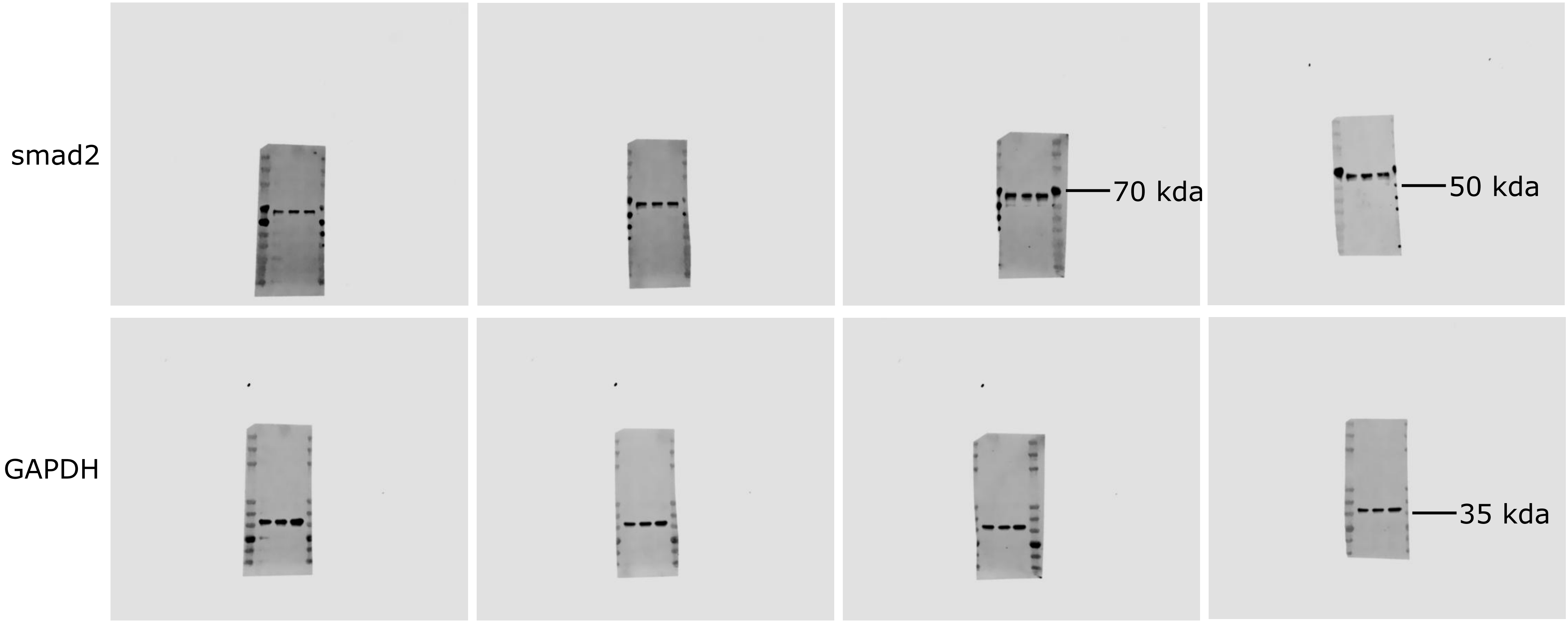

Figure 5, F

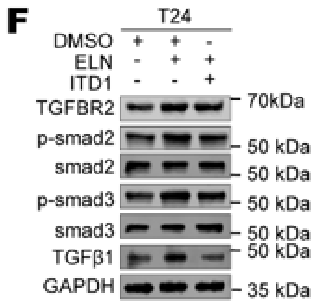

The Image captured by the LICOR instrument processed using Image Studio Ver 5.2.

Uncropped/unedited images↓

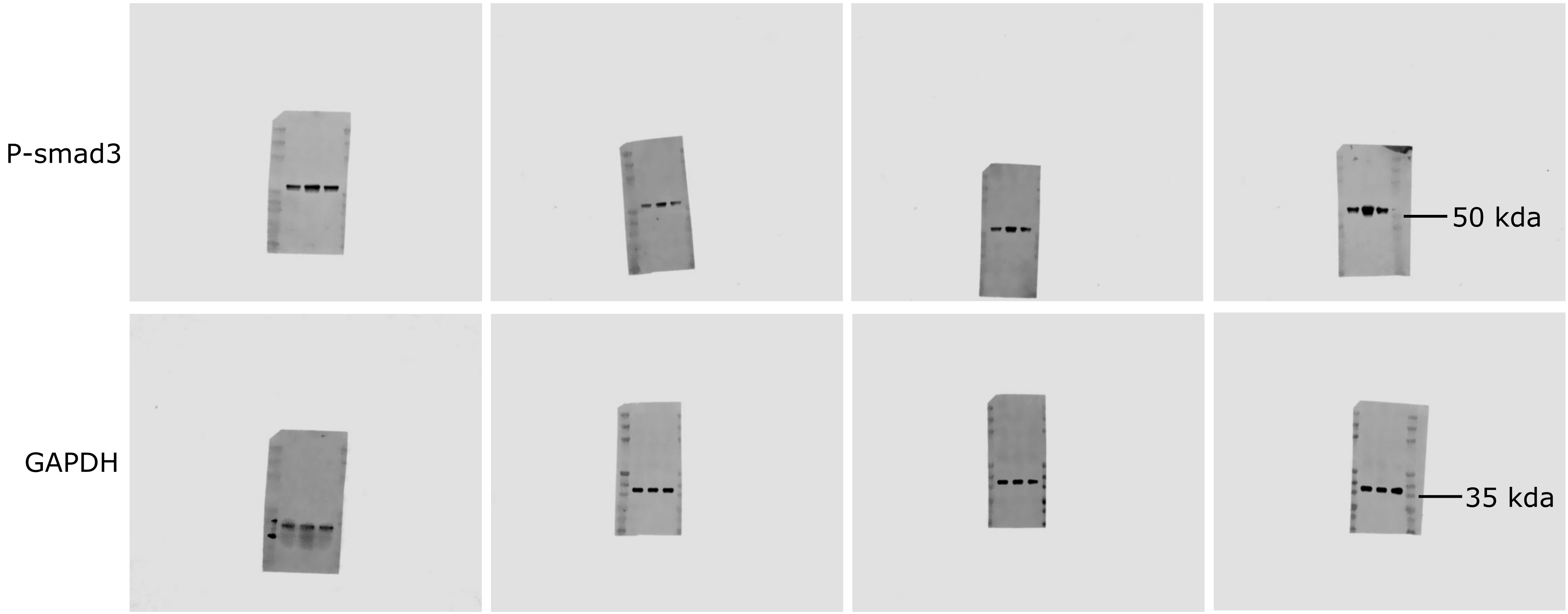

Figure 5, F

The Image captured by the LICOR instrument processed using Image Studio Ver 5.2.

Uncropped/unedited images↓

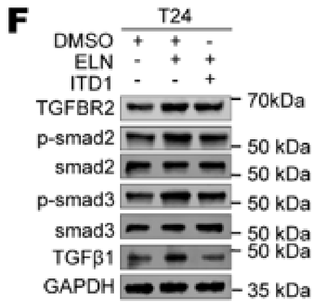

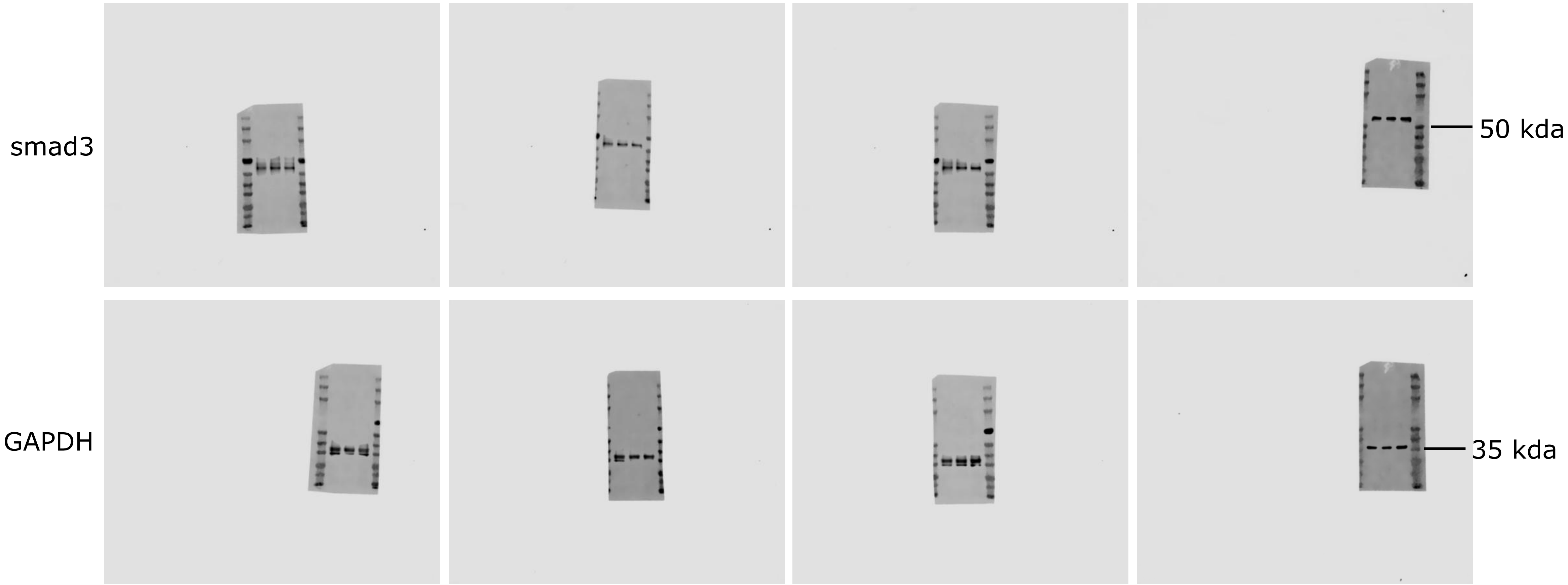

Figure 5, F

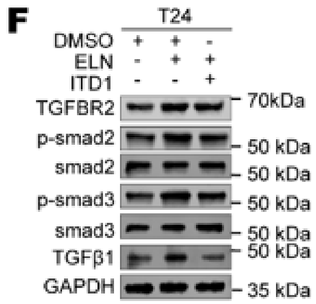

The Image captured by the LICOR instrument processed using Image Studio Ver 5.2.

Uncropped/unedited images↓

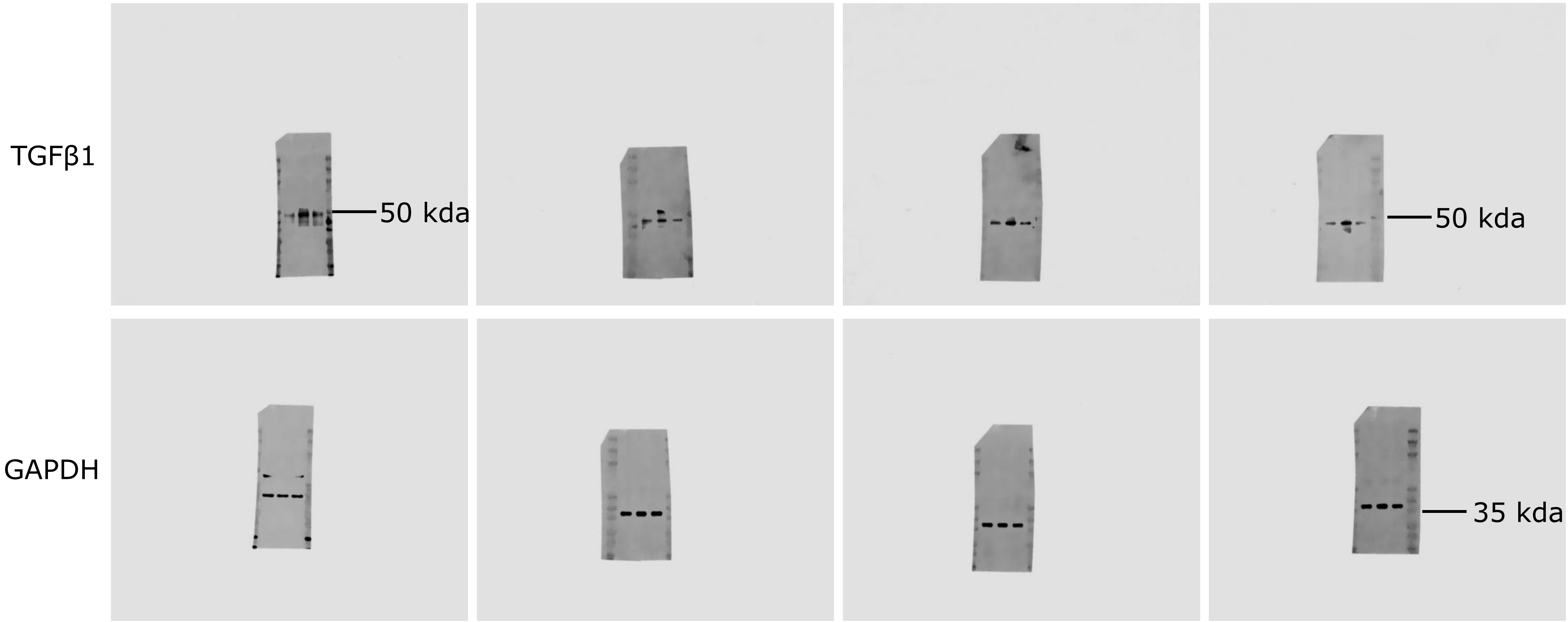

Figure 5, J

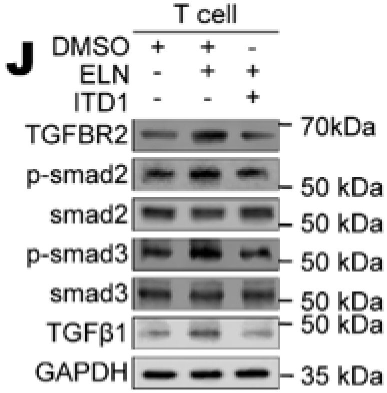

The Image captured by the LICOR instrument processed using Image Studio Ver 5.2.

Uncropped/unedited images↓

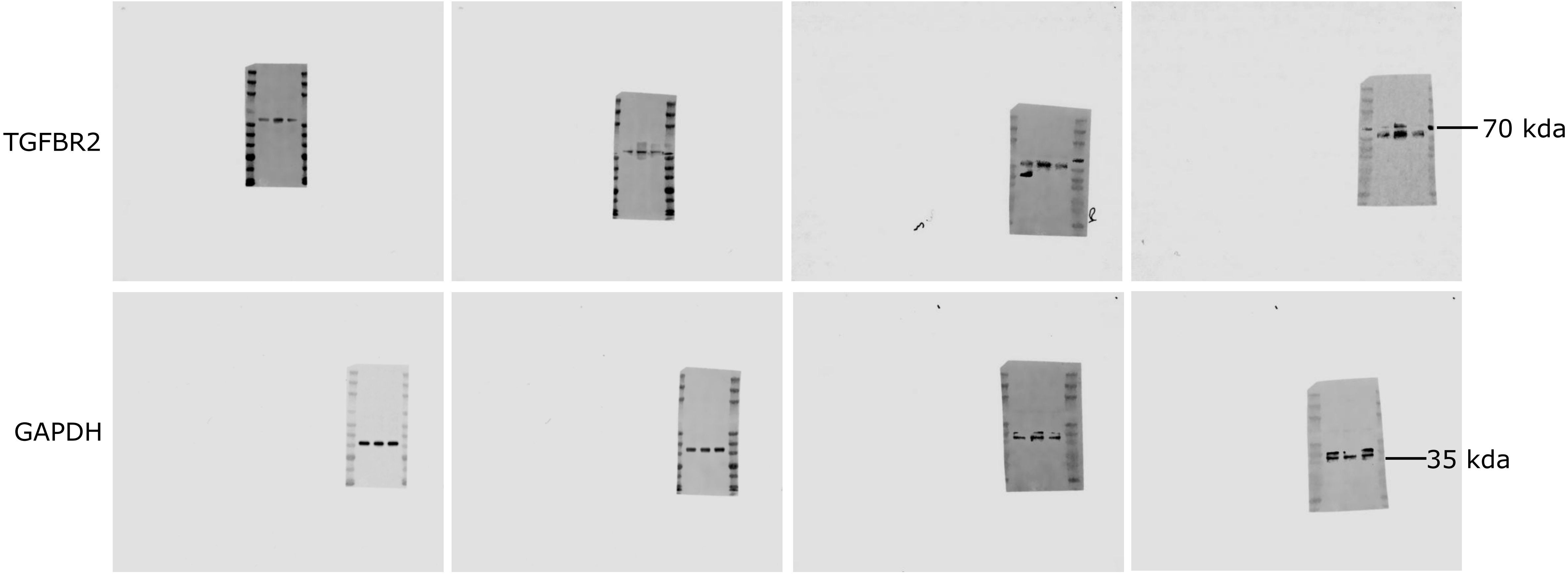

Figure 5, J

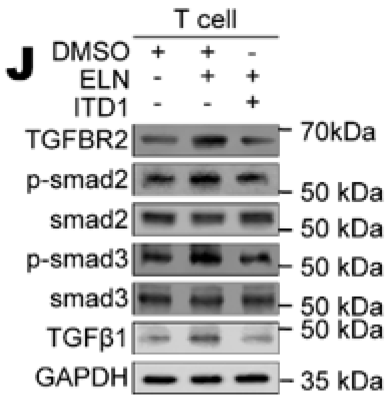

The Image captured by the LICOR instrument processed using Image Studio Ver 5.2.

Uncropped/unedited images↓

P-smad2

GAPDH

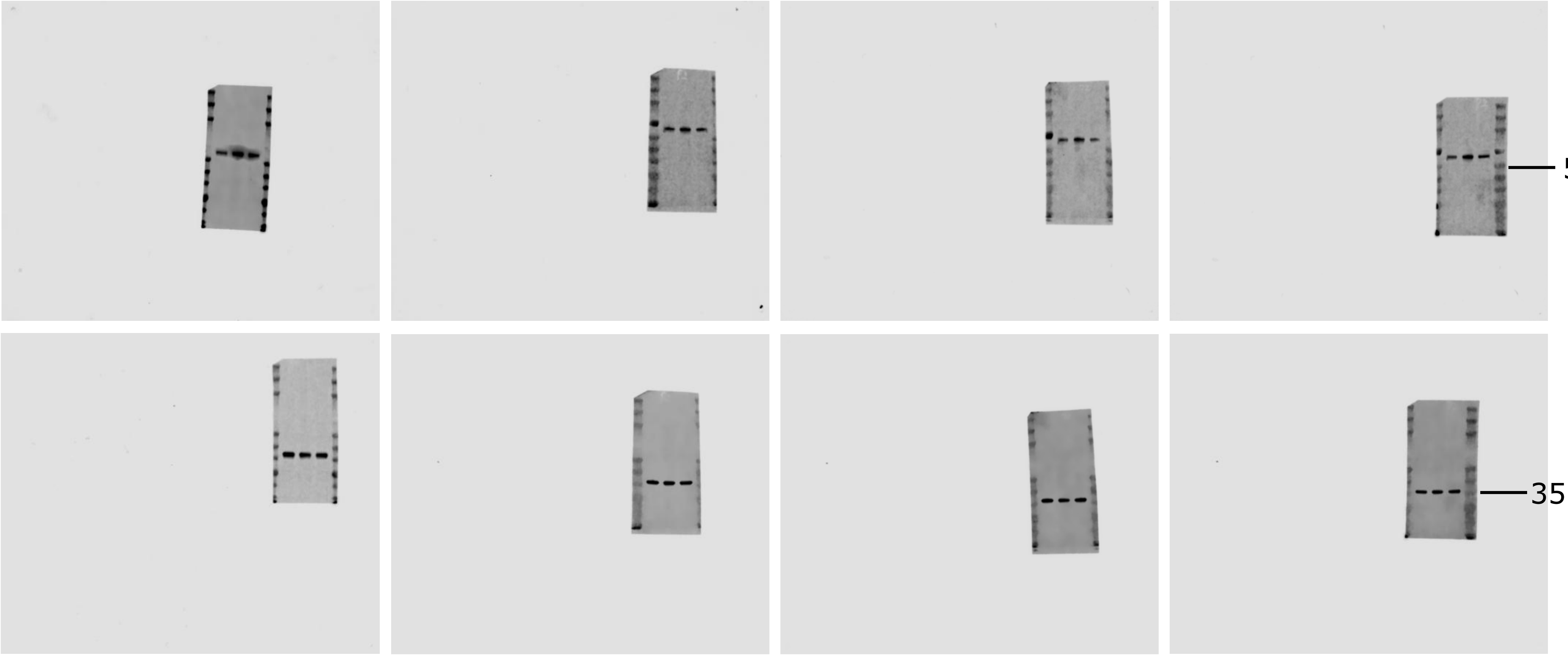

50 kda

35 kda

Figure 5, J

The Image captured by the LICOR instrument processed using Image Studio Ver 5.2.

Uncropped/unedited images↓

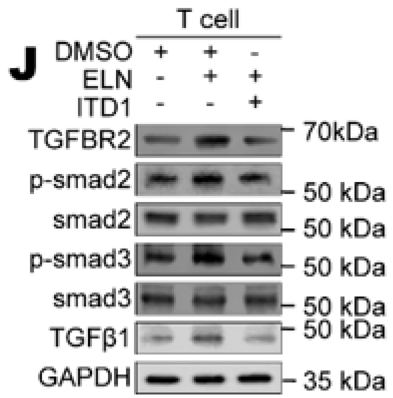

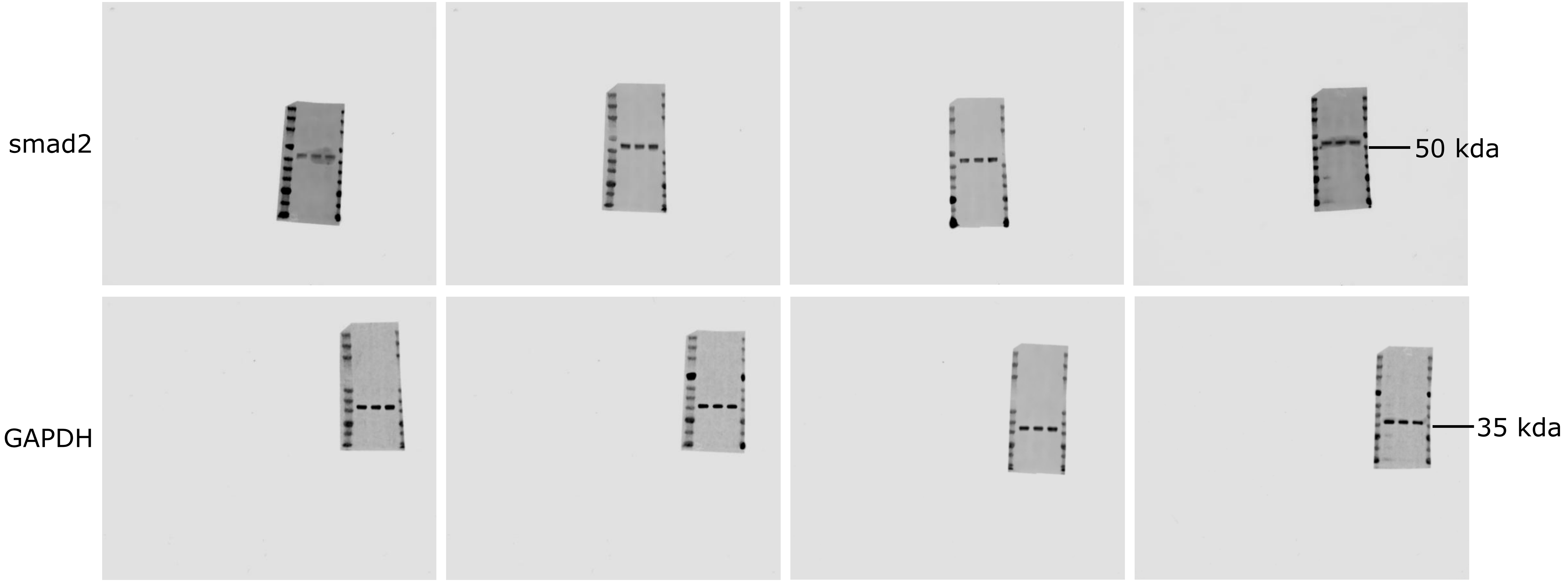

Figure 5, J

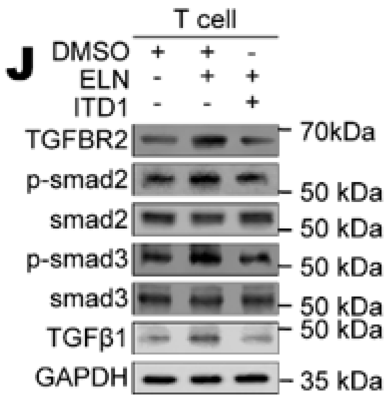

The Image captured by the LICOR instrument processed using Image Studio Ver 5.2.

Uncropped/unedited images↓

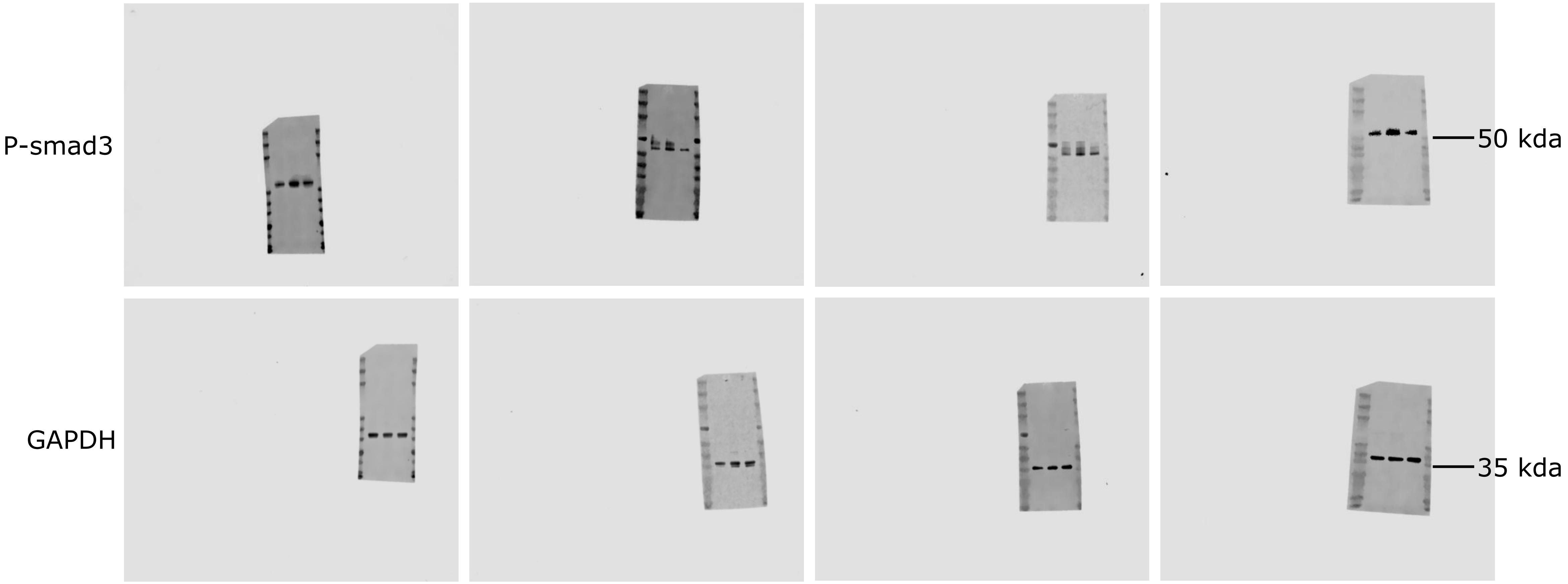

Figure 5, J

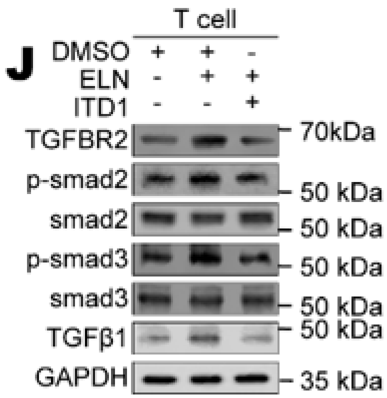

The Image captured by the LICOR instrument processed using Image Studio Ver 5.2.

Uncropped/unedited images↓

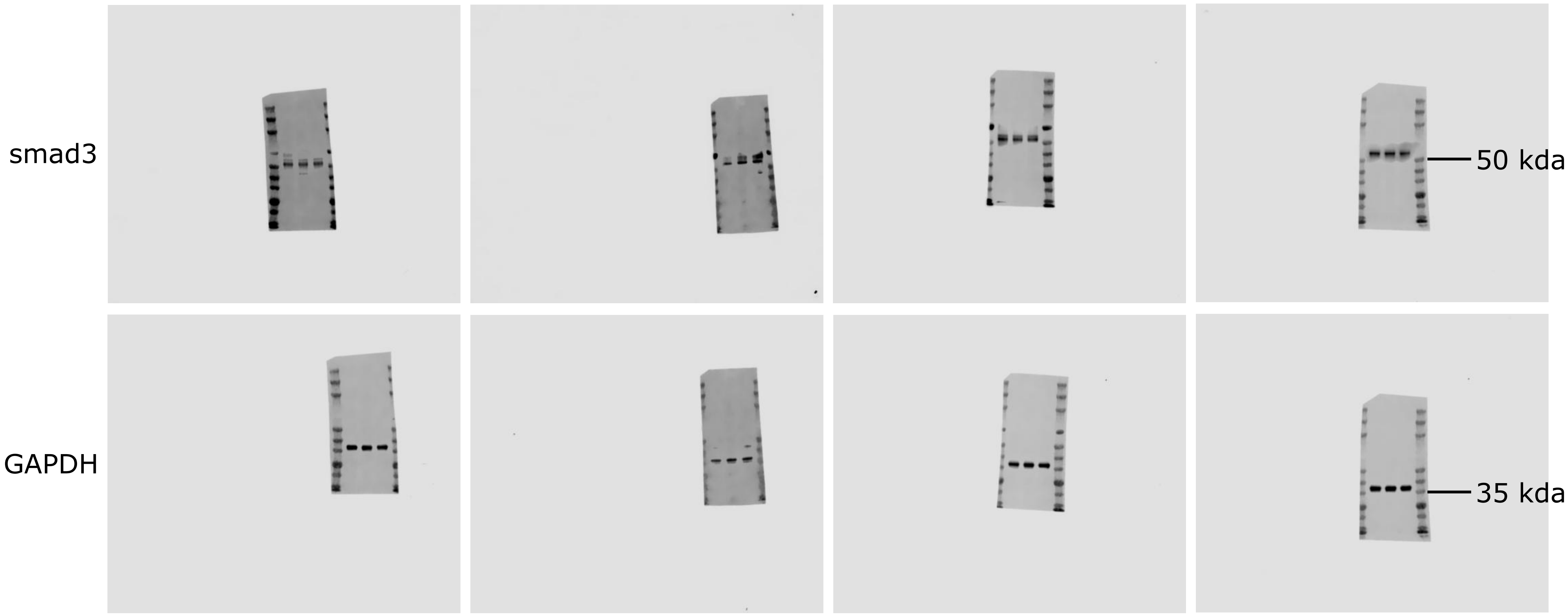

Figure 5, J

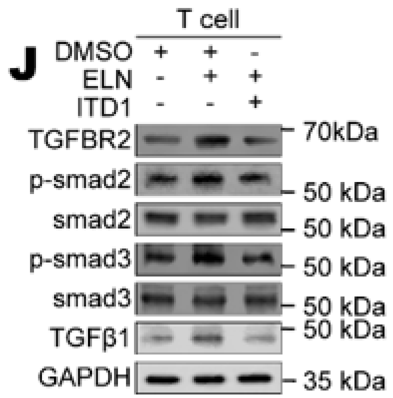

The Image captured by the LICOR instrument processed using Image Studio Ver 5.2.

Uncropped/unedited images↓

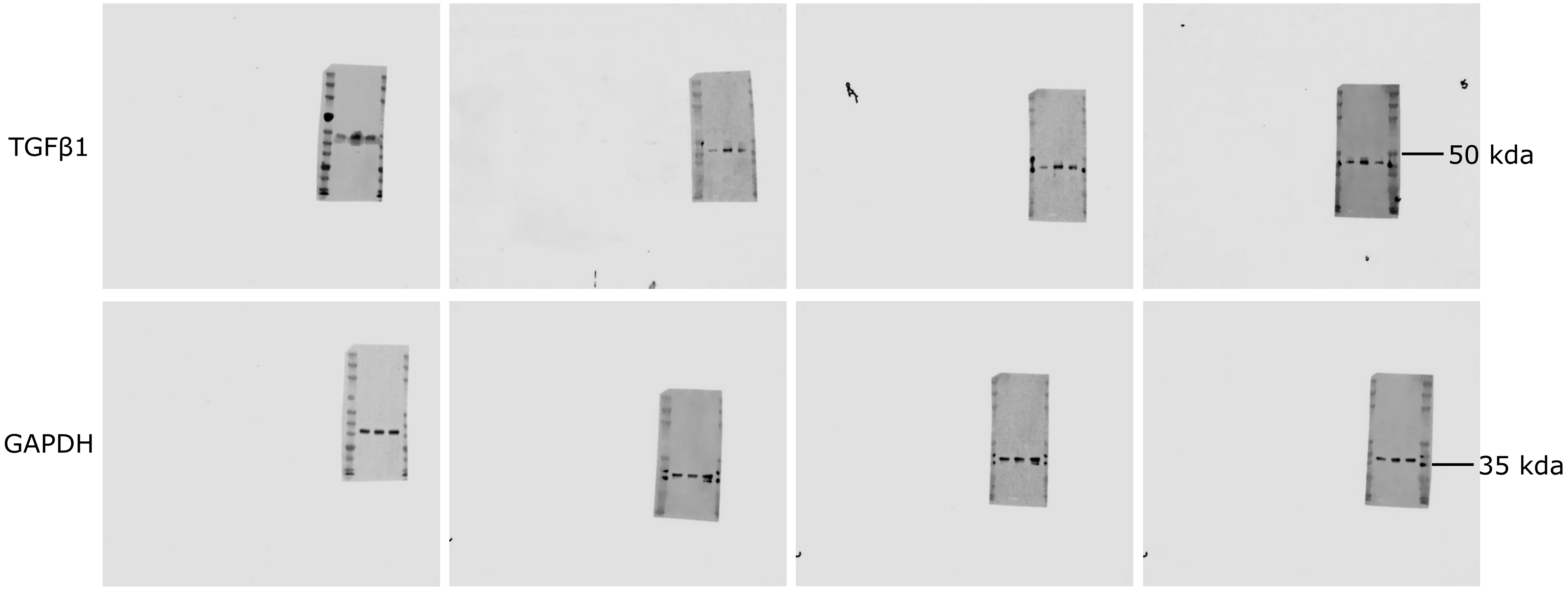

Figure 5, K

The Image captured by the LICOR instrument processed using Image Studio Ver 5.2.

Uncropped/unedited images↓

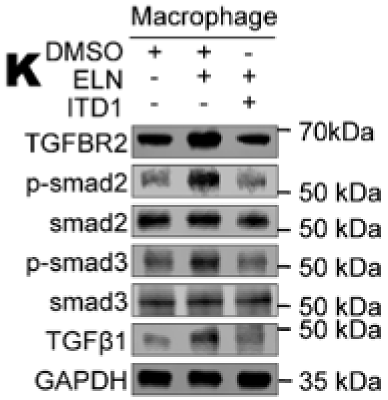

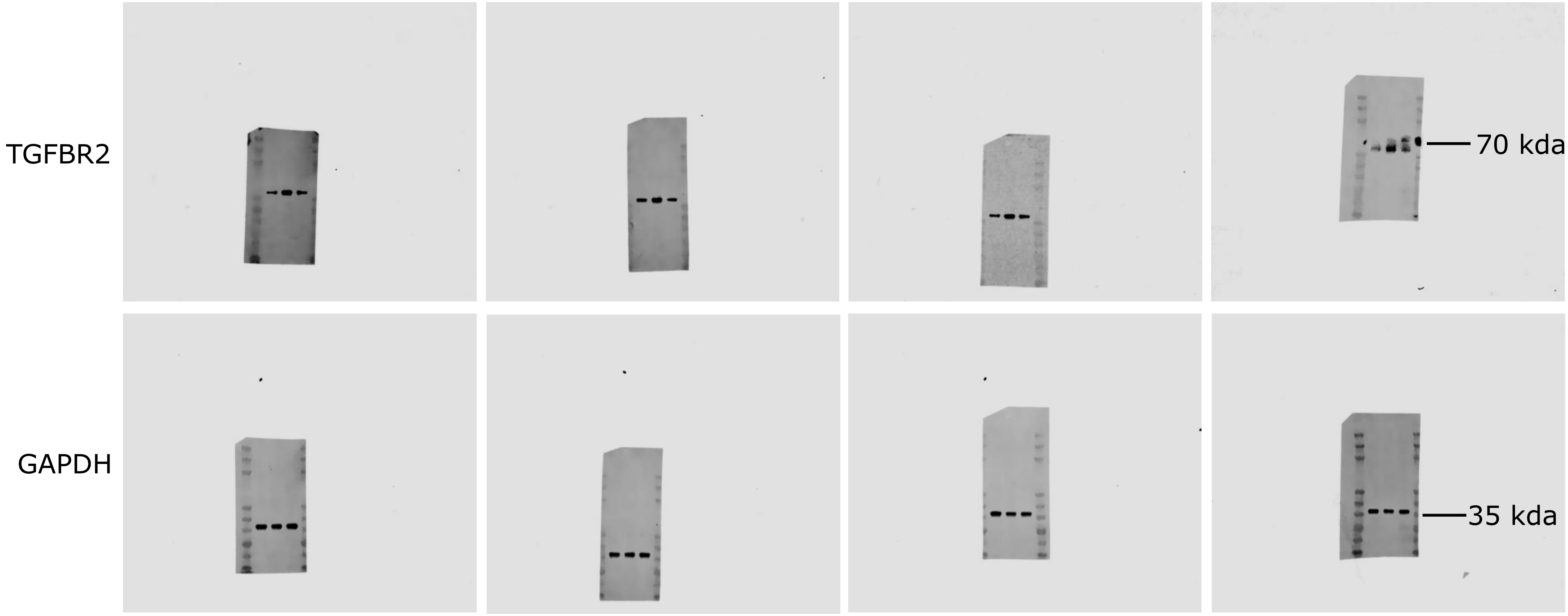

Figure 5, K

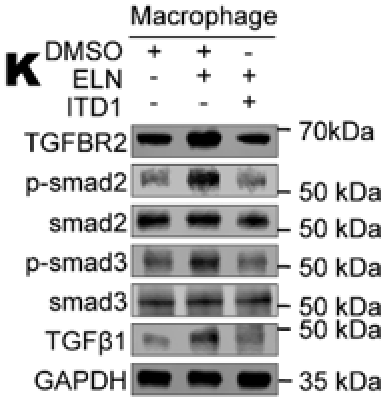

The Image captured by the LICOR instrument processed using Image Studio Ver 5.2.

Uncropped/unedited images↓

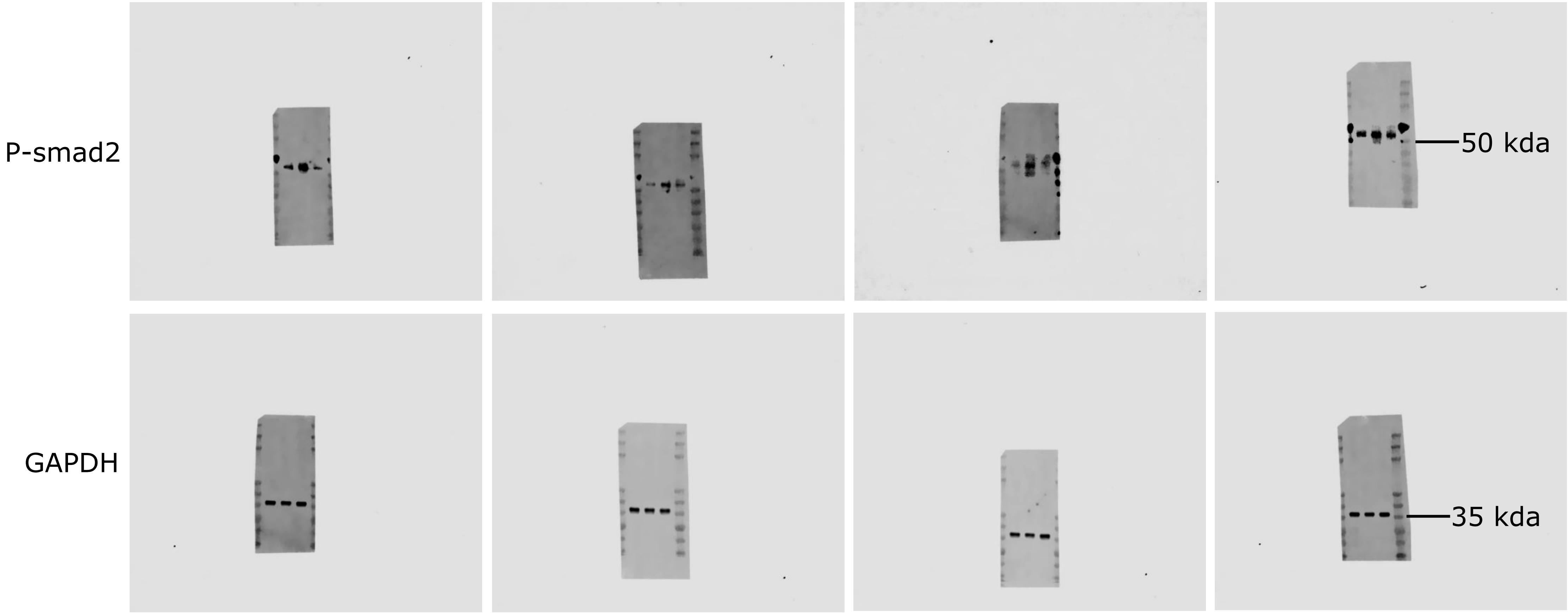

Figure 5, K

The Image captured by the LICOR instrument processed using Image Studio Ver 5.2.

Uncropped/unedited images↓

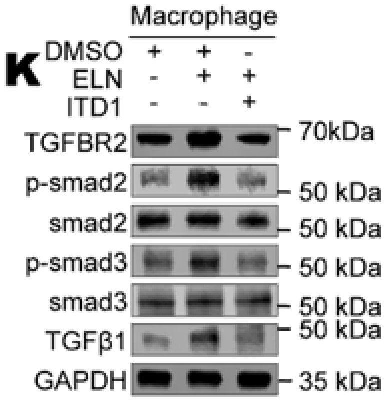

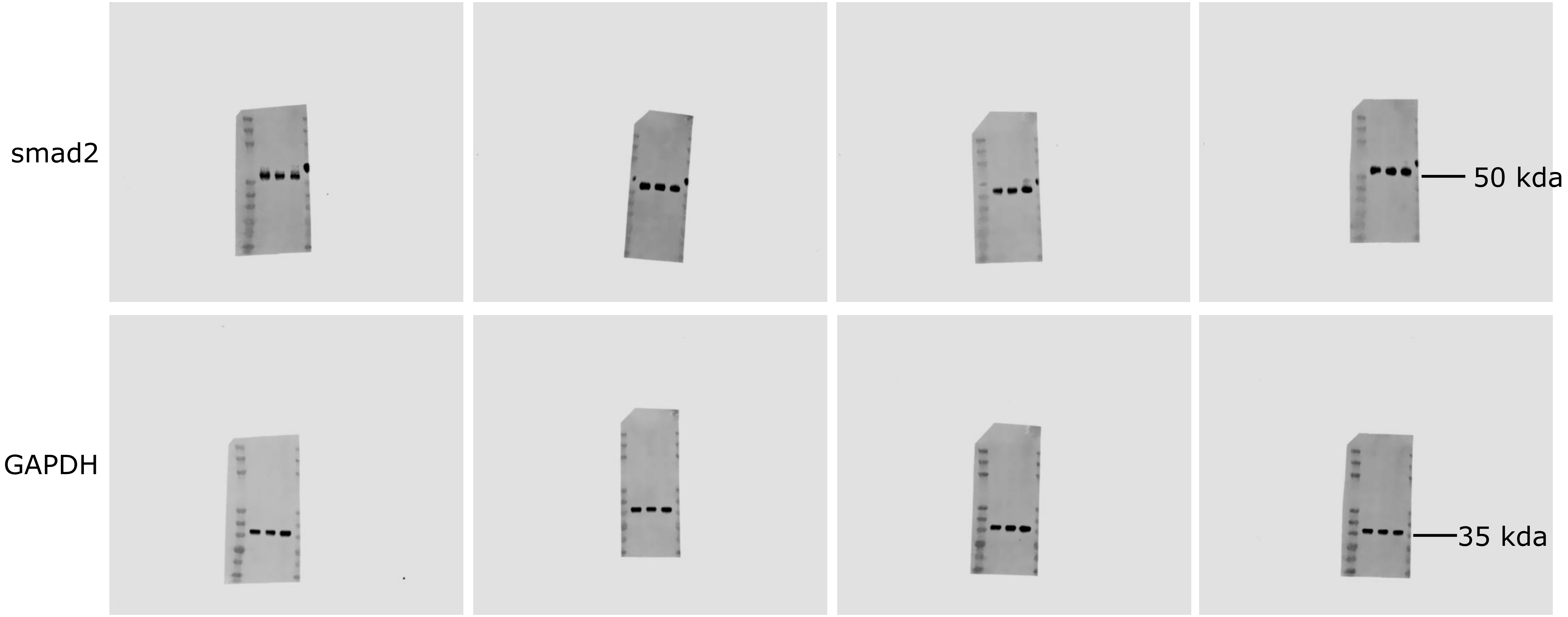

Figure 5, K

The Image captured by the LICOR instrument processed using Image Studio Ver 5.2.

Uncropped/unedited images↓

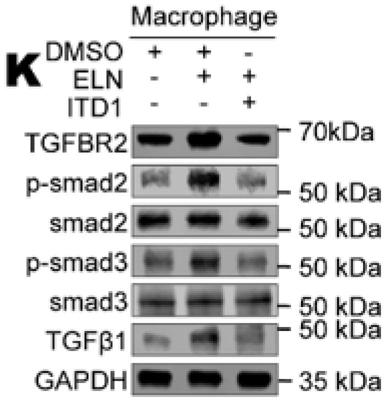

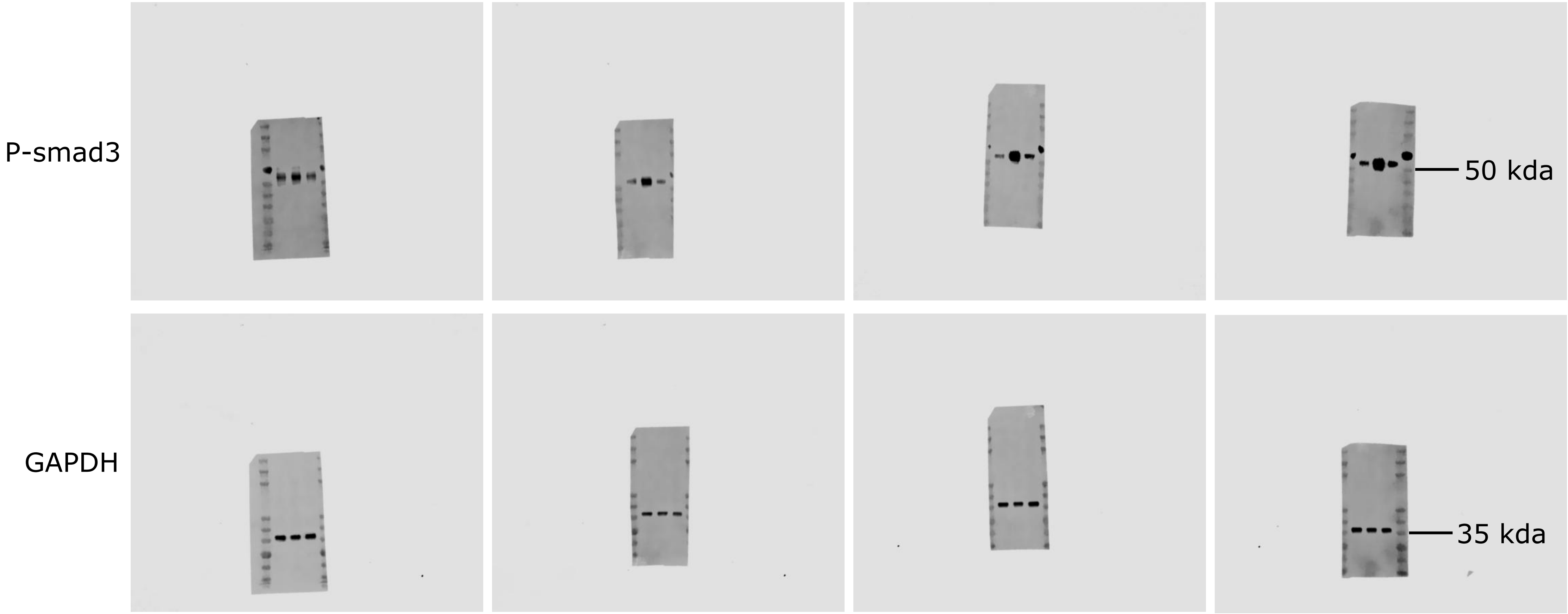

Figure 5, K

The Image captured by the LICOR instrument processed using Image Studio Ver 5.2.

Uncropped/unedited images↓

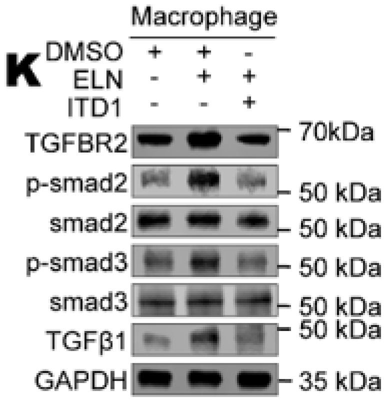

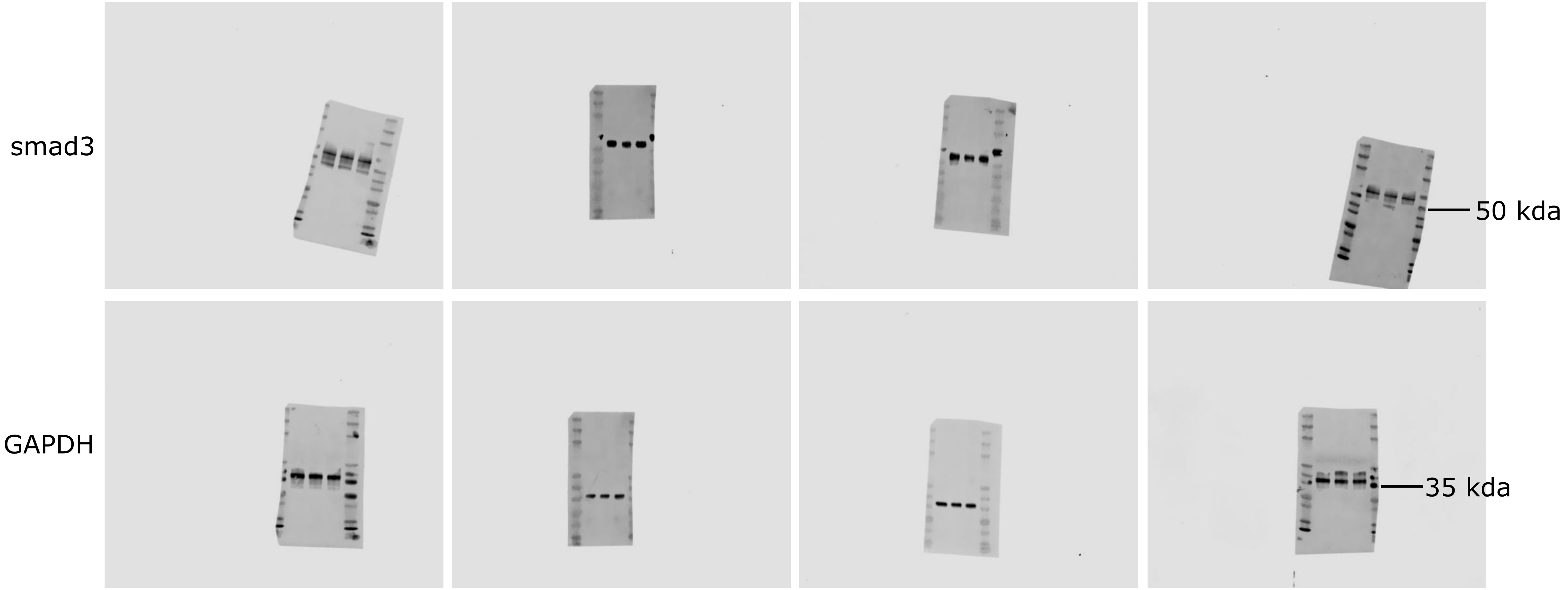

Figure 5, K

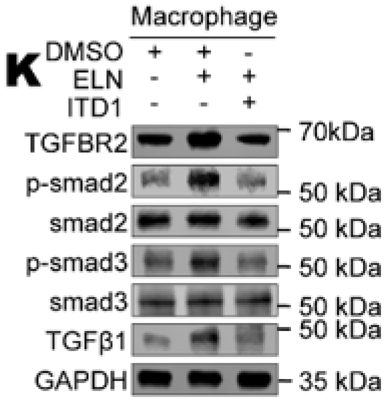

The Image captured by the LICOR instrument processed using Image Studio Ver 5.2.

Uncropped/unedited images↓

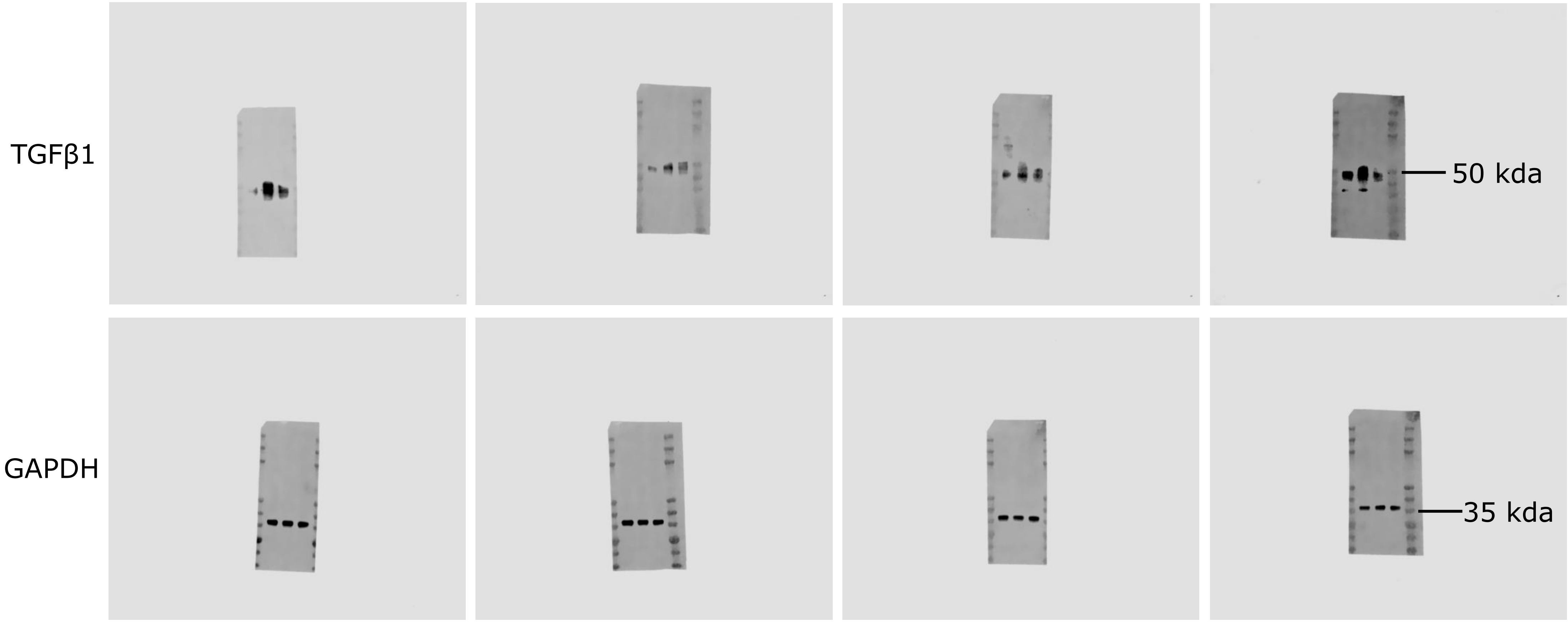

Figure S6, D

The Image captured by the LICOR instrument processed using Image Studio Ver 5.2.

Uncropped/unedited images↓

D

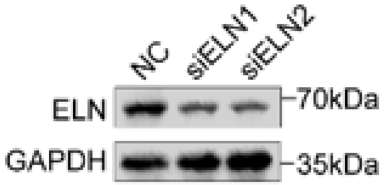

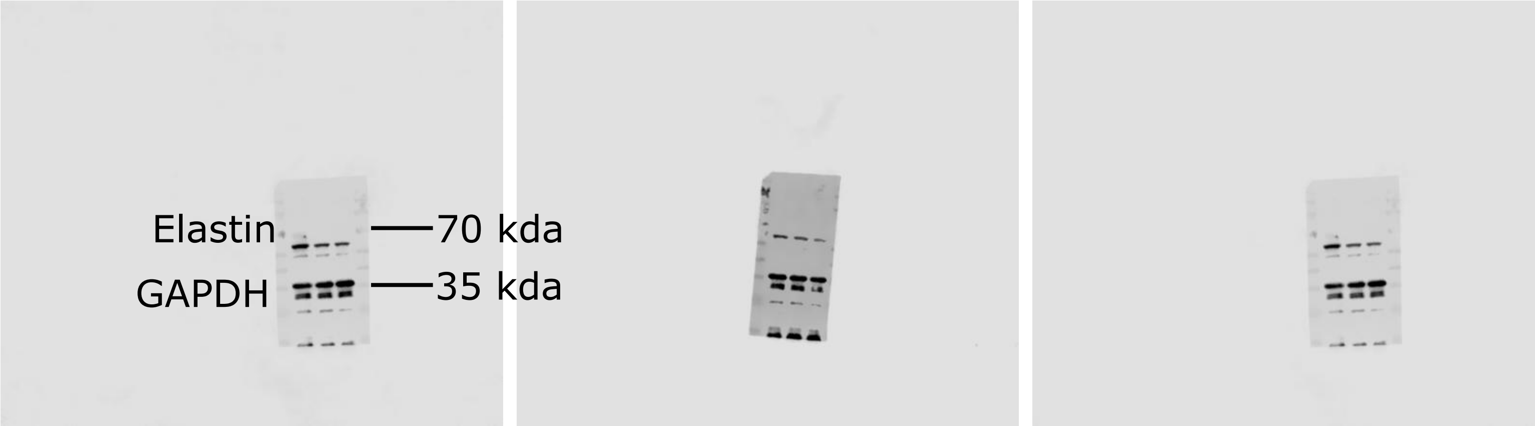

Supplement: Unedited blot and gel images [file jciinsight-11-194700-s248.pdf]
